# Modulation of Cytoskeleton, Protein Trafficking, and Signaling Pathways by Metabolites from *Cucurbitaceae*, *Ericaceae,* and *Rosaceae* Plant Families

**DOI:** 10.3390/ph15111380

**Published:** 2022-11-10

**Authors:** Ankit Patel, Aliyah Rasheed, Isiah Reilly, Zil Pareek, Mattia Hansen, Zayn Haque, Daniela Simon-Fajardo, Chloe Davies, Akash Tummala, Karlyn Reinhardt, Alexandria Bustabad, Maxwell Shaw, Jasmine Robins, Karolaent Vera Gomez, Thitisuda Suphakorn, Mariana Camacho Gemelgo, Ashley Law, Kristina Lin, Elizabeth Hospedales, Harrison Haley, Jean Pierre Perez Martinez, Saifullah Khan, Jessica DeCanio, Malcolm Padgett, Artem Abramov, Meera Nanjundan

**Affiliations:** Department of Cell Biology, Microbiology and Molecular Biology, University of South Florida, 4202 East Fowler Avenue, Tampa, FL 33620, USA

**Keywords:** *Ericaceae*, *Rosaceae*, *Cucurbitaceae*, cytoskeleton, protein trafficking, signaling

## Abstract

One promising frontier within the field of Medical Botany is the study of the bioactivity of plant metabolites on human health. Although plant metabolites are metabolic byproducts that commonly regulate ecological interactions and biochemical processes in plant species, such metabolites also elicit profound effects on the cellular processes of human and other mammalian cells. In this regard, due to their potential as therapeutic agents for a variety of human diseases and induction of toxic cellular responses, further research advances are direly needed to fully understand the molecular mechanisms induced by these agents. Herein, we focus our investigation on metabolites from the *Cucurbitaceae, Ericaceae,* and *Rosaceae* plant families, for which several plant species are found within the state of Florida in Hillsborough County. Specifically, we compare the molecular mechanisms by which metabolites and/or plant extracts from these plant families modulate the cytoskeleton, protein trafficking, and cell signaling to mediate functional outcomes, as well as a discussion of current gaps in knowledge. Our efforts to lay the molecular groundwork in this broad manner hold promise in supporting future research efforts in pharmacology and drug discovery.

## 1. Introduction

### 1.1. Objective of Review

For approximately 60,000 years, plants have been utilized across the world for treating a diverse array of health conditions and diseases [1]. Indeed, plants are used not only as a medicinal source, but their metabolites also provide the foundation for developing new chemotherapies [1]. For example, a metabolite from the *Apocynaceae* plant family (i.e., vinblastine, a monoterpenoid indole alkaloid from *Catharanthus roseus*) elicits anti-neoplastic properties and is an FDA-approved drug for use in patients afflicted by various cancers such as non-Hodgkin lymphomas [2]. Along with the goal of identifying bioactive metabolites from plants, their subsequent synthetic/semi-synthetic development would ideally minimize toxicity and improve efficacy for use in patients [1]. However, to generate sufficient industrial-level quantities of vinblastine, for example, necessary for clinical use, its production has been based on extracting low yields followed by purification of precursors, chemical coupling, and reduction reactions [3]. Since up to 2 tons of leaves are needed to obtain 1 g of the final product, vinblastine has unfortunately been listed as a drug with supply chain issues by the FDA in recent years [3]. In spite of the numerous challenges [1], this prior work sets a strong framework for future synthesis platforms (i.e., using engineered microbial hosts [3]) and clinical application of promising metabolites derived from other plant families.

Herein, our objective is to critically evaluate the literature with the identification of gaps in knowledge concerning the mechanisms of action that underlie the pharmacological potential of plant metabolites across a subset of plant families in a comparative manner. Specifically, we evaluate their effects on the cytoskeleton, protein trafficking (i.e., intracellular protein movement), and signal transduction (i.e., alterations in EGFR, MAPK, PI3K/AKT, JAK/STAT, GPCR signaling), which may consequently contribute to functional outcomes that target disease manifestations. Therefore, it is necessary to increase our knowledge of the regulation of plant metabolites on the cytoskeleton, protein trafficking, and signaling pathways in relation to various diseases.

### 1.2. Relevance to Hillsborough County in the State of Florida and Selection of Plant Families

A common discussion topic in the field of ethnobotany includes factors that underlie the selection of specific plants for use as medicines; these include their abundance, ease of access, and affiliation with a taxonomy [4]. Interestingly, medicinally important plants are only partly overlapping across various regions of the world, with seven plant families (namely *Apocynaceae*, *Apiaceae*, *Euphorbiaceae*, *Leguminosae*, *Lamiaceae, Malvaceae*, and *Ranunculaceae*) being reported worldwide as the most medicinally valuable [5]. In contrast, in North America, five plant families (namely *Asteraceae*, *Apiaceae*, *Ericaceae*, *Ranunculaceae*, and *Rosaceae*) were identified as species-rich with high medicinal value [6]. This contrasts with findings in other countries and specific regions in North America. For example, in Hawaii, beyond the above-listed families, *Cyperaceae* and *Poaceae* were identified with high medicinal value [7]. In another published study, 21 plant families (including *Ericaceae* and *Rosaceae*) were identified as medicinally important in at least four countries [8]. 

**Figure 1:**
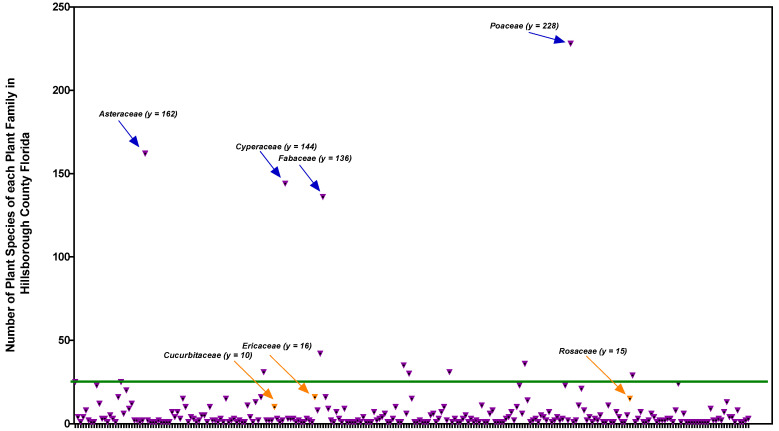
The number of plant species of each plant family identified in Hillsborough County, Florida, United States. The triangles represent plant families; the blue arrows point to plant families with the highest number of species; the orange arrows point to plant families selected in this article.

In Hillsborough County within the state of Florida, we identified 251 plant families with a total of 1937 species using the Florida Plant Atlas publicly-accessible database [9]. As shown in Figure 1, the plant families with the highest number of plant species include *Poaceae* (228), *Asteraceae* (162), *Cyperaceae* (144), and *Fabaceae* (136). However, towards our review objective and for comparative purposes, we selected three plant families that had at least 10 species in Hillsborough County. Two families are associated with high medicinal value in North America (namely, *Ericaceae* (16) and *Rosaceae* (15)), and one is associated with a lesser medicinal value (*Cucurbitaceae* (10), although reported to be of high medicinal value in Nepal and Italy [8]). *Ericaceae* and *Rosaceae* are also associated with high food value, particularly the fruit component [6]. Another plant family with high food value from the fruit component includes the *Cucurbitaceae* family [6], which is equivalently abundant to *Ericaceae* and *Rosaceae* in terms of the number of species in Hillsborough county. Although the *Cucurbitaceae* was not listed as an important medicinal plant family in North America [8], it does have a higher-than-average number of uses for both foods and drugs, similar to *Ericaceae* and *Rosaceae*. For these collective reasons, these three plant families were pursued the study performed herein.

### 1.3. Methods—Search Terms and Pubmed Results

We exclusively utilized PubMed to identify primary research articles for the critical evaluation of the cytoskeleton, protein trafficking, and signaling pathways. From the search results, the abstracts were first reviewed, and those that were irrelevant were eliminated. It was further noted that there were multiple articles that overlapped across the various search terms utilized. The full primary research articles were accessed through our institution and reviewed; those that were irrelevant were also discarded upon review. We utilized similar search terms for each plant family; the number of identified articles is indicated in Supplementary Table S1 (*Cucurbitaceae*), Supplementary Table S2 (*Ericaceae*), and Supplementary Table S3 (*Rosaceae*). Out of all these search results, >200 primary research articles were reviewed comprehensively. 

Specific search terms for the *Cucurbitaceae* family included “cucurbitacin human motor protein”, “cucurbitacin human dynein”, “cucurbitacin human kinesin”, cucurbitacin human microtubules”, “cucurbitacin human actin”, “cucurbitacin human vimentin”, “cucurbitacin human lamin”, “cucurbitacin human golgi”, “cucurbitacin human endoplasmic reticulum”, “cucurbitacin human lysosome”, “cucurbitacin human MAPK”, “cucurbitacin human AKT”, “cucurbitacin human PI3K”, “cucurbitacin human JAK”, “cucurbitacin human STAT”, “cucurbitacin human EGFR”, “cucurbitacin human adenylyl cyclase”, “cucurbitacin human phospholipase C”, and “cucurbitacin human GPCR.” 

Specific search terms for the *Ericaceae* family included “Ericaceae human motor protein”, “Ericaceae human dynein”, “Ericaceae human kinesin”, “Ericaceae human microtubules”, “Ericaceae human actin”, “Ericaceae human vimentin”, “Ericaceae human lamin”, “Ericaceae human golgi”, “Ericaceae human endoplasmic reticulum”, “Ericaceae human lysosome”, “Ericaceae human MAPK”, “Ericaceae human AKT”, “Ericaceae human PI3K”, “Ericaceae human JAK”, “Ericaceae human STAT”, “Ericaceae human EGFR”, “Ericaceae human adenylyl cyclase”, “Ericaceae human phospholipase C”, “Ericaceae human GPCR”.

Specific search terms for the *Rosaceae* family included “Rosaceae human motor protein”, “Rosaceae human dynein”, “Rosaceae human kinesin”, “Rosaceae human microtubules”, “Rosaceae human actin”, “Rosaceae human vimentin”, “Rosaceae human lamin”, “Rosaceae human golgi”, “Rosaceae human endoplasmic reticulum”, “Rosaceae human lysosome”, “Rosaceae human MAPK”, “Rosaceae human AKT”, “Rosaceae human PI3K”, “Rosaceae human JAK”, “Rosaceae human STAT”, “Rosaceae human EGFR”, “Rosaceae human adenylyl cyclase”, “Rosaceae human phospholipase C”, “Rosaceae human GPCR”.

We also direct the reader to additional, comprehensive review articles for these plant families or specific species that may present other valuable information at a broader scope. 

## 2. Overview of Plant Families: *Cucurbitaceae*, *Ericaceae*, and *Rosaceae*

### 2.1. Plant Background

#### 2.1.1. Plant Background on Cucurbitaceae

Plant genera from the gourd family *Cucurbitaceae* that we have identified in Hillsborough County, Florida, using the Florida Plant Atlas [9], are summarized in Table 1. Together with the presence of multiple phytochemicals (i.e., carotenoids, glycosides, saponins, steroids, tannins, terpenoids, and resins) [10], members of the *Cucurbitaceae* family are rich in cucurbitacins (and their derivatives) that are triterpenoids, which elicit pheromone activities to mediate plant self-protection from external injury [11]. The structural details of the major cucurbitacins are summarized in [12] and are diverse due to side chain groups, stereochemistry, and the presence of functional groups on specific rings [12]. Although cucurbitacins can be found in all plant parts (i.e., leaves, stems, flowers, fruits, seeds, and roots), they are enriched in mature plant fruits and roots [12]. Cucurbitacins elicit an array of biological activities, including anti-inflammatory, anti-atherosclerotic, anti-diabetic, and anti-neoplastic responses [12]. 

#### 2.1.2. Plant Background on Ericaceae

Table 2 summarizes the plant genera from the heath family *Ericaceae* in our Florida county, using the Florida Plant Atlas [9]. Not only is this family of high medicinal value across multiple world regions, but it is highly important to Florida’s economy [13]. Some of the phytochemicals identified in the *Ericaceae* family include anthocyanins (derived from fruits (outer layer with a minor component in seeds and pulp)) and phenolic compounds (proanthocyanidins, flavonols, and hydroxycinnamic acids) [14]. It is important to note that the effectiveness of anthocyanins *in vivo* is regulated by their bioavailability as a result of their degradation [14]. Within the proanthocyanidin class, there are several common members, including cyanidin, delphinidin, malvidin, pelargonidin, petunidin, and peonidin. There are also over 35 anthocyanin glycosides [14]. Flavonoids are also abundant; of these, quercetin is the most common, together with kaempferol, myricetin, and several glycosidic derivatives [14]. A compilation of compounds in this plant family has tabulated >300 phytochemicals [15]. The extracts from this plant family and/or a few purified compounds exhibit an array of biological activities such as anti-microbial, anti-neoplastic, anti-inflammatory, and anti-diabetic, as well as protection against neurodegenerative and cardiovascular diseases [15].

#### 2.1.3. Plant Background on Rosaceae

The highly medicinally rich flowering plant family, *Rosaceae*, comprises over 100 genera [16], of which several are present within Hillsborough County, Florida (Table 3) [9]. Similar to the *Ericaceae* family, it is also economically important as valuable crop for fruit consumption as well as the production of perfume and cosmetics. The *Rosaceae* family contains a variety of phytochemicals; within the red raspberry from *Rubus idaeus* L, the bioactive compounds are classified based on their structural similarities, including phenolic acids and complex polyphenols, amongst others [17]. With respect to polyphenols, the major compound groups include anthocyanins (9 components in red raspberries) and ellagitannins (i.e., sanguiin H-6 in red raspberries), as well as other phenolics such as hydroxycinnamic acids (i.e., caffeic, *p*-coumaric, and ferulic acids), hydroxybenzoic acids (i.e., ellagic and *p*-hydroxybenzoic acids), flavonols (i.e., quercetin and kaempferol), and tannins [17]. Many of these phytochemicals, such as the *Ericaceae* family, are also subjected to their bioavailability due to their degradation *in vivo* [17]. The extracts and/or purified phytochemicals from species from *Rosaceae* induce anti-neoplastic, anti-inflammatory, and anti-diabetic responses in addition to protection against obesity and neurodegenerative diseases [17].

### 2.2. Overview of Plant Metabolite Type Use (Pure versus Mixture) and Human Disease Focus in the Reviewed Literature

Based on our search terms, in the majority of the research literature reviewed for *Cucurbitaceae*, highly purified agents purchased from companies were utilized in the studies described herein. This is in contrast to the *Ericaceae* and *Rosaceae* families, in which the majority of studies utilized plant extracts and/or fractions, with only a subset utilizing purified compounds. Supplementary Files S1–S3 (*Cucurbitaceae*, *Ericaceae*, and *Rosaceae*, respectively) summarize the use of these agents as either purified, mixed, or fractionated components together with originating plant details if provided. 

**Figure 2:**
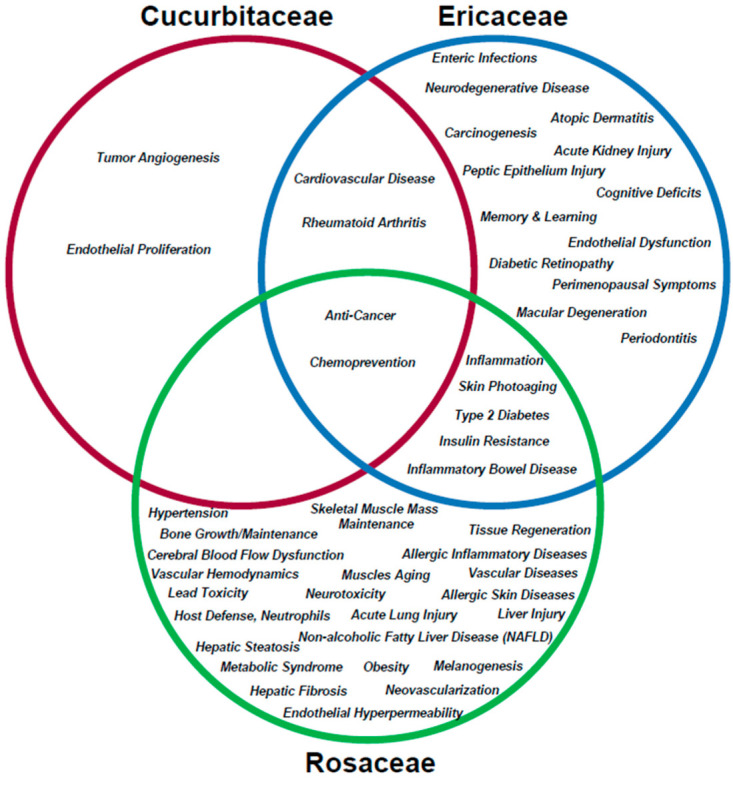
A Venn-diagram representation of the diseases in which metabolites from each plant family have been reported, with respect to the PubMed, searches performed.

Furthermore, based on our focused search term analyses, we present similarities and differences amongst these three plant families in terms of research area foci to specific human diseases; the results show higher diversity in human diseases within the *Rosaceae* family in contrast to the *Cucurbitaceae* family, which appears to be mostly focused on anti-neoplastic responses. These results are presented in Supplementary File S4 and schematically represented as a Venn diagram in Figure 2. 

## 3. Phytochemical-Induced Alterations on the Cytoskeleton 

### 3.1. Importance of the Cytoskeleton

Multiple cellular events (i.e., angiogenesis, migration, invasion, cell division, and intracellular protein trafficking) require dynamic cytoskeletal rearrangements involving changes in microtubules, actin, and intermediate filaments, amongst other regulatory components [18]. Hallmarks of cancer include dissemination from the primary tumor site followed by re-establishment at a secondary site and increased cellular proliferative capacity. These events are essential to support tumor growth, angiogenesis, and metastasis, for which the latter involves cell invasion and migration [18,19]. Drugs that disrupt the cytoskeleton (i.e., vinblastine) are utilized in the clinic as a treatment strategy for cancer. With an improved understanding of the mechanisms underlying cytoskeletal regulation, improved targeting agents could be designed to improve patient survival [18]. Please see Table 4 for a summary of plant components investigated with respect to health relevance or associated disease models. 

### 3.2. Effect of Cucurbitaceae Phytochemicals on the Cytoskeleton

**Figure 3:**
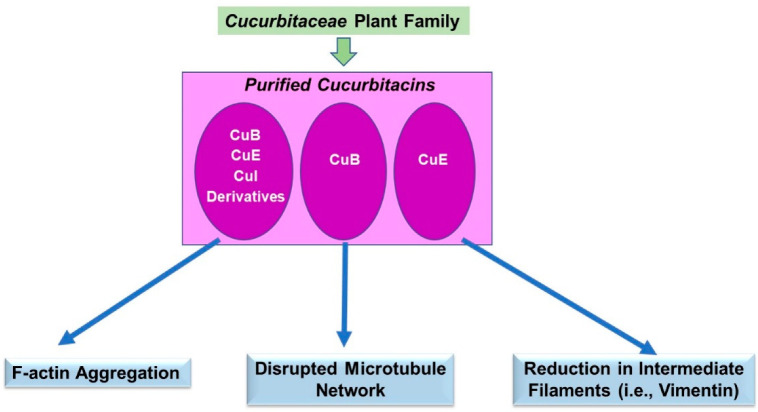
A schematic summary of the effects of metabolites from *Cucurbitaceae* on cytoskeletal alterations.

For the *Cucurbitaceae* family, although it is recognized that species contain multiple phytochemicals, including carotenoids and saponins (amongst other constituents), we focused exclusively on the effect of cucurbitacins (which are highly abundant in various genera of this family) on cytoskeletal alterations. Using our above-described search terms, the research findings that were uncovered primarily revolved around the contribution of cucurbitacin B, E, I, and some derivatives. There were also a few studies identifying key regulatory molecules involved in the cytoskeletal organization (i.e., paxillin, cofilin, Arp2/3, LIMK, and VASP), whose expression and/or activities were modulated by cucurbitacins. The effects of these phytochemicals on actin/microtubule organization and expression of intermediate filaments (i.e., vimentin) were also noted to be altered. The details of these studies are described below in Section 3.2.1, Section 3.2.2, Section 3.2.3 and Section 3.2.4, of which the majority were conducted in cancer cell lines associated with *in vitro* and/or *in vivo* anti-neoplastic responses (i.e., reduction in cellular viability, cell cycle arrest, induction of apoptosis, reduced migration/invasion, reduced adhesion, and diminished tumor growth in murine xenograft models). A schematic of the key findings is displayed in Figure 3.

#### 3.2.1. Cucurbitacin B (CuB)

*Blood Cancers:* CuB-induced alterations in actin filament organization in cells from different blood cancer types within minutes to a few hours following exposure. Specifically, in an acute promyelocytic leukemia cell line (HL60), CuB (0.5 µM) elicited a disorganized accumulation of F-actin into aggregates, as observed via rhodamine-phalloidin immunofluorescence staining [20]. In support, in the Jurkat T lymphocytic cell line, CuB (1 µM) diminished the pool of monomeric G-actin with corresponding increases in F-actin [21]. The changes in actin organization occurred within 30 min and coincided with cofilin dephosphorylation (an actin-interacting molecule that regenerates actin filaments) [21]. Similar cytoskeletal alterations were noted in primary effusion lymphoma (PEL) cell lines (i.e., BCBL-1) in which CuB (30 nM) reduced G-actin levels leading to actin aggregation and decreased p-cofilin levels within 1–6 h of treatment [22]. 

*Solid Tumors:* Alterations in a cytoskeletal organization upon exposure to CuB were also reported in cells derived from solid tumors, including breast, lung, brain, and melanoma. In breast cancer cell lines, CuB-induced alterations in all three major components of the cytoskeleton: actin, microtubules, and intermediate filaments. In the MCF-7 breast cancer cell line, aggregation of F-actin filaments and microtubules occurred upon exposure to CuB (0.5 µM) within 20 min [23]. In an independent report using the same breast cancer cell lines (MDA-MB-231 and MCF-7), CuB (2.5–10 µM) inhibited the assembly of microtubules within 15 min, as noted via immunofluorescence staining of α-tubulin [24]. In MDA-MB-231 and SKBR-3 breast cancer cell lines, CuB (30 nM) altered the cytoskeletal organization with reduced vimentin along with increased F-actin aggregates in the perinuclear area [25]. In one report studying H1299 lung cancer cells, cytoplasmic aggregates of F-actin were observed following 2 h of CuB (0.1–0.35 µM) exposure, which partially required activation of p38 MAPK, as shown through the use of the SB203580 inhibitor [26]. In human T98G and U87 glioblastoma multiforme (GBM) cell lines, within 30 min exposure to CuB (0.1 µM), F-actin formed aggregates, and the microtubule network became disrupted [27]. In human A375 and murine B16F10 melanoma cell lines, CuB (0.1 µM) induced F-actin aggregation within 30 min [28], an event that was dependent on VASP (barbed-end F-actin binding protein) clustering and co-localization. CuB also induced VASP phosphorylation, mediated by Protein Kinase A (PKA) in a cAMP-independent manner [28]. Furthermore, Gα13 and RhoA were shown to contribute to PKA activation, as demonstrated through siRNA targeting these upstream molecules [28].

#### 3.2.2. Cucurbitacin E (CuE)

*Blood Cancers:* Reports of CuE-induced alterations in cytoskeletal organization in blood cancer cells is minimal, with only one report in a leukemia cell line. In human U937 leukemia cells, CuE (1–100 nM, 24 h) reduced p-cofilin levels in the absence of a change in p-LIMK1/2, which is the kinase involved in regulating cofilin phosphorylation [29]. Interestingly, using biotin-linked CuE in an affinity binding assay in these cells, a 20kDa cofilin protein was identified as an interacting partner [29]. Further studies involving molecular docking simulation may be needed to determine whether this is a direct and high-affinity interaction.

*Solid Tumors:* Alterations in a cytoskeletal organization upon exposure to CuE are comparatively more extensive in cells derived from solid tumors, including prostate, breast, lung, and intestinal epithelial cells. In the prostatic PC-3 cell line, CuE (50 nM, 24 h) altered the F-actin intracellular arrangement to an aggregated network in the cytoplasm with a loss of G-actin [30]. CuE treatment, however, did not disrupt the microtubular network (using staining of β-tubulin) [30]. Although the intermediate filament cytokeratin was not altered, CuE disrupted the vimentin distribution to cell-surface membrane blebs [30]. In the MDA-MB-231 breast cancer cell line, CuE (0.2 µM, 1–6 h) reduced focal adhesions (i.e., through paxillin staining) and polymerized actin (i.e., through phalloidin staining) [31]. Using an *in vitro* assay, CuE inhibited the activity of Arp2/3, a critical mediator of lamellipodia formation [31], and reduced its protein levels in cells [31]; furthermore, when Arp2/3 was overexpressed in the breast cancer cells, the cellular response to CuE was diminished in terms of modulating the actin network [31]. In lung 95D cancer cells, CuE (50–1000 nM, 24 h) induced aggregation of F-actin with no marked alteration on the intracellular distribution of β-tubulin [32]. In human Caco-2 intestinal epithelial cells, CuE (0.1 µM, 6–24 h) induced the aggregation of F-actin filaments as well as reduced p-cofilin and p-LIMK protein expression [33].

*Other:* In an *in vitro* study, CuE (0.5 µM) inhibited rabbit skeletal muscle actin depolymerization by binding to F-actin through a covalent bond at Cys257, simulating the behavior of a small molecule modulator [34].

#### 3.2.3. Cucurbitacin I (CuI)

*Solid Tumors:* Alterations in the cytoskeletal organization were reported with CuI in cells derived from cervical cancers. Using the cervical HeLa cell line, CuI (10–100 nM, 2 h) caused aggregation of actin in a disulfide bridge-independent manner (at C272, C374, and C257) [35]. Furthermore, CuI reduced p-cofilin levels by interacting directly with LIMK1 [35]; through molecular docking simulations and taking advantage of the atomic structure of LIMK1-staurosporine complex, CuI was identified to dock into the nucleotide pocket of LIMK1 [35].

#### 3.2.4. Other Cucurbitacins

*Solid Tumors:* Cucurbitacin derivatives have also been reported to alter cytoskeletal networks. In the prostatic CWR22Rv-1 cell line, cucurbitacin IIa (CuIIa, 50 µg/mL, 48 h) disrupted the actin cytoskeleton by causing aggregation of F-actin filaments (i.e., staining with rhodamine-phalloidin) with no alteration in microtubule network (i.e., staining with α-tubulin) [36]. In prostate cancer cells, 23,24-dihydroCuF (DHCF) decreased p-cofilin (20 µM, 24 h) without effect on microtubule cytoskeleton but decreased G-actin while increasing actin aggregation (20 µM, 24 h) [37]. In the human fibrosarcoma HT1080 cell line, isocucurbitacin D (IsoCuD, 1–1000 nM) reduced p-cofilin levels while increasing p-LIMK1/2 levels [38]. IsoCuD also resulted in a reduced F-actin/G-actin ratio elucidated by examining the polymerization rate for 24 h [38].

### 3.3. Ericaceae Phytochemicals on the Cytoskeleton

**Figure 4:**
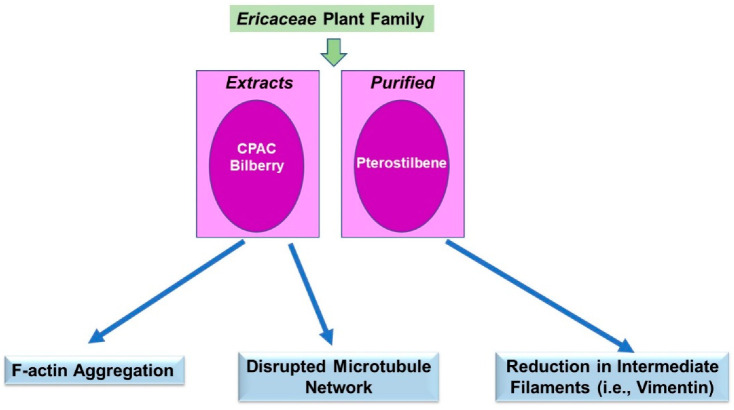
A schematic summary of the effects of metabolites from *Ericaceae* on cytoskeletal alterations.

For the *Ericaceae* family, using our above-described search terms, the research findings that were uncovered utilized extracts as well as purified phytochemicals. Amongst these studies, the majority were descriptive of the visual alteration in the cytoskeletal network and thus limited with respect to the underlying mechanism of action. These are described below in Section 3.3.1 and Section 3.3.2, primarily conducted in cancer cell lines associated with *in vitro* and *in vivo* anti-neoplastic responses as described for *Cucurbitaceae*. A schematic of the key findings is displayed in Figure 4.

#### 3.3.1. Extracts

*Solid Tumors:* Using the cervical HeLa cancer cell line, cranberry proanthocyanidin extract (CPAC, from *Vaccinium macrocarpon*, up to 100 µg/mL, 4 h) disrupted actin polymerization along with delocalization of VASP to focal adhesions and α-actinin/paxillin [39]. CPAC also altered the phagocytic response in a J774 murine macrophage cell line, which was independent of any alteration in host cellular viability [39]. In another study, using an extract derived from a capsule-form of bilberry, deterioration of the actin filament network (F-actin aggregation in cytoplasm, 0.25 mg/mL, 24 h) with changes in the microtubule network (aggregation at specific areas in the cytoplasm, 0.5 mg/mL, 24 h) were noted in MCF-7 breast cancer cells overexpressing GFP-tubulin [40].

#### 3.3.2. Purified Components

*Solid Tumors:* Pterostilbene (trans-3,5-dimethoxy-4-hydroxystilbene, a phytochemical component of berries, 2.5–10 µM) was tested in MCF-7 and MDA-MB-231 breast cancer cell lines and found to inhibit the production of cancer stem-like cells (CSCs characterized by CD44+/CD24- expression) through an NFκB pathway [41]. Specifically, this agent inhibited tumor-associated macrophage (i.e., M2-polarized THP-1) induced CSCs from enriching along with reduced migratory and invasive potential [41]. These changes were accompanied by reduced NFkB expression as well as mediators involved in epithelial-mesenchymal transition (EMT); one such mediator is vimentin, which was reduced (coinciding with increased E-cadherin) [41]. These effects were supported in an *in vivo* mouse model xenograft model with pterostilbene (5 mg/kg) with reduced NFκB and vimentin protein [41].

### 3.4. Rosaceae Phytochemicals on the Cytoskeleton

**Figure 5:**
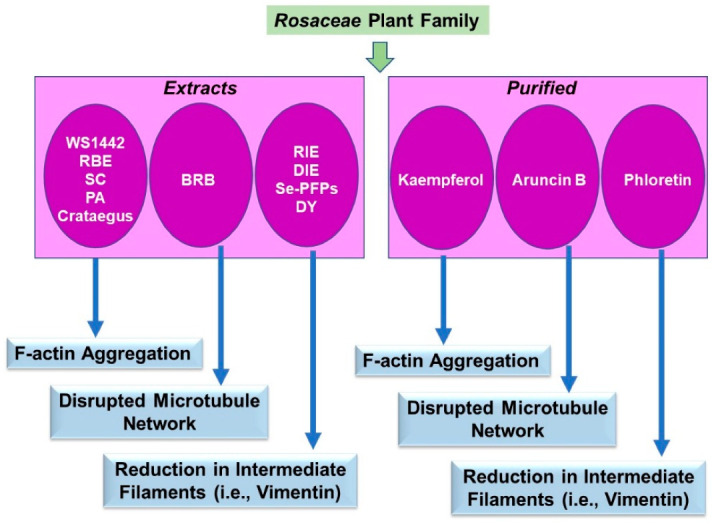
A schematic summary of the effects of metabolites from *Rosaceae* on cytoskeletal alterations.

Similar to the *Ericaceae* family, the research findings that were uncovered for the *Rosaceae* family utilized both extracts and purified phytochemicals. Amongst these studies, the majority were descriptive of the visual alteration in the cytoskeletal network and thus limited to the underlying mechanism of action. These are described below in Section 3.4.1 and Section 3.4.2, conducted across a diverse range of cell lines and some conditions outside the realm of tumorigenesis. A schematic of the key findings is displayed in Figure 5.

#### 3.4.1. Extracts

*Endothelial Permeability and Neovascularization:* WS1442, an herbal extract derived from *Crataegus spp* hawthorn (100 µg/mL), hindered thrombin-induced adherens junction dysfunction and stress fiber formation (i.e., F-actin stress fibers and the contractile network involving myosin) in human umbilical vein endothelial cells (HUVEC) [42]. These alterations were accompanied by reduced intracellular calcium levels, PKC/RhoA inactivation, as well as elevated cAMP and PKA activation leading to VASP phosphorylation [42]. In another study, a red raspberry phenolic extract (50 µg/mL) also induced changes in F-actin, leading to its disorganization in human microvascular endothelial cells (HMVECs) [43]. 

*Hepatic Fibrosis*: A red raspberry extract (RBE, 250 µg/mL) mediated protection against fibrotic response in hepatic stellate cells (HSCs); interestingly, a proteomic-biological network analysis identified changes in pathways involved in cell adhesion and cytoskeletal remodeling [44]. Further, in an *in vivo* model using dimethylnitrosamine- (DMN) treated rats with RBE (25–50 mg/kg), diminished levels of alpha-smooth muscle actin, an indicator of liver fibrogenesis, were noted [44]. 

*Solid Tumors:* Reports of *Rosaceae* extract-induced alterations in cytoskeletal organization encompass multiple tumor types, including liver, brain, prostate, lung, ovarian, and colorectal. In the human HepG3 hepatoma cell lines, stem and cortex extracts from *Sorbus commixta* Hedl (SC, 250 µg/mL, 24 h) inhibited actin filament organization [45]. Likewise, extract preparations from *Crataegus* berries, leaves, and flowers from 6 species reduced cell polarity, coinciding with increased actin aggregates and actin stress fibers in the highly malignant U87MG human glioblastoma cell line [46]. In prostate stromal cancer (PSC) cells, an extract from *Pygeum africanum* (PA, 7.35 µg/mL, 24 h) reduced levels of vimentin and alpha-smooth muscle actin proteins [47]. A raspberry extract from *Rubus idaeus* L (RIE, 100 µg/mL) reduced p-FAK, p-paxillin, vimentin, and N-cadherin protein levels in A549 lung cancer cells; furthermore, using an *in vivo* mouse xenograft model, RIE (500 mg/kg) not only reduced tumor volume but also diminished vimentin protein expression, as noted via immunohistochemical analyses [48]. Leaf extracts from *Duchesnea indica* (DIE, 100 µg/mL, 24 h) also increased E-cadherin protein while decreasing N-cadherin, vimentin, as well as p-paxillin levels in A549 cells [49]. Selenium-enriched polysaccharides from *Pyracantha fortuneana* (Se-PFPs, 200–400 µg/mL, 48 h) increased E-cadherin while reducing N-cadherin and vimentin protein expression in the ovarian cancer HEY and SKOV3 cell lines [50]. Along with reduced tumor volume, diminished expression of vimentin and E-cadherin were also noted in an *in vivo* xenograft model using HEY cells following treatment with Se-PFPs (400 mg/kg) [50]. In colorectal RKO and HCT15 cancer cell lines, extracts from the roots from *Sanguisorba officinalis* L (DY, 100 µg/mL, 24 h) increased E-cadherin levels while reducing N-cadherin and vimentin protein levels [51]. 

#### 3.4.2. Purified Components

*Blood Cancers:* Aruncin B (a monoterpenoid, 30 µg/mL, 36 h) derived from a methylene chloride extract from the aerial parts of the goats’ beard, *Aruncus dioicus* var. *kamtschaticus*, was observed (via α-tubulin staining) to induce damage on the microtubule network in Jurkat T cells [52]. 

*Solid Tumors:* In an *in vivo* xenograft model using breast cancer cells, phloretin (Ph, 100–150 mg/kg), a polyphenol from apple, reduced tumor weight as well as N-cadherin and vimentin expression [53]. Moreover, Ph (10–150 µM, 24 h) reduced mediators involved in the cytoskeletal organization, including p-FAK, paxillin, and alpha-smooth muscle actin in the breast cancer MDA-MB-231 cell line [53]. In addition to a black raspberry extract (BRB), some purified components, namely ellagic acid (EA), urolithin A (UA), and protocatechuic acid (PCA), were tested in combination with docetaxel-and cabazitaxel in castration-resistant prostatic cancer (CRPC) cell lines [54]. Differing effects were noted between the extract (1 mg/mL) and the purified components; specifically, EA (as well as PCA and UA, 10–30 µM) increased microtubule assembly, whereas BRB extract inhibited assembly [54]. Together with cabazitaxel (a chemotherapeutic agent), all the compounds and the extract decreased tubulin polymerization [54]. The roots of *Sanguisorba officinalis* L have been used traditionally for loss of pigment; in this context, kaempferol (15 µM) markedly increased melanin content while also increasing dendrite length and cytoskeletal F-actin filaments in the malignant SK-MEL-28 melanoma cell line [55]. Kaempferol also elevated RhoA and CDC42 expression, which are critical in mediating actin-dependent melanosome transport [55].

## 4. Phytochemical-Induced Alterations on Protein Trafficking

### 4.1. Importance of Protein Trafficking Dynamics

In order to support a variety of cellular functional outcomes (i.e., growth, migration, invasion, etc.), numerous molecules must traffic from various originating locations to a specific destination to mediate a specific molecular function [56]. This may include movement from the endoplasmic reticulum (ER) along the secretory route to the cell surface or plasma membrane, where they could potentially function as mediators in signal transduction [57]. Along this route, these proteins pass through a series of compartments, including various stacks of the golgi apparatus and transport vesicles, that eventually fuse with the cell surface in an exocytic event. Proteins are also internalized from the cell surface, moving into endosomal compartments, which are then distributed to secondary locations that may include the lysosome [57]. Other molecules may be retrograde transported to the trans-golgi network or directly to the endoplasmic reticulum. Some proteins may be translocated to other organelles, including the nuclear or mitochondrial compartments using unique transport mechanisms. Cytoskeletal involvement, including actin, microtubules, and intermediate filaments, in addition to motor proteins (i.e., dynein and kinesin) and targeting molecules (i.e., SNAREs and Rabs), are all critically involved in protein trafficking events. These need to be critically regulated to support cellular homeostasis and, when dysregulated, can result in cellular abnormalities contributing to the pathogenesis of specific diseases [56]. The endoplasmic reticulum is also involved in quality control, and when this is dysregulated, accumulated misfolded proteins may lead to ER stress response [57]. Drugs that target these events to restore cellular homeostasis would be of clinical benefit in various diseases [56]. We urge the reader to review the literature such as [57] for a broader background in this research field. Herein, we focus on organellar dynamics, trafficking of proteins along the secretory route, as well as translocation of key mediators involved in signaling events, including cytoplasmic to nuclear movements. Please see Table 5 for a summary of plant components investigated with respect to health relevance or associated disease models.

### 4.2. Cucurbitaceae Phytochemicals in Protein Trafficking Dynamics

**Figure 6:**
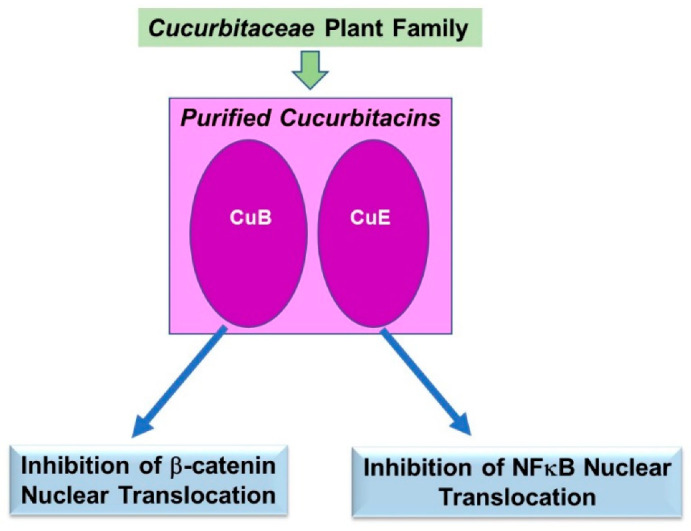
A schematic summary of the effects of metabolites from *Cucurbitaceae* on protein trafficking dynamics.

The literature describes cucurbitacins’ cellular response primarily in altering the nuclear translocation of key signaling molecules such as β-catenin and the p65 subunit of NFκB. Only CuB and CuE were thus far reported to modulate such protein trafficking events. A schematic of the key findings is displayed in Figure 6.

#### 4.2.1. Cucurbitacin B (CuB)

*Solid Tumors:* β-catenin is a critical Wnt signaling mediator that normally translocates from the cytoplasmic compartment to the nucleus; it is frequently deregulated in breast tumors leading to increased gene expression of cyclin D1 and c-Myc. In a series of breast cancer cell lines (MCF-7, SKBR-3, and T47D), CuB (25 µg/mL, 24 h) not only reduced β-catenin protein expression but it hindered its ability to translocate into the nuclear compartment [58].

#### 4.2.2. Cucurbitacin E (CuE)

*Rheumatoid Arthritis:* This disease is characterized by inflammation, damage to bone or cartilage, and deformed joints. In the synoviocyte MH7A cell line stimulated with TNFα, CuE treatment (10 nM) reduced the gene expression of pro-inflammatory cytokines (i.e., IL-1β, IL-6, and IL-8) together with a reduction in p-NFκB levels [59]. The nuclear translocation of p65, a subunit of NFκB, was also markedly reduced upon CuE treatment [59].

### 4.3. Ericaceae Phytochemicals in Protein Trafficking Dynamics

**Figure 7:**
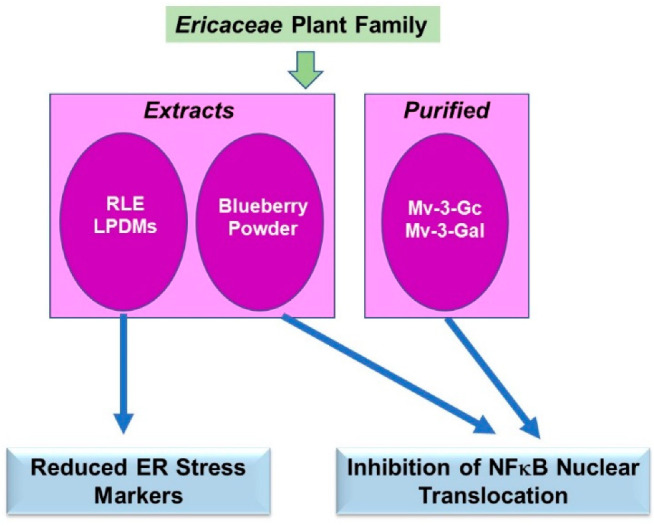
A schematic summary of the effects of metabolites from *Ericaceae* on protein trafficking dynamics.

The phytochemical literature for the *Ericaceae* plant family for protein trafficking primarily focused on the dysregulated nuclear translocation of the p65 subunit of NFκB in addition to descriptive analyses of ER stress markers in cell model systems of cancers, inflammation, and neurodegenerative diseases. A schematic of the key findings is displayed in Figure 7.

#### 4.3.1. Extracts

*Solid Tumors:* Blueberry powder was utilized to supplement a basal diet in a 7,12-dimethylbenz[a]anthracene (DMBA)-induced hamster buccal pouch cancer model [60]. The blueberry supplementation mediated a protective response (100–200 mg/kg) that entailed reduced tumor burden, expression of NFκB, and nuclear translocation of the p65 subunit [60]. In the cervical HeLa cancer cell line, the *Rhododendron luteum* extract (RLE, 40 µg/mL, 72 h) increased the RNA expression of CHOP, an ER stress marker [61]. Since the ER is a critical regulator of apoptotic response, increased CHOP may thus contribute to the cell death response.

*Neurodegenerative Disease:* Neurodegenerative diseases such as Parkinson’s (PD) are characterized by misfolding and aggregation of alpha-synuclein (aSyn) as well as by ER stress and induction of autophagy [62]. The (poly)phenol-digested metabolites from the leaves of *Arbutus unedo* (LPDMs, 2 µg/mL) antagonized aSyn aggregation in the human neuroglioma H4 cell line while decreasing RNA levels of ER stress markers (LPDMs, 62 µg/mL) in yeast cells [62].

#### 4.3.2. Purified Components

*Inflammation:* In the endothelial HUVEC cell line treated with TNFα, combinatorial administration of two major blueberry anthocyanins (malvidin-3-glucoside (Mv-3-Gc) and malvidin-3-galactoside (Mv-3-Gal), up to 100 µM) reduced the pro-inflammatory response (i.e., MCP-1, ICAM-1, and VCAM-1) and the translocation of the p65 subunit of NFκB into the nuclear compartment [63].

### 4.4. Rosaceae Phytochemicals in Protein Trafficking Dynamics

**Figure 8:**
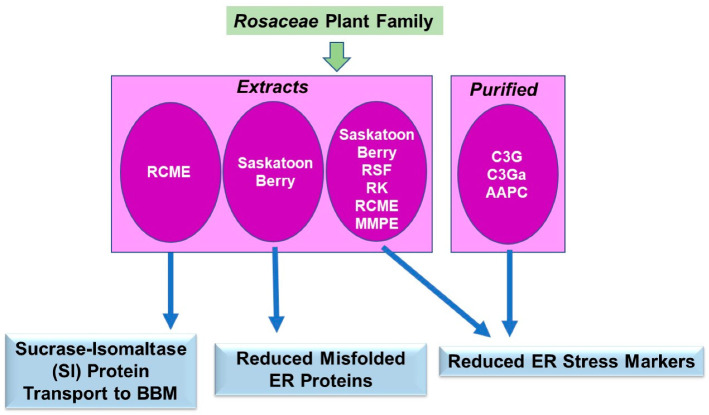
A schematic summary of the effects of metabolites from *Rosaceae* on protein trafficking dynamics.

The phytochemical literature for the *Rosaceae* plant family in protein trafficking primarily focused on expression patterns of ER stress markers in multiple *in vitro* and/or *in vivo* model systems of obesity, cancer, inflammation, heart and liver diseases, as well as heavy metal toxicity. Interestingly, one study featured the trafficking of a key enzyme from the ER to the cell surface in cells relevant to intestinal bowel disease (IBD). A schematic of the key findings is displayed in Figure 8.

#### 4.4.1. Extracts

*Obesity:* Raspberry seed powder (RSF) supplementation was investigated on metabolic outcomes of a high-sucrose diet in mice [64]. In this *in vivo* murine model, RSF recovered glucose metabolism and reduced triglycerides to normal levels, but it also reduced liver ER stress (assessed via western analyses of the stress markers p-JNK, p-p38, and p-eIF2α) [64]. In an ovariectomy-induced obese rat model, raspberry ketone (RK, 160 mg/kg) reduced body weight and protein expression of ER stress markers (i.e., reduced BIP and IRE1α) [65]. In a high-fat, high-sucrose (HFHS) fed mouse model, the Saskatoon berry powder (SBp, 5%) and one of its major components cyanidin-3-glucoside (C3B, 5%) reduced glucose, triacylglycerides, as well as ER stress markers (i.e., CHOP) [66].

*Solid Tumors:* Increased levels of ER stress markers (i.e., ATF-6 and XBP-1) were reported alongside a series of apoptotic markers in colorectal HCT-116 cancer cells that were treated with strawberry tree honey from *Arubutus unedo* L. (STH), which was more potent in combination with 5-fluorouracil (5-FU) [67].

*Inflammation:* One potential key therapeutic target in intestinal bowel diseases (IBD) is intestinal sucrase-isomaltase (SI), responsible for digesting disaccharides [68]. Reduced levels of SI at brush border membrane (BBM) lipid rafts may give rise to symptoms such as diarrhea and abdominal pain [68]. In this context, the response of a methanol extract from *Rosa canina* (RCME) was investigated on dextran sodium sulfate- (DSS) induced dysregulation of ER homeostasis and protein trafficking using Caco-2 intestinal cancer cells [68]. RCME (50–1000 µg/mL) reduced the expression of ER sensors (including CHOP, ATG4, BiP, GRP94, and XBP1) and restored the trafficking of SI to cell surface lipid rafts [68].

*Heavy Metal Toxicity:* Lead exposure typically targets the liver and kidney, leading to oxidative stress within these tissues and possibly ER stress [69]. A polyphenol extract from the pulp of *Malus micromalus Makino* (MMPE, 100 mg/kg) was tested in lead-treated mice [69]. Along with recovered body weight, reduced erythrocyte lysis, and improved liver and kidney functions, MMPE reduced calreticulin (CRT) protein expression, which is associated with the ER stress pathway [69].

#### 4.4.2. Purified Components

*Cardiovascular Disease:* The effect of Saskatoon berry (SB, in which the major anthocyanins are cyanidin-3-galactoside (C3Ga) and cyanidin-3-glucoside (C3G)) was investigated in a murine diabetic model (db/db) [70]. Although SB (5%) did not alter body weight, blood glucose, or serum cholesterol, there were reduced misfolded ER proteins (as measured via the thioflavin T assay (ThT)) [70]. Furthermore, SB reduced ER stress markers (i.e., GRP78/94, XBP-1, and CHOP) within the hearts and aorta of the db/db mice [70]. HUVEC cells stimulated by glycated LDL, C3G, or C3Ga (30 and 100 µM, respectively) also reduced these ER stress markers [70].

*Liver Disease:* In a rat model of high-fat diet-induced non-alcoholic fatty liver disease (NAFLD), a compound present in *Potentilla chinensis*, namely Asiatic acid (AAPC, at 4 and 8 mg/kg), was found to improve liver injury and reduce ER stress markers (i.e., GRP78, PERK, eIF2α, and CHOP) [71].

## 5. Phytochemical-Induced Alterations on Signaling

### 5.1. Importance of Signaling Events

Cytoskeletal alterations and protein trafficking dynamics are intertwined into signaling events. Indeed, these cellular activities are modulated by stimulation of a signaling cascade mediated by receptor activation. The contributors to these signaling pathways are numerous; their complexity is further increased through the large array of intracellular binding partners for each of these mediators [72]. Another level of complexity arises from post-translational modifications on each of these signaling mediators [72]. In sum, these all contribute to the challenges in defining the underlying aberration in a pathological disease. Drugs that target signaling events can include those that (1) elicit structural change, (2) inhibit enzyme activities through binding to catalytic sites, (3) inhibit enzymes by binding to an allosteric site, (4) are agonists or antagonists to ligand-receptor interactions, or (5) deregulate expression of a signaling molecule [72]. In each of our selected plant families, the major pathways that we assessed herein are the MAPK, PI3K/AKT/mTOR, and JAK/STAT pathways. Please see Table 6 for a summary of plant components investigated with respect to health relevance or associated disease models.

### 5.2. Cucurbitaceae Phytochemicals in Signaling

**Figure 9:**
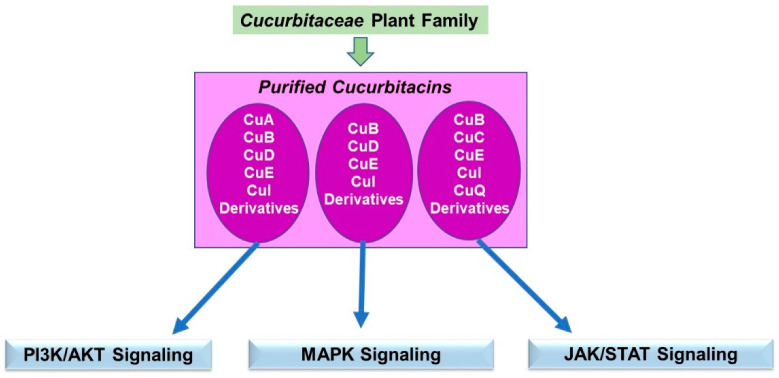
A schematic summary of the effects of metabolites from the *Cucurbitaceae* on the MAPK, PI3K/AKT, and JAK/STAT signal transduction pathways.

A schematic of the key findings is displayed in Figure 9.

#### 5.2.1. Cucurbitacin A (CuA)

##### PI3K/AKT/mTOR Signaling

*Solid Tumors:* In lung A549 adenocarcinoma cells, CuA (200 µM) reduced cell viability and clonogenic potential by inducing apoptosis and G2/M phase arrest [73]. These cellular alterations were accompanied by reduced activation of signaling mediators, including p-AKT, p-mTOR, and p-PI3K, although the total proteins for these latter two were also reduced [73]. In ovarian SKOV3 cancer cells, CuA (300 µM) similarly reduced cellular viability and colony formation ability, possibly by induction of G2/M arrest and apoptosis [74]. Similar to A549 cells, the cellular alterations in SKOV3 cells were accompanied by reduced p-AKT, p-mTOR, and p-PI3K, although the total proteins of the latter two were also reduced [74].

#### 5.2.2. Cucurbitacin B (CuB)

##### PI3K/AKT/mTOR Signaling

*Cardiac Hypertrophy:* In a human cardiomyocyte cell line (AC16) stimulated with phenylephrine (PE), CuB (1 nM) increased apoptosis and antagonized the fibrotic response, which contributes to cardiac hypertrophy [75]. Overexpression of constitutively activated AKT hindered the CuB-mediated reduction in fibrosis [75].

*Solid Tumors:* In a neuroblastoma cell line (SHSY5Y), CuB (5 µM)-mediated reduction in cellular viability and the proliferative index was accompanied by a reduction in p-AKT, which coincided with increased PTEN [76]. Knockdown of PTEN resulted in the recovery of cell survival and proliferation upon CuB treatment, implicating the involvement of PTEN in CuB-induced cellular responses [76]. In a series of human astrocytoma cell lines, CuB (IC_50_ of 0.93–0.49 µM) inhibited cell viability, clonogenic potential, invasion, and migration with an increased apoptotic response [77]. These functional outcomes were also accompanied by reduced p-AKT protein [77]. Similarly, in breast cancer cell lines, CuB (75 nM) inhibited cell survival and induced apoptosis. These cellular outcomes were accompanied by reduced cell surface tyrosine kinase receptor expression (i.e., HER2/neu and EGFR), suppression of integrins (another cell surface receptor), and reduction in p-AKT as well as p-ERK [78]. However, in an independent study, CuB (1.6 µM) reduced cellular viability and increased apoptosis, but reductions in p-AKT were not aligned with alterations in MAPK signaling [79]. In BEL-7402 hepatocellular carcinoma cells, CuB (100 nM) promoted apoptosis with increased DNA damage response [80]. Together with these alterations, CuB reduced p-mTOR, p-AKT, and p62 with increased levels of p-ULK1 and LC3B-II (which are autophagy markers) as well as elevated p-PTEN [80]. In the KKU-100 cholangiocarcinoma cell line, CuB reduced cell viability and increased apoptotic response (20 µM) [81]. These functional changes were accompanied by changes in the expression of p-PI3K and p-AKT [81]. In a series of colorectal cell lines, CuB (0.742 µM) hindered the interaction between laminin and integrin, which reduces tumor budding [82]. Via microarray profiling, it was demonstrated that gene expression of mediators within the PI3K/AKT and focal adhesion signaling events were altered by CuB [82].

##### JAK/STAT Signaling

*Solid Tumors:* In a series of pancreatic cancer cell lines, CuB (ED_50_ of 10^−7^M) reduced cell viability and clonogenic potential while inducing G2/M phase arrest and apoptosis. These alterations were accompanied by reduced p-STAT3, p-STAT5, p-JAK2, and increased p-MAPK [83]. Changes in JAK-STAT signaling (i.e., reduced p-STAT3) were also noted in an independent study using Panc-1 pancreatic cancer cell line, though at higher doses of CuB (up to 3 µM) that coincided with reduced cell viability, G2/M phase arrest, and increased apoptosis [84]. In yet another Panc-1 study in which CuB (at 0.5–1.0 mg/kg) reduced tumor volume when cells in Matrigel were grafted into nude mice, alterations in JAK-STAT signaling (i.e., reduced levels of p-STAT3 and p-JAK2) were also noted [85]. In the human U2OS osteosarcoma cell line, CuB reduced cell viability (100 µM) and migration with increased apoptosis [86]. These alterations were accompanied by reduced p-JNK, p-ERK, p-p38, p-JAK2, and p-STAT3; however, all of their total proteins were also reduced [86]. In human HCT116 colorectal cancer cells, CuB reduced clonogenic potential and cellular viability (800 nM) along with an apoptotic response [87]. These cellular outcomes were associated with reduced p-JAK2 and p-STAT3 proteins [87]. In the lung A549 adenocarcinoma cell line, CuB hindered cell viability (0.9 µM), increased apoptotic response, and reduced clonogenic potential [88]. These changes were also associated with reduced p-STAT3 levels [88].

##### MAPK Signaling

*Solid Tumors:* In hepatic-derived cancer cell lines, CuB (1 µM) hindered 12-O-tetradecanoylphorbol 13-acetate (TPA) mediated migration, invasion, and epithelial-mesenchymal transition (EMT) along with reduced cellular viability and clonogenic growth [89]. These cellular outcomes were accompanied by reduced activation of MAPK (i.e., p-ERK, p-p38, p-JNK) in addition to p-AKT proteins [89]. In a series of gefitinib resistant non-small cell lung cancer cell lines, CuB (0.2 µM) not only reduced clonogenic potential, invasion, and migration [90], but the expression in p-ERK, p-AKT, and EGFR reduced markedly [90]. In a series of melanoma cell lines, CuB increased apoptotic response with reduced p-MEK1/2, p-MAPK, and p-STAT3 [91].

#### 5.2.3. Cucurbitacin C (CuC)

##### JAK/STAT Signaling

*Solid Tumors:* Across an array of cancer cell lines, CuC reduced their viability (IC_50_ of 10–100 nM) along with reduced clonogenicity, migration, G2/M or G1 phase arrest, and elevated apoptotic response [92]. These events were accompanied by reduced p-AKT, although the GO/KEGG pathway analyses also identified the JAK/STAT pathway as the most altered in the hepatoma HepG2 cell line [92].

#### 5.2.4. Cucurbitacin D (CuD)

##### PI3K/AKT/mTOR Signaling

*Cancers:* In a series of human gastric cancer cell lines, including AGS cells, CuD (2 µM) induced apoptosis with increased reactive oxygen species (ROS) generation; these functional alterations were accompanied by reduced p-AKT and p-mTOR levels [93].

##### MAPK Signaling

*Cancers:* In MT-4 adult T cell leukemia cells, CuD (1 µM) promoted cell death and was associated with reduced p-MAPK, p-p38, and p-JNK [94]. In a series of pancreatic cancer cell lines including Capan-1, CuD (0.8 µM) reduced cellular viability with a G2/M phase arrest, increased apoptotic response and elevated ROS levels, which coincided with increased p-p38 levels, in the absence of change in p-JNK [95].

#### 5.2.5. Cucurbitacin E (CuE)

##### PI3K/AKT/mTOR Signaling

*Solid Tumors:* In human esophageal carcinoma cell lines, CuE (10 µM) reduced cellular viability, migration, and invasion, which were accompanied by reduced p-AKT and p-mTOR levels [96]. In Caco-2 human intestinal cancer cells, CuE (1 µM) induced apoptosis; this cellular outcome was accompanied not only by increased ER stress markers (i.e., CHOP and GRP78) and autophagy markers (i.e., LC3B-II and Beclin-1), but reduced p-mTOR and p-AKT (along with reducing total AKT) proteins were also reported [97].

##### JAK/STAT Signaling

*Solid Tumors:* In human Huh7 hepatoma cells, CuE (40 nM) inhibited migration together with G2/M phase arrest [98]. Gene expression profiling identified alterations in several signaling pathways, including the JAK/STAT pathways, amongst others (i.e., actin cytoskeleton, angiogenesis, focal adhesion) [98]. In addition to F-actin aggregation in response to CuE, western blotting validated reduced p-JAK3 and p-STAT3 protein expression levels [98].

*Neovascularization:* CuE (1 nM) not only inhibited tubulogenesis in HUVEC cells, but VEGF-treated HUVEC cells diminished p-VEGFR2 levels along with reduced p-JAK2, p-STAT3, p-ERK, and p-p38 [99]. Furthermore, CuE hindered the nuclear movement of STAT3 from the cytoplasm [99].

##### MAPK Signaling

*Solid Tumors:* In A549 cells, CuE (2.5 µM) induced apoptosis along with reduced p-STAT3 and p-MEK1/2 levels, whereas p-EGFR and p-ERK were elevated [100]. Interestingly, molecular docking simulation of the CuE-EGFR complex identified CuE interaction with the ATP binding site of the EGFR kinase domain; this interaction was stabilized by H-bonds with Leu694, Met769, Arg817, and Asp831 [100]. Across a series of cancer cell lines, including MDA-MB-468, CuE (100–200 nM) induced G2/M phase arrest and apoptosis; these cellular outcomes were associated with reduced p-STAT3, p-AKT, and p-ERK with increased levels of p-JNK [101].

#### 5.2.6. Cucurbitacin I (CuI)

##### PI3K/AKT/mTOR Signaling

*Solid Tumors:* In A549 lung cancer cells, CuI (200 nM) reduced cellular viability and induced apoptosis [102]. These changes were accompanied by reduced PI3K, p-AKT, and p-p70S6K levels [102]. Furthermore, the inhibition of PI3K contributed to the detrimental effect of CuI on A549 cell health [102].

##### JAK/STAT Signaling

*Blood Cancers:* Along with CuI-mediated apoptotic response in CD4+T cells from patients with Sezary syndrome, an aggressive type of lymphoma, CuI (30 µM) also induced a reduction in p-STAT3 (as well as total STAT3 levels) [103].

*Solid Tumors:* CuI (10 µM) treatment in NIH 3T3 cells led to a reduction in p-STAT3 and p-JAK2, which was confirmed in an array of tumor cell lines [104]. CuI was also shown to mediate this inhibition by disrupting the DNA binding activity of STAT3 and, henceforth, its subsequent effect on gene expression [104]. In an independent study, inhibition of CuI on p-STAT3 was confirmed across an array of cancer cells [105]. In cancer-associated fibroblasts (CAFs), CuI (50 nM) promoted apoptosis with inhibition of p-STAT3 [106]. In human malignant glioma cell lines, CuI (up to 400 nM) reduced cellular viability with an apoptotic response and induction of G2/M phase arrest [107]. These cellular outcomes were accompanied by reduced p-STAT3 [107]. CuI (IC_50_ of 170–245 nM) response in glioblastoma cell lines resulted in increased p-AMPK with decreased p-p70S6K, p-mTOR, p-JAK, and p-STAT3 [108]. These changes coincided with cell death accompanied by the increased autophagic response (i.e., LC3B-II punctae and autophagosomes identified via transmission electron microscopy) [108]. In DU145 prostate cancer cells treated with EGF or IL-6, CuI (50 nM) was noted to reduce STAT3 activity and STAT3 nuclear localization [109]. In nasopharyngeal cell lines, CuI (1 µM) reduced cellular viability, and clonogenicity, while simultaneously inducing apoptosis [110]. These cellular outcomes were associated with reduced p-STAT3 [110]. In MDA-MB-468 breast cancer cells, CuI (1 µM) reduced p-STAT3 levels, which accompanied reduction in cellular viability, adhesion, migration, and tube formation [111].

##### MAPK Signaling

*Blood Cancers:* CuI (1 µM, non-toxic doses) was found to induce p-JNK in the BJAB Burkitt lymphoma cell line and the pre-acute NALM-6 lymphocytic leukemia cell line; CuI treatment, however, increased VEGF levels [112].

*Solid Tumors:* In colon cancer cell lines, CuI (10 nM–1 µM) was most effective in reducing cellular viability and inducing apoptosis in cells lacking a K-RAS activating mutation [113]. In A549 cells, CuI (400 nM) reduced cellular viability and clonogenicity with increased apoptosis [114]. These changes were accompanied by elevated LC3B-II (autophagy marker) with reduced AKT, PI3K, p-PI3K, p-mTOR, p-ERK, and p-STAT3 [114]. When autophagy was inhibited using an inhibitor (3-methyladenine, 3-MA), the effect of CuI on ERK/mTOR/STAT3 signaling was reversed, suggesting that autophagic induction is responsible for the CuI-mediated alterations through this signaling pathway [114]. In gastric cancer cell lines, CuI reduced cellular viability (IC_50_ of 97.4 to 123 nM) with G2/M phase arrest, induction of apoptosis, and DNA damage [115]. These changes, however, were not accompanied by changes in p-STAT3 but only elevated p-p38 and p-JNK proteins [115]. Furthermore, pretreatment with a JNK inhibitor (SP600125) antagonized the effect of CuI on cellular viability and apoptosis, implicating the JNK pathway in mediating these observed cellular responses of CuI [115].

*Cardiac Tissue:* In cardiac H92c cells derived from embryonic rat heart tissue with characteristics of cardiomyocytes, CuI (0.1–0.3 µM) increased apoptosis, which coincided with autophagy alterations (i.e., increased LC3B-II) [116]. These events were also associated with elevated p-ERK1/2, p-JNK, and p-p38 [116]. Moreover, pathway inhibitors such as U0126 (MAPK) inhibited the autophagic induction and apoptotic response induced by CuI [116], suggesting that CuI mediates these functional responses through the MAPK signaling cascade.

#### 5.2.7. Cucurbitacin Q (CuQ)

##### JAK/STAT Signaling

*Solid Tumors:* Although CuQ (0.5–1 mg/kg) inhibited tumor growth most effectively using A549 cells in a mouse xenograft model along with reduced p-STAT3 levels in a series of cancer cell lines, CuQ (10 µM) did not elicit a change in p-JAK2 (with contrasts with responses to CuA, CuB, CuE, and CuI) [117].

#### 5.2.8. Cucurbitacin Derivatives

##### JAK/STAT and AKT Signaling

*Solid Tumors:* An extract from the leaves of *C. colocynthis* (L.) Shrad (containing cucurbitacin-glycosides) was tested in breast cancer cell lines. The cucurbitacin treatment surprisingly increased p-STAT3 levels, which was associated with G2/M phase arrest and apoptosis; however, p-AKT and cell survival response was reduced [118].

##### MAPK Signaling

*Solid Tumors:* In gastric cancer cells, DHCE (23,24-Dihydrocucurbitacin E) reduced cellular viability (IC_50_ of 3.83–7.53 µM), clonogenic potential, and migration/invasion [119]. In addition to these functional outcomes, DHCE modulated several regulatory signaling pathways that were identified via network pharmacology analysis (i.e., adherens junction, F-actin organization, and Ras/Raf/ERK/MMP9 pathways) [119]. Molecular docking was performed to validate the DHCE effect on the ERK pathways, specifically ERK2 protein; this was performed using AutoDockTools, PyMol, and the X-ray crystal structure of ERK2 (from PDB (Protein Data Bank)) [119]. The analyses identified several hydrophobic interactions from ERK2 with DHCE (Lys106, Leu148, and Val31) and H-bonds (Met100, Lys46, Ile23, and Asp159) [119]. In A549 lung cancer cells, CuIIa (60 µM) reduced cellular viability while increasing apoptosis and G2/M phase arrest [120]. Moreover, CuIIa increased p-EGFR as well as reduced p-MEK and p-ERK levels [120]. Interestingly, CuIIa reduced EGFR kinase activity [120]. Molecular dynamic simulations, based on the three-dimensional structure of EGFR with erlotinib, identified that the long side chain of CuII sits into the binding pocket by two H-bonds with Met769 while making additional H-bonds at the active site with Arg817, Thr830, and Asp831 [120]. In A549 cells, CuIIb (80 µM) reduced cellular viability and increased apoptosis along with G2/M phase arrest [121]. These cellular outcomes were accompanied by reduced p-STAT3, p-EGFR, p-MEK1/2, and p-ERK1/2 [121]. With the finding that EGFR kinase activity was reduced, molecular docking was performed; this analysis investigated the interaction between CuIIb and EGFR (using the X-ray crystal structure of the kinase domain of EGFR) [121]. CuIIb was identified to fit into the hydrophobic cleft of the ATP-binding site with multiple hydrophobic interactions arising from Leu694, Phe699, Val702, Ala719, Met742, Leu768, Met769, Phe771, and Leu829 in addition to H-bonds at Leu694, Met769, Arg817, and Asp831 [121].

### 5.3. Ericaceae Phytochemicals in Signaling

**Figure 10:**
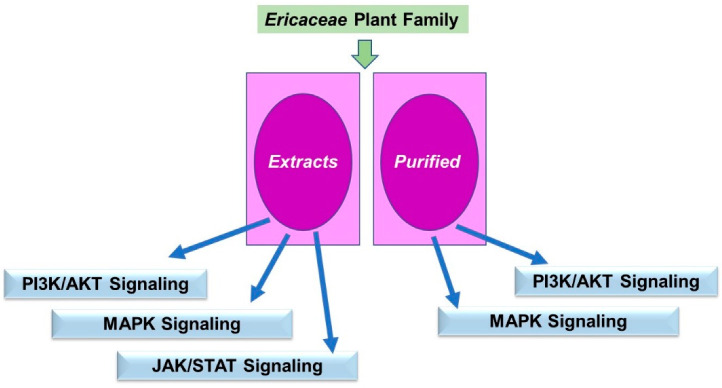
A schematic summary of the effects of metabolites from the *Ericaceae* on the MAPK, PI3K/AKT, and JAK/STAT signal transduction pathways.

A schematic of the key findings is displayed in Figure 10.

#### 5.3.1. Ericaceae Extracts

##### PI3K/AKT/mTOR Signaling

*Blood Cancers:* Antho 50 (25 µg/mL, a bilberry extract from *Vaccinium myrtillus* L.) induced apoptosis in chronic lymphocytic leukemia (CLL) cells; this cellular outcome was associated with reduced pro-survival signaling, including p-AKT and p-BAD levels [122]. Studies have also been performed using whole cranberry extracts from berry juice (CB, 25–50 µg/mL) in the human leukemia HL-60 cell line leading to apoptosis, which was associated with increased dephosphorylation of BAD along with reduced p-AKT [123].

*Solid Tumors:* In the neuroblastoma SMS-KCNR cells, an enriched fraction of cranberry oligomeric proanthocyanidins (PAC-1A, 25 µg/mL) induced a cytotoxic response along with G2/M phase arrest and increased apoptotic response [124]. These cellular outcomes were associated with reduced pro-survival (i.e., p-AKT, p-PI3K, and p-mTOR) and increased pro-death (i.e., increased p-JNK) signaling events [124]. Another cranberry proanthocyanidin extract (PAC-1, 50 µg/mL) was tested on SKOV3 ovarian cancer cells, which reduced cellular viability along with G2/M phase arrest and increased apoptosis; these outcomes were similarly associated with reduced p-AKT levels [125]. In addition, proanthocyanidin-enriched extracts from cranberries (CPAC, 25 µg/mL) reduced cellular viability in DU145 prostate cancer cells that were associated with alterations in signaling pathways, including reduced p-AKT (and total AKT, however) with increased p-p38 and p-ERK1/2 [126]. In an *in vivo* study, whole cranberry powder from *Vaccinium macrocarpon* (WCP, 7.5 g/day) hindered tumor formation in a mouse model of colitis (induced by azoxymethane (AOM) and dextran sulfate sodium (DSS)) [127]. WCP elevated the expression of pro-inflammatory cytokines (i.e., IL-1β, IL-6, and TNF-α) and reduced p-AKT, p-PI3K, and EGFR protein levels [127].

In colorectal HCT-116 cancer cells, a blueberry extract (BE, IC_50_ of 1.26 mg/mL) reduced cellular viability along with an apoptotic response and G0/G1 cell cycle arrest [128]. Associated with these functional changes was reduced expression of pro-inflammatory cytokines (i.e., IL-1β, IL-6, and TNFα) in addition to reduced levels of p-AKT protein [128]. In breast MDA-MB-231 cancer cells, a whole blueberry extract from *Vaccinium angustifolium* (30 µL/mL) hindered hepatocyte growth factor (HGF)-induced migration; this cellular outcome was associated with reduced p-AKT levels [129]. In another study with relevance to chronic use of Snus (smokeless tobacco containing N-nitrosamines), blueberry supplementation (*Vaccinium myrtillus*, 0.5 g/kg) in a rat model (administered snus intragastrically) promoted the health of epithelium in the forestomach, which was associated with reduced expression of p-AKT [130]. Phytochemicals may also offer protection against dysregulated angiogenic events [131]. In this regard, blueberry components (anthocyanins (ACN, 60 µg/mL) and phenolic acids (PA, 60 µg/mL) from whole blueberries) were tested in the HUVEC endothelial cell system [131]. Although ACN reduced tube formation with reduced AKT1 protein in an *in vitro* angiogenesis assay, PA increased angiogenesis with elevated AKT1 protein [131]. These results implicate different cellular outcomes to specific phytochemical fractions within a specific extract.

*Cardiovascular Disease:* One characteristic associated with cardiovascular disease is elevated blood levels of microvesicles (MVs) [132]. The effect of a bilberry extract (BE) was investigated on MVs using patient samples wherein they analyzed baseline and 8 weeks post-treatment levels [132]. The findings show that BE reduced blood-derived MVs, in the absence of BE-mediated toxicity (up to 1000 µg/mL) on HUVECs, and reduced p-AKT with no change in p-p38 expression [132].

*Perimenopause:* Towards a search for alternative treatments to hormone replacement therapy, bilberry anthocyanins (BA, similar in structure to phytoestrogens, at 25, 70, and 140 mg/kg) was examined in a premenopausal rat model [133]. In addition to reduced levels of cholesterol and triglycerides along with improved ovarian morphology and function, elevated levels of GPR30, AKT, and ERK2 mRNA were noted [133], suggesting that activation of AKT signaling may contribute to some health benefits to alleviate perimenopausal symptoms.

*Metabolic Disorders:* An anthocyanin-enriched blueberry extract (BAE, 200 mg/kg) was tested in a high-fat diet (HFD) mouse model and found to reduce body weight, the weight of liver/adipose tissues, and function/histology of liver/adipose tissues [134]. BAE also reduced triacylglycerides and ceramide synthesis, which were associated with reduced PKC-zeta expression, which is proposed to alter AKT signaling [134]. In human retinal capillary endothelial cells (HRCECs), a blueberry anthocyanin extract (BAE, from *Vaccinium ashei*) not only antagonized the reduction in cellular viability induced by high glucose (HG) but also reduced AKT and VEGF protein levels [135].

*Macular Degeneration:* Neovascularization of retinal pigment epithelial cells (RPE), resulting in vision loss in aged individuals, is a clinical feature of macular degeneration (MD) [136]. Blueberry anthocyanin extract, in addition to its major purified components (malvidin (Mv), malvidin-3-glucoside (Mv-3-glc), and malvidin-3-galactoside (Mv-3-gal), at 5 µg/mL) were tested on RPE cells treated with hydrogen peroxide (H_2_O_2_) [136]. Not only were cellular viability and the apoptotic response opposed by these anthocyanin components, but they mediated protection against oxidative stress and decreased VEGF and p-AKT levels while decreasing p-ERK1/2 levels [136].

*Cognitive Functions:* Spatial working memory may also be affected by the consumption of components from blueberries [137]. Compared to aged rats on an unsupplemented diet, aged rats on a diet supplemented with blueberries (BB) performed better in spatial working memory tasks [137]. These changes were correlated with the activation of CREB (cAMP response element binding protein) along with increased BDNF (brain-derived neurotrophic factor) and p-ERK1/2, p-AKT, and p-mTOR in the hippocampal region [137].

##### JAK/STAT Signaling

*Blood Cancers:* Rabbit-eye blueberry leaves (*Vaccinium virgatum* Aiton) was fractionated to generate several extracts to test on adult T-cell leukemia (ATL, 10 µg/mL) cells and an *in vivo* mouse xenograft model (50–100 mg/kg)) [138]. Fractions with the highest concentration of proanthocyanidin (PAC) inhibited cellular viability and reduced tumor volumes while also reducing p-JAK1, p-JAK2, p-JAK3, p-STAT1, p-STAT3, and p-STAT5 (although there was no change in total STATs, total JAKs were decreased) [138].

*Solid Tumors:* In a dimethylbenzanthracene (DMBA)-induced hamster model of oral cancer, blueberry supplementation (200 mg/kg) reduced tumor burden; this outcome was associated with reduced JAK2 and STAT3 RNA expression and reduced p-JAK2 and p-STAT3 protein (along with reducing its nuclear translocation) [139]. Although the blueberry supplement failed to alter cellular viability in SCC131 oral cancer cells, the purified malvidin component induced a marked growth reduction (IC_50_ of 62 µM) associated with reduced p-JAK2 and nuclear p-STAT3 levels [139].

*Inflammation:* In HaCaT keratinocyte cells, a *Rhododendron album* blume methanol extract (RAME, 2.5–20 µg/mL) antagonized TNFα/IFNγ induced alterations in pro-inflammatory cytokines (i.e., IL-8 and IL-6 protein levels) in addition to reducing the activation of MAPKs (i.e., p-JNK, p-ERK, and p-p38), p-JAK1, p-STAT1, and the nuclear translocation of STAT1 [140].

##### MAPK Signaling

*Solid Tumors:* In a series of high-grade glioma cell lines, in which EGFR is frequently amplified and overexpressed, extracts from chokeberry (*Aronia melanocarpa*), elderberry (*Sambucus nigra*), and bilberry (*Vaccinium myrtillus*) were tested [141]. Chokeberry extract was determined to be the most effective in reducing cellular viability (IC_50_ of 30 µg/mL), which was associated with diminished expression of surface-expressed CD44 and EGFR, a cell surface tyrosine kinase receptor that mediates MAPK activation [141]. In HT29 colon cancer cells, a microencapsulated form of bilberry extract reduced cellular viability and p-EGFR protein levels; however, it was not effective in altering EGFR kinase activity, suggesting the release of some bioactive components in this formulation may have been affected [142]. In A431 and a porcine aortic endothelial cell line (overexpressing VEGF receptor, namely VEGFR-2/3), a mixture of 15 anthocyanins ((IC_50_ of 146 µg/mL) from bilberries (i.e., mirtocyan composed of “delphinidin-3-galactoside (16%), -3-glucoside (14%) and -3-arabinoside (12%), cyanidin-3-galactoside (10%), -3-glucoside (11%), and -3-arabinoside (8%), petunidin-3-galactoside (3%), -3-glucoside (8%), and -3-arabinoside (2%), peonidin-3-galactoside (1%), -3-glucoside (4%), and -3-arabinoside (1%), malvidin-3-galactoside (3%), -3-glucoside (5%), and -3-arabinoside (2%)” and “polyphenols”) inhibited VEGFR-2 and EGFR receptor tyrosine kinase activity along with reducing p-EGFR protein expression; these cellular responses occurred only with subtle effects on cellular viability [143]. In breast cancer cell lines, a polyphenol-enriched blueberry preparation (PEBP, from *Vaccinium angustifolium* Ait juice, 200 µM) reduced cellular proliferation without toxicity, invasion/migration, and mammosphere formation [144]. Associated with these cellular outcomes, PEBP reduced p-STAT3, p-AKT, p-PI3K, and p-ERK1/2, as well as increased p-p38, p-JNK, and p-PTEN [144].

Cellular transformation induced by phorbol ester in JB6 P+ mouse epidermal cells was antagonized by lingonberry extracts (1:160 to 1:40 dilution); this cellular outcome was associated with reductions in p-ERK, p-MEK1/2 but no effect on p-JNK or p-p38 signaling molecules [145].

*Inflammation:* Ethanol extracts from stems (VOS), leaves (VOL), and fruits (VOF) from *Vaccinium oldhamii* Miquel were tested on RAW264.7 murine macrophages to evaluate their potential anti-inflammatory activities [146]. Both VOS and VOL reduced viability (100 µg/mL) in contrast to VOF (25–100 µg/mL), which failed to elicit a change [146]. VOS also reduced the expression of pro-inflammatory cytokines (i.e., IL-1β, IL-6, and TNFα) along with suppression of p-ERK1/2, p-p38, and p-JNK [146]. Anthocyanins extracted (BE, 50 mg/kg) from the blueberry Beilu variety (*Vaccinium* sp.) were tested in a mouse model of gastric injury (induced via LPS lavage with pyloric ligation) [147]. BE not only reduced gastric injuries and levels of pro-inflammatory cytokines (IL-6, IL-8, IL-1β, and TNFα), but it also reduced the levels of p-ERK and p-JNK [147]. In liposaccharide (LPS)-stimulated human gastric epithelial cells, BE (150 µg/mL) also reduced pro-inflammatory cytokine production along with reduced levels of p-ERK1/2 and p-JNK [147]. Similarly, in human dermal fibroblasts, an anthocyanin extract from bog blueberry (ATH-Bbe, 10 mg/L) not only antagonized ultraviolet B (UVB)-induced reduction in cellular viability, but it also diminished pro-inflammatory cytokine production (i.e., IL-1β, IL-6, IL-8, and TNF-α) and reduced levels of p-JNK and p-p38 [148]. A high-molecular-weight non-dialyzable material (NDM, containing “0.35% anthocyanins (0.055% cyanidin-3-galactoside, 0.003% cyanidin-3 glucoside, 0.069% cyanidin-3-arabinoside, 0.116% peonidin-3-galactoside, 0.016% peonidin-3-glucoside and 0.086% peonidin-3-arabinoside) and 65.1% proanthocyanidins”) from cranberry juice of *Vaccinium macrocarpon* was tested on a human gingival epithelial line stimulated with IL-1β [149]. Under these conditions, NDM (25 µg/mL) reduced the pro-inflammatory cytokine IL-6; however, there was no marked change in p-ERK1/2 or p-JNK activation [149]. In RAW264.7 macrophages, a mixture of 7 phenolic acids (7PA, composed of “hippuric acid, 3-hydroxyphenylacetic acid, 3-hydroxybenzoic acid, ferulic acid, 3-(3-hydroxyphenyl)propionic acid, 3-(4-hydroxyphenyl)propionic acid, and 3-hydroxycinnamic acid”) was found to reduce expression of pro-inflammatory cytokines (i.e., IL-6 and TNF-αwhile also reducing activation of p-38, p-JNK, and p-ERK1/2 [150].

*Cognitive Function:* Cognitive deficits associated with aging may be due to elevated oxidative and inflammatory (OX/INF) signaling [151]. An assessment of whole extract (Tifblue BB, from *Vaccinium virgatum*) and fractions prepared from wild blueberry juice (from *Vaccinium angustifolium* Aiton, PRE-C18) including anthocyanins (ANTH), high molecular weight proanthocyanidins (HMW), and low molecular weight anthocyanidins (LMW) were tested in rat hippocampal neuronal cells exposed to dopamine (DA), amyloid beta (Aβ_42_), and lipopolysaccharides (LPS) [151]. Altogether, the whole BB extract (500 µg/mL) and combined LMW and HMW (PAC, 15 µg/mL) fractions were most protective against the above-described stressors, with respect to antagonizing detrimental effects on cellular viability along with hindering ROS production and stress signaling of p-38 MAPK [151]. With respect to Alzheimer’s disease (AD), blueberry supplementation (BB, 25–100 µg/mL) fed to mutant amyloid precursor protein/presenillin 1 mice were normal in terms of their Y-maze performance at 12 months of age along with normal amyloid beta burden; furthermore, in primary microglial cells stimulated with LPS, BB (5 µM) reduced pro-inflammatory cytokines (i.e., TNFα and IL-6) and reduced p-p42/44 MAPK proteins [152].

#### 5.3.2. Ericaceae Purified Phytochemicals

##### PI3K/AKT/mTOR Signaling

*Solid Tumors:* Estrogen receptors (ER) may contribute to the pathogenesis of colorectal carcinoma (CRC) with support of ER pathway targeting by pterostilbene (Pter, a component in blueberries and analog of resveratrol); via molecular docking simulations, Pter was identified to dock into the ER-β active site with high affinity, similar to 17-β-estradiol [153]. Using Caco-2 and HCT-116 colorectal cancer cells, Pter (IC_50_ from 2.44 to 1.07 µM) not only reduced cellular viability mediated by 5-fluorouracil (5-FU) along with reducing ER-β levels (9%), but it also reduced p-AKT and p-ERK levels [153]. In human myeloma cell lines, Pter (IC_50_ of 24–22.8 µM) not only reduced cellular viability, but it also increased apoptosis; these cellular outcomes were associated with reduced PI3K and p-AKT along with increased p-p38 but no change in p-JNK [154]. In A549 and H460 non-small cell lung cancer cell lines, Cinnamtannin D1 (CNT D1, A-type procyanidin trimer, a component isolated from *Rhododendron formosanum* leaf extracts, 50–200 µM) reduced cellular viability along with G1 phase arrest; while there was an absence of apoptosis, autophagic induction was identified through vacuole formation and an LC3B-II punctate pattern [155]. Together with these cellular features, CNT D1 also reduced p-mTOR and p-AKT levels while also increasing p-ERK1/2 levels [155].

*Inflammation:* The cellular effect of rhodomeroterpene (RMT, 30 mg/kg, a meroterpenoid from *Rhododendron*) was tested in a murine ischemia-reperfusion (I/R)-induced AKI mouse model and found to improve the inflammatory response and kidney health as assessed via kidney injury markers and histological analyses [156]. In support of these *in vivo* findings, when macrophages were co-treated with LPS/IFNγ, RMT (40 µM) also reduced the expression of pro-inflammatory mediators (i.e., IL-1β, IL-6, and TNFα) as well as p-PI3K and p-AKT [156]. In HUVEC endothelial cells, hyperoside (purified component from the leaves of *Rhododendron brachycarpum*) protected against the pro-inflammatory response by diminishing high mobility group box 1 (HMGB1)-induced activation of p-AKT and p-ERK1/2 [157].

*Metabolic Disorders:* Hypotensive effects may be mediated by the anthocyanin component in the berries (such as *Vaccinium ashei*), of which malvidin (Mv) comprises the major component (i.e., malvidin-3-glucoside (Mv-3-Glc) and malvidin-3-galactoside (Mv-3-Gal)) [158]. High-glucose (HG) stimulated HUVECs were treated with blueberry anthocyanin extract (BAE) and these purified malvidin components [158]. These agents (5 µg/mL) mediated protection against the reduction in cellular viability in addition to restoring the levels of p-AKT and PI3K back to baseline levels [158]. In another study using HepG2 cells overexpressing tyrosine phosphatase 1B (PTP1B, which undergoes deregulated activation in insulin resistance), purified components from anthocyanins (i.e., cyanidin, pelargonidin-3-glucoside, cyanidin-3-arabinoside (Cya-3-Ara), delphinidin-3-glucoside (Del-3-Glu), cyanidin-3-galactoside (Cya-3-Gal), cyanidin-3-glucoside (Cya-3-Glu), malvidin-3-galactoside (Mal-3-Glu), and petunidin- 3-glucoside (Pet-3-Glu)) were tested towards their protective mechanism of action [159]. Out of all these compounds, Cya-3-Ara (10–40 µM) was the most effective in inhibiting PTP1B while improving alterations in the IRS1/PI3K/AKT pathway [159]. Interestingly, through molecular docking simulations, Cya-3-Ara was shown to interact with PTP1B through Tyr46, Val49, Asp181, Phe182, Cys215, Ala217, and Arg221 [159].

##### MAPK Signaling

*Solid Tumors:* In SKOV3 and OVCAR-8 ovarian cancer cell lines, purified cranberry flavonols (i.e., “myricetin-3-galactoside, quercetin-3-galactoside, quercetin-3-glucoside, quercetin-3-xylopyranoside, quercetin-3-arabinopyranosdie, quercetin-3-arabinofuranoside, quercetin-3-rhamnopyranoside, and quercetin aglycone”) and A-type proanthocyanidins (PACs, namely “PAC DP-2 to PAC DP-12”) elicited different cytotoxic effects [160]. However, in SKOV3 cells, DP-9 (50–200 µg/mL) was one of the more potent compounds and decreased p-EGFR levels along with reductions in MEK, p-ERK1/2, and p-c-Raf [160]. In human hepatoma HepG2 cells, the major anthocyanin blueberry component, malvidin-3-galactoside (M3G, which reduced tumor growth in a mouse xenograft model, 40–80 mg/kg), reduced cellular viability, which was accompanied by increased an apoptotic response [161]. These alterations were associated with increased p-p38 and p-JNK, along with reduced p-AKT and increased PTEN expression [161].

*Inflammation:* Methyl salicylate 2-O-β-D-lactoside (MSL, a component from *Gaultheria yunnanensis*, 150–600 mg/kg) reduced hind paw and ankle swelling while reducing inflammation in a rat adjuvant-induced arthritis (AIA) model, which involved subcutaneous (SQ) injection of heat-killed *Myocobacterium butyricum* in one of its hind paws [162]. In addition, using RAW264.7 macrophages treated with LPS, MSL (10–50 µM) mediated protection against the pro-inflammatory response (i.e., reduced PGE2 and COX-2) along with inhibition of p-p38 and p-ERK but not p-JNK [162]. In another study, rhododendrin (20mM, an arylbutanoid glycoside extracted from the powdered leaves of *Rhododendron brachycarpum*) was tested in mice treated with 2,4,6-trinitrochlorobenzene (TNBC) to induce inflammation of the skin on the ears and found to reduce inflammation and epidermal hyperplasia [163]. The mechanism of action was defined by using HaCaT keratinocyte cells stimulated by TNFα/IFNγ treated with rhododendrin (20 µM), which also reduced expression of pro-inflammatory mediators (i.e., IL-1α, IL-1β, IL-6, IL-8, TNFα, and IFNγ) [163]. Furthermore, there was a marked reduction in p-ERK1/2, p-p38, and p-JNK in addition to p-AKT and p-MEK1/2 [163].

### 5.4. Rosaceae Phytochemicals in Signaling

**Figure 11:**
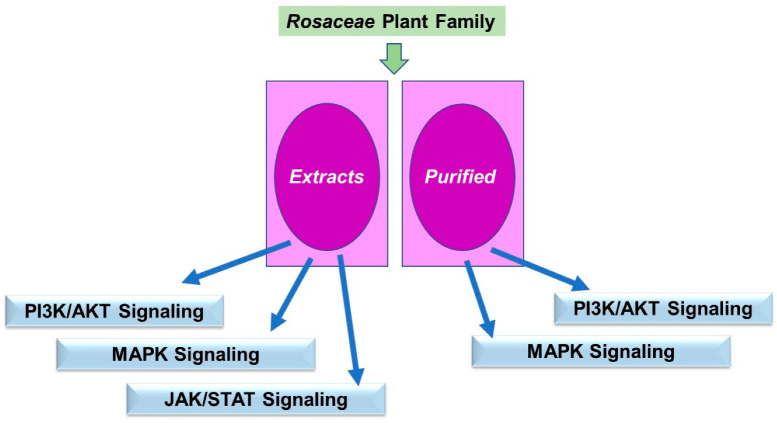
A schematic summary of the effects of metabolites from the *Rosaceae* on the MAPK, PI3K/AKT, and JAK/STAT signal transduction pathways.

A schematic of the key findings is displayed in Figure 11.

#### 5.4.1. Rosaceae Extracts

##### PI3K/AKT/mTOR Signaling

*Blood Cancers:* Using adult MOLT-4 lymphoblastic leukemia cells, extracts from dried fruits of *Rosa cymosa* (RCE, 60 µg/g) reduced tumor volume in an *in vivo* mouse xenograft model [164]. In *in vitro* assays, RCE reduced cellular viability (IC_50_ of 88.8–114.8 µg/mL) with induction of apoptosis [164]. These changes were associated with changes in ER stress markers as well as elevated PTEN, p-PTEN, and p-c-Raf with decreased p-AKT and p-STAT3 proteins [164].

*Solid Tumors:* Multiple polyphenolic fractions from *Kakadu* and *Illawarra* plums (KPF1-8 and IFP1-5, respectively) were tested on various human cell lines [165]. From all the fractions tested, KPF5 (100–400 µg/mL) was determined to be the most potent and modulated expression of pro-inflammatory cytokines (i.e., COX-2, iNOS) in LPS-stimulated RAW264.7 macrophages, which was associated with reduced p-ERK1/2 and p-AKT levels [165]. In A549 cells, different extracts from *Rubus idaeus* L were tested, notably a methanol extract (RIME), chloroform extract (RICE), ethyl acetate extract (RIAE), n-butanol extract (RIBE), and a water extract (RIWE) [166]. RIAE (30 µg/mL) elicited the most potent activity in terms of reducing cellular invasion and migration as well as reducing tumor burden in an *in vivo* xenograft model (50–100 mg/kg) [166]. Along with these functional alterations, RIAE reduced p-AKT together with increased p-GSK3β in the A549 cells [166]. In another study, extracts from lyophilized strawberries (*Fragaria* × *ananassa*) were tested in a mouse model in which colorectal cancer was induced using azoxymethane (AOM) and dextran sodium sulfate (DSS) [167]. The strawberry extract (2.5, 5, and 10%) reduced tumor pathogenesis along with reductions in gene expression of pro-inflammatory markers (i.e., TNFα, IL-1β, IL-6, COX-2, and iNOS) and signaling pathway mediators (i.e., p-PI3K, p-AKT, and p-ERK) [167]. In liver cancer cells, extracts from the fruits from *Rubus idaeus* L (red raspberry, RRE, 25mg/mL) reduced cellular viability with induction of S phase arrest and apoptosis [168]. These changes were associated with increased PTEN expression and decreased p-AKT levels [168].

In lung A549 cells, an extract from the petals of *Rosa gallica* (RPE, 100–400 µg/mL) reduced cellular viability as well as inhibited migration and invasion; these changes were accompanied by reduced expression of p-EGFR, p-c-Raf, p-MEK1/2, p-mTOR, and p-AKT proteins [169]. In this same lung cancer cell line, a polyphenol-enriched plum pulp extract (PPP, 160 µg/mL) from *Wushancuili* elicited a marked reduction in cellular viability, which coincided with reduced p-PI3K and p-AKT levels [170]. Red-flesh (AFP) or peel (APP, phenolic content is higher) component of apples (Meihong variety) was tested on breast cancer cell lines (250–1000 µg/mL); it was identified that the APP was more potent in mediating reduction in cellular viability along with G0/G1 phase arrest and apoptotic response; these functional changes were associated with reduced p-AKT and p-BAD [171]. In human prostate cancer cells, four extracts from the fruits of *Rubus coreanus* Miquel (RCM, 100 µg/mL) (i.e., 50% ethanol extract of unripe RCM (UE), aqueous extract of unripe RCM (UH), 50% ethanol extract of ripe RCM (RE), and aqueous extract of ripe RCM (RH) did not mediate any alteration in cellular viability [172]. However, most of the extracts (except for RE) inhibited cellular migration and invasion, which was accompanied by reduced p-PI3K and p-AKT levels following UA treatment [172]. In HeLa and SiHa cervical cancer cell lines, a polysaccharide extract from *Rosa rugosa* petals (RRP, 800 µg/mL) reduced cellular viability along with induction of apoptosis and autophagy (i.e., increased LC3B-II and reduced p62 levels) [173]. The autophagic-induced cell death response by RRP involved reducing p-AKT and p-mTOR levels [173].

*Tissue Regeneration:* Tissue regeneration using stem cells is a valuable option for therapeutic purposes, such as in the process of aging in which stem cell quality and quantity are reduced [174]. Ethanol extracts from the apple, *Malus pumila* Mill, were tested on adult stem cells from adipose tissue (ADSCs) and cord-blood mesenchyme (CB-MSCs) [174]. Using these cells, the apple extract (0.5–1%) supported stem cell proliferation and cytokine production (i.e., VEGF and IL-6), which were accompanied by activation of p-p70S6K, p-S6RP, p-eIF4E, and p-Raptor [174].

*Inflammation:* Intestinal bowel diseases (IBD) involves the stimulation of microvascular endothelial cells to which circulating immune cells adhere [175]. The effect of a black raspberry extract (BRE, 100 µg/mL) on primary human esophageal microvascular endothelial cells (HEMEC, stimulated with TNFα/IL-1β) reduced gene expression of cell adhesion molecules (i.e., ICAM-1 and VCAM-1), which likely caused diminished adhesion of U937 monocytes [175]. Along with these cellular changes, BRE diminished gene expression of pro-inflammatory mediators (i.e., COX-2 and PGE2 activity) and reductions in VEGF-induced p-AKT in addition to p-ERK1/2 and p-JNK [175]. In another study, total flavonoids (TFs, 50–100 mg/kg, intragastric administration) from the leaves of *Eriobotrya japonica* were tested on mice exposed to cigarette smoke (CS) to examine protective effects and underlying mechanism of action [176]. The treatment with TF reversed the loss of body weight and pulmonary edema; these changes were associated with improved lung histology and lung health as well as reduced levels of pro-inflammatory cytokines (i.e., IL-1β, IL-6, and TNFα in serum) with increased p-AKT and reduced p-JNK levels [176]. The anti-inflammatory activity of a water extract using the dried inner bark of the stems of *Sorbus commixta* (Sc-WE, 100 mg/kg) was tested in an *in vivo* mouse ear edema model; in this system, Sc-WE reduced edema without any cytotoxic response [177]. In the *in vitro* model using macrophages stimulated with LPS, Sc-WE (300 µg/mL) hindered the activation of p-AKT, p-PI3K, and p-PDK1 [177]. Another bark extract from *Prunus jamasakura* (and the purified component, sakuranetin at 60 µM) was tested in rat hepatocytes stimulated with IL-1β and found to reduce pro-inflammatory nitric oxide (NO) production along with diminished levels of p-AKT and IL-1 receptor [178].

*Muscle Aging:* During the process of aging, loss of muscle mass is one characteristic leading to diminished quality of life in the elderly population [179]. A leaf extract from *Eriobotrya japonica* (LE, 50 mg·kg^−1^·day^−1^) was tested on aged rat muscles, and although there was no alteration in body weight, forelimb grip strength was increased together with increased muscle mass [179]. In C2C12 murine myoblasts, LE (0.25–2.5 µg/mL) supported their differentiation without a change in cell survival; these cellular outcomes were accompanied by elevated p-AKT (as well as total AKT) and p-4E-BP1 [179]. Since the quality of life is promoted by maintaining the appropriate mass of skeletal muscles, compounds with a high ursolic acid content, such as that present in an extract from *Aronia melanocarpa* (AME), were tested in rats [180]. Although AME (2.9 g/kg) did not alter muscle weight or muscle protein synthesis after resistance exercise, there were elevated p-AKT levels along with elevated p-ERK1/2, p-mTOR, and p70S6K [180].

*Neurodegeneration Diseases:* The nervous system is the primary accumulation point for heavy metal cadmium toxicity, potentially leading to neurogenerative diseases [181]. The protective action of a polysaccharide ethanol extract (PAP) derived from the roots of *Potentilla anserine* L. was noted in a mouse model, which led to reduced cadmium-induced reductions in food consumption, diarrhea and convulsions, amongst other symptoms of cadmium-induced toxicity [181]. Likewise, in human and murine neuroblastoma cell lines, although PAP did not alter cellular viability (up to 400 g/L) alone, it did promote viability (25 mg/L) against the detrimental effects of cadmium along with a reduction in apoptotic response and reduced ROS levels [181]. Furthermore, PAP reduced cadmium-induced activation of p-AKT and p-mTOR [181].

*Metabolic Diseases:* Extracts prepared from the fruit of the chokeberry (CBE), *Aronia melanocarpa*, were tested *in vivo* (at 100–200 mg·kg^−1^·day^−1^) to determine its efficacy in improving the metabolic condition of rats fed with a high-fructose diet [182]. Together with reduced gain in body weight and adipose tissue, the fasting blood glucose, insulin levels in plasma, triacylglycerides, and total cholesterols were reduced by CBE treatment [182]. In addition, there were diminished levels of the pro-inflammatory cytokines (i.e., IL-6 and TNFα) with altered gene expression of molecules involved in insulin signaling (i.e., elevated PI3KR1 and reduced PTEN RNA) in the epididymal adipose tissues [182]. The anti-diabetic activity of root, fruit, and leaf extracts from *Sarcopoterium spinosum* sp. were tested for their effects on 3T3-L1 pre-adipocytes [183]. Using non-toxic doses of these plant extracts (<1 mg/mL), it mediated glucose uptake (more potent with root and leaf than fruit extracts) as well as inhibition of the PI3K/AKT pathway [183]. In diabetic rats, extracts from fruits (without seeds) of *Crataegus pinnatifida* Bge (HPE, 300 mg/kg) reduced body weights, fasting blood glucose, cholesterol, triacyglyceride, and insulin levels along with improved tissue histology [184]. HPE also reduced the expression of pro-inflammatory mediators (i.e., IL-6 and TNFα) and recovered p-AKT and p-PI3K levels [184]. A methanol extract derived from the aerial components of *Alchemilla monticola* (ALM, 5–25 µg/mL), in which the major components are kaempferol-3-O-glucoside (AST, 5–25 µM) and quercetin-3-O-rhamnoside (QUE, 5–25 µM), were all tested for anti-adipogenic effects in human adipocytes [185]. In a molecular docking simulation, it was noted that AST and QUE had the lowest binding constants with PI3K and PPARγ, which may provide a mechanism of action for these specific metabolites [185]. The ALM extract was reported to diminish the abundance of lipid droplets (LDs) while reducing gene expression of adipogenic genes (i.e., CEBPA and PPARG) [185]. The ALM extract was also the most potent in reducing the protein levels of AKT and PI3K [185].

##### JAK/STAT Signaling

*Inflammation:* In LPS-activated macrophages, a water extract from the unripe fruits of *Rubus coreanus* Miquel (RF) reduced p-p38 levels and NO generation [186]. Associated with this response, RF diminished the gene expression of the ER stress marker, CHOP, as well as STAT1, STAT3, and JAK2, in the absence of cytotoxicity (25–200 µg/mL) [186]. In another study using HaCaT cells, a water extract from *Sanguisorbae Radix* (WSR) did not mediate any change in cellular viability (up to 50 µg/mL) with only a subtle effect at a higher dose (100 µg/mL) [187]. In response to TNFα/IFNγ stimulation, WSR (low doses) inhibited pro-inflammatory chemokine expression (i.e., TARC, RANTES, MDC, and IL-8) and diminished expression of p-JAK2 and p-STAT1 [187]. An ethanol extract from black raspberry powder (BRB-E, containing diverse phytochemical constituents, 200 µg/mL) from *Rubus occidentalis* was tested on CD4+ and CD8+ T cells; in response to CD3/CD28 activation, BRB-E reduced cellular viability, as well as IL-6, mediated p-STAT3 and IL-2 mediated p-STAT5 levels [188].

##### MAPK Signaling

*Solid Tumors:* Pre-treatment of human lung cancer cells (A549) with strawberry extracts (*Fragaria* x *ananassas* cv. *Earliglow*, diluted at 1:250–1:500) resulted in a reduction in cellular viability as well as antagonism towards ultraviolet irradiation (UVB)-induced activation of p-ERK and p-JNK with no change in p-p38 [189]. In B16 murine melanoma cells, a methylene chloride fraction (1mg/mL, from Whole *Geum japonicum* Thunberg plant powder) inhibited cell attachment and migration; this fraction also inhibited angiogenesis in HUVEC endothelial cells [190]. Together with these cellular outcomes, this fraction reduced gene expression of CD44 (involved in tumorigenic response) and TIMP2 (involved in activation of MMP-2) along with increased p-JNK and p-p38 but no change in p-ERK [190]. In HepG2 cells, three different extracts from different plum (*Prunus salicina* Lindl cv Soldam) stages of maturity (immature extract (IPE), mid mature extract (MMPE), and mature extract (MPE)) were tested [191]. Phorbol ester (PMA) induction of cellular migration, as well as MMP-9 (expression and activity), were markedly reduced by IPE (12.5–100 µg/mL) [191]. In addition, other alterations by IPE in PMA-treated HepG2 cells included reduced p-p38, p-JNK, and p-ERK levels [191]. The effect of total phenolics from dark sweet cherries (WE) along with enriched fractions of anthocyanins (ACN) and proanthocyanidins (PCA) were tested towards their anti-neoplastic effects using breast cancer MDA-MB-453 cells in an *in vivo* mouse xenograft model [192]. In this regard, treatments with these agents (150 mg/kg) reduced tumor growth along with elevated levels of p-ERK1/2 [192]. With ACN, there were enhanced anti-neoplastic effects via downregulation in the expression of signaling proteins (i.e., AKT, STAT3, p38, and JNK) [192]. By using these same breast cancer cells, the dark sweet cherry anthocyanin-enriched phenolics (WE, 83 µg/mL) along with ACN (anthocyanin-rich, 19 µg/mL) and PCA (proanthocyanidin-rich, 22.5 µg/mL) from *Prunus avium* were found to reduce p-AKT while increasing p-p38, p-JNK, and p-ERK1/2 [193]. In the HT1080 fibrosarcoma cell line, a methanolic extract from *Agrimona Pilosa* Ledeb (APLME, 20–80 µg/mL) reduced cellular viability and invasion upon VEGF stimulation; these cellular outcomes also reduced MMP-2 and MMP-9 levels [194]. Furthermore, the levels of p-ERK and p-JNK were reduced following stimulation with PMA, as a tumor promoting agent [194]. In colorectal SW-480 and HT-29 cancer cells, a leaf extract derived from *Chaenomeles japonica* L. (PRE) inhibited migration and invasion by reducing the activities of MMP-2 and MMP-9 [195]. These changes were accompanied by diminished levels of p-ERK and p-AKT in SW-480 but increased p-ERK with reduced p-AKT in HT-29 cells [195].

*Cardiovascular Diseases:* Fruit juice concentrate from Asian plum, Bainiku-ekisu, was tested on angiotensin II (AngII, a vasoconstrictor)-induced EGF receptor transactivation and downstream signaling events in vascular smooth muscle cells (VSMCs) derived from rat thoracic aorta [196]. Pre-treatment with Bainiku-ekisu (1mg/mL) reduced AngII-induced (and H_2_O_2_-induced) EGF receptor transactivation without altering total EGFR protein levels in addition to reduced p-ERK1/2 levels [196].

*Inflammation:* With attempts to uncover dietary considerations for intestinal bowel diseases (IBD), apple powder (from the skin and pulp) from two varieties of apples (Marie Menard and Golden Delicious) were tested in a mouse model of colitis (HLA-B27 transgenic rats, characterized by intestinal inflammation with histological alterations similar to human IBD) [197]. After a 12-week treatment period, symptoms of IBD were reduced (i.e., diarrhea) with extensive changes in gene expression patterns (i.e., reduction in MAPK pathway, TNFα-NFkB), which were more marked with the Marie Menard variety [197]. An apple polyphenol extract (APP) was tested to identify whether it could mediate protection against cigarette-smoke-induced lung injury and inflammation using mice exposed to cigarette smoke [198]. APP treatment (up to 300 mg/kg) via intragastric administration diminished the inflammatory cell infiltration in the lung tissue with improved lung tissue histology [198]. At a cellular level, APP treatment reduced gene expression of the pro-inflammatory cytokines (i.e., TNFα and IL-1β) and reduced p-p38, as noted in histochemical sections [198]. In LPS-stimulated mice, extracts from the bark of *Prunus yedoensis* (PYE, (10, 50, 250 mg/kg) markedly reduced pro-inflammatory cytokines in serum (i.e., TNFα and IL-6) [199]. This supports findings from *in vitro* macrophage studies wherein PYE (up to 200 µg/mL) also reduced the gene expression of these cytokines induced by LPS, which were accompanied by reduced levels of p-p38, p-JNK, and p-ERK1/2 [199].

The anti-inflammatory potential of extracts from the berries of *Crataegus laevigata* (CLE) was tested on HaCaT cells [200]. In the absence of any changes in cytotoxicity from CLE (10–100 µg/mL) with LPS-stimulated HaCaT cells, CLE reduced the expression of pro-inflammatory cytokines (i.e., IL-8 and TARC) as well as p-p38, p-ERK, and p-JNK [200]. HaCaT cells treated with TNFα/IFNγ were also utilized to test anti-inflammatory responses with extracts from the leaves of the *Rosa davurica* Pall plant (RDL) [201]. RDL (100 µg/mL) did not alter cellular viability in cells, but it did suppress TARC and IL-6 secretion along with p-p38, p-JNK, and p-ERK [201]. The anti-inflammatory action of the peel extract from *Cydonia oblonga* miller was tested in THP-1 human myelomonocytic cells [202]. Although the extract (20 µg/mL) did not alter cellular viability, it diminished LPS-stimulated secretion of TNFα (partly via IL-6), IL-10, and IL-8 [202]. The signaling cascade mediated through this extract was the inhibition of p-p38 and p-AKT [202]. The skin moisturizing and anti-inflammatory responses of an extract from the whole plant of *Filipendula palmata* (FPE) were tested on HaCaT cells [203]. Although FPE (10–200 µg/mL) did not alter cellular viability, it reduced ROS generation when cells were stimulated with TNFα/IFNγ [203]. Furthermore, FPE inhibited the gene expression of pro-inflammatory cytokines (IL-8 and TARC) as well as promoted elevated levels of hyaluronic acid (HA) [203]. FPE also inhibited the p-ERK1/2, p-JNK, and p-p38 levels [203].

*Allergic Responses:* In addition to unripe fruit extracts of the black raspberry plant, namely *Rubus coreanus* Miquel, that antagonize allergic responses, ripe fruit extracts from this plant (RFRC) were also tested in an animal model [204]. In the *in vivo* mouse model, mortality was reduced when the mast cell degranulation compound 48/80 was intraperitoneally injected following anal administration of RFRC (10–1000 mg/kg) [204]. Likewise, in human mast cell lines, RFRC (100 µg/mL) inhibited the release of histamine, intracellular calcium, and pro-inflammatory cytokine expression and secretion (i.e., TNFα, IL-1β, IL-6) [204]. Furthermore, there was a notable reduction in p-p38, p-JNK, and p-ERK upon mast cell activation [204].

*Neurodegenerative Disease:* A total flavonol extract (TFs) from *Rosa laevigata* Michx was tested for its ability to mediate protection against neurodegenerative diseases [205]. Specifically, TFs were examined with respect to protection against oxidative injury in which hydrogen peroxide can be detrimental to neuronal health by inducing cell death [205]. In this regard, H_2_O_2_-stimulated pheochromocytoma PC-12 cells treated with TFs (100–500 µg/mL) recovered cell health in addition to the reduction in pro-inflammatory cytokine production (i.e., IL-1, IL-6, and TNFα) and reduction in p-ERK, p-JNK, and p-p38 levels [205].

In an animal model subjected to BCCAo (bilateral common carotid artery occlusion) in which there is reduced cerebral blood flow leading to neurological complications, an extract from unripe fruit of *Prunus mume* (200 mg·kg^−1^·day^−1^) was tested [206]. The extract decreased both microglial and astrocytic activation with a reduction in pro-inflammatory mediators (i.e., COX-2, IL-1β, and IL-6) protein expression and a reduction in p-p38 activation in the hippocampus [206].

*Liver Injury:* Carbon tetrachloride (CCl_4_)-induced liver injury is associated with the induction of oxidative stress, and thus, antioxidants arising from phytochemicals may provide some health benefits to hinder the detrimental effects associated with this injury [207]. In this regard, extracts from the fruits of *Chaenomeles thibetica* (CTE, 40–100 mg/kg) were tested in a rat model of CCl_4_-induced liver injury [207]. CTE elevated glutathione (GSH) levels, as well as decreased liver enzymes (ALP) and total bilirubin, as a marker of liver function, with decreased histological evidence of liver damage [207]. Along with these outcomes, CTE was noted to recover cellular viability (up to 250 µg/mL) as well as elevate p-JNK, p-ERK, and p-p38 levels in HepG2 cells treated with CCl_4_ [207].

*Skin Aging:* Photoaging of the skin, a critical barrier between organs and environment, can be induced by ultraviolet B irradiation (UVB) [208]. Ethanol extracts from the twigs of *Sorbus commixta* (STE) were found to mediate protection from UVB-stimulated human dermal fibroblast cells (NHDF); STE (200 µg/mL) treatment increased cellular viability, reduced ROS generation, and protein expression of secreted MMP-1, MMP-2, and MMP-3 [208]. These changes were accompanied by reduced p-p38, p-ERK, and p-JNK (and total JNK) [208]. Extracts from cherry blossoms of *Prubus yeonesis* (CBE) were also tested on UVB-irradiated NHDFs [209]. In the absence of any alteration in cellular viability, CBE (100 µg/mL) reduced UVB-induced gene expression of MMP-1 and MMP-3 along with increased type I pro-collagen RNA levels [209]. These changes were accompanied by reduced p-ERK, p-JNK, and p-p38 [209]. Furthermore, an extract from the dried fruit of *Rubus idaeus* L (RI, 1–100 µg/mL) also increased cellular viability, reduced ROS production, and levels of secreted MMP-1 and IL-6 along with increased pro-collagen type I RNA [210]. These changes were accompanied by reduced p-ERK, p-JNK, and p-p38 under these conditions [210].

Polyphenol extracts from the fruits of *Crataegus pinnatifida* (HPE) were also tested for their protective benefit towards UVB-induced photoaging on dermal fibroblasts and keratinocytes (HDF and HaCaT cells) [211]. HPE was noted to recover UVB-induced reduction in cellular viability (5–10 µg/mL) along with reducing ROS production and MMP protein production [211]. Pro-collagen type I protein expression was also elevated with HPE treatments [211]. In an *in vivo* mouse study wherein mice were orally exposed to HPE (100 or 300 mg·kg^−1^·day^−1^) along with UVB irradiation [211], the extract reduced MMP-1, MMP-3, and MMP-9 with improvements in histology (i.e., reversal of elastic fiber thickening, disorganization, and hyperplasia along with improved skin moisture) [211]; moreover, HPE reduced UVB-induced p-p38, p-ERK, and p-JNK levels [211]. Extracts prepared from the leaves of *Pourthiaea villosa* (PVDE, 50–100 µg/mL) were tested on HDFs and found to mediate protection against H_2_O_2_-induced cell death [212]. PVDE also reduced ROS production, which occurred along with the reduction in MMP-2, MMP-3, and MMP-9 activities as well as p-p38 and p-JNK (although p-ERK was elevated) [212]. Ethanol extracts from *Potentilla glabra* (Pg-EE) were also tested in HaCaT cells in response to UVB-induced photoaging [213]. With only a subtle growth-promoting response on cellular viability of Pg-EE (200 µg/mL), there was the diminished expression of pro-inflammatory mediators (i.e., IL-1β and IL-6), elevated RNA gene expression of the skin barrier and hydration factors, as well as reduced levels of p-ERK1/2 and p-p38 levels [213].

*Hair Growth:* An extract prepared from *Crataegus pinnatifia* fruits was tested on human dermal papilla cells (hDPCs) to investigate its effects on modulating hair growth, which may offer some benefit to improving blood circulation to the hair follicle to stimulate its growth [214]. While the extract increased the proliferative capacity (1 µg/mL, 40%), it also elevated p-p38, p-ERK, and p-JNK, as well as p-AKT [214].

#### 5.4.2. Rosaceae Purified Phytochemicals

##### PI3K/AKT/mTOR Signaling

*Solid Tumors:* In an array of cancer cell lines, a compound isolated from *Potentilla discolor* Bunge, namely PDB-1 (C-27-carboxylated-lupane-triterpenoid derivative), was found to reduce cellular viability (most potent at IC_50_ of 7.8 µM in A549) along with induction of G2/M phase arrest; these cellular outcomes were associated with reduced p-PI3K, p-mTOR, and p-AKT levels [215]. A compound (ellagic acid) isolated from an ethanol extract from the root of *Sanguisorba officinalis* was tested on murine melanoma cells (B16F10) to uncover its mechanism of action [216]. Along with its growth-reducing potential (100–300 µg/mL), it induced apoptosis and G1 arrest, which was associated with reduced levels of p-AKT, p-p70S6K, and p-ERK1/2 along with increased PTEN activity [216].

*Cardiovascular Diseases:* Pentacyclic triterpenoids, euscaphic acid (EA), and tormentic acid (TA) were isolated from roots from *Potentilla anserine* L and tested in EA.hy926 cells, a human umbilical vein endothelial cell line [217]. While both agents protected the cells against hypoxia-induced cellular damage, they mediated differential responses in signaling events wherein EA induced p-ERK1/2 (with reduced p-AKT) and TA activated both p-ERK1/2 and p-AKT [217].

##### MAPK Signaling

*Blood Cancers:* One component from the Jewel black raspberry extract, namely cyanidin-3-rutinoside (C3R, 50 µM), reduced cellular viability via induction of apoptosis in leukemic HL-60 cells; these cellular outcomes were associated with elevated p-p38 and p-JNK levels [218].

*Inflammation:* The critical cellular component of articular cartilage, namely chondrocytes, contributes to the pathogenesis of osteoarthritis (OA) in which IL-1β is a critical contributor to OA development; antagonizing IL-1β activities may be of benefit to these patients [219]. One compound from *Rosa agrestis*, namely astragalin, did not affect cellular viability (20–80 µg/mL) in these cells, but it did reduce IL-1β-induced responses, including expression of pro-inflammatory mediators (i.e., NO and PGE2) as well as p-ERK1/2, p-JNK, and p-p38 levels [219]. In A549 cells, the apple tree flavonoid phloretin was also tested for its effects on eliciting anti-inflammatory responses [220]. In response to IL-1β stimulation, phloretin (3–100 µM) reduced the expression of pro-inflammatory cytokines (i.e., PGE2, COX-2, IL-8, and IL-6) [220]. Furthermore, these changes were accompanied by reduced levels of p-AKT, p-ERK, p-JNK, and p-p38 [220]. Since inappropriate activation of human neutrophils can lead to damage to tissues along with the pathogenesis of the disease, finding therapies to hinder their aberrant activation would be of high clinical benefit [221]. In *in vitro* studies, N-formylated peptides (fMLP, which activates GPCRs) were utilized to simulate neutrophil activation and examine protection mediated by 2′-3-dihydroxy-5-methoxybiphenyl (RIR-2), which is obtained from a methanol extract derived from the roots of a variety of *Rhaphiolepsis indica* [221]. In normal human neutrophils, RIR-2 (IC_50_ of 2.57 µM) diminished fMLP-stimulated migration with no change in cellular viability [221]. Interestingly, although RIR-2 did not alter ligand-binding to the receptor, it did hinder the interaction between G_i_β and p-Src and with PLC, and moreover, RIR-2 diminished fMLP-induced activation of p-p38, p-AKT, and p-ERK along with reduced p-PLC and p-PKC [221].

*Skeletal Maintenance:* Maintenance of the skeleton is critical in post-menopausal women, who commonly undergo hormone therapy to oppose osteoporosis [222]. However, this treatment regimen is associated with an increased risk of myocardial infarctions, strokes, and cancer [222]. In this regard, using calvarial osteoblasts isolated from rats, prunetin (from *Prunus avium*) increased cellular proliferative indices (0.1 nM–1 µM) and osteoblast differentiation along with induction of genes involved in osteogenesis (i.e., RUNX2, OCN, Col-1). These alterations were dependent on cAMP production [222], while the increase in RUNX2 was dependent on activation of the MAPK signaling cascade (pMEK1/2 and p-ERK1/2) through adenylyl cyclase (AC) and increased G-protein coupled receptor (GPR30) expression [222].

*Cardiovascular Disease:* Vascular diseases (such as atherosclerosis) are characterized by increased proliferation of vascular smooth muscle cells and alterations in the extracellular matrix [223]. In human aortic smooth muscle cells (HASMCs, TNFα stimulated), a purified component from *Geum japonicum,* namely trihydroxybenzaldehyde (THBA, up to 500 µg/mL) reduced cellular viability while reducing activities and expression of MMP-2 and MMP-9; these expression changes were accompanied by reduced cellular migration as well as reduced p-ERK1/2, p-p38, and p-JNK levels [223].

**Table 6:** Summary of Effects on Signaling across the three plant families.

Effects on Signaling			
*Cucurbitaceae*			
Plant Metabolite	Signaling Pathway	Associated Disease Model	References
CuA	PI3K/AKT/mTOR	Solid Tumors	[73,74]
CuB	PI3K/AKT/mTOR	Cardiac Hypertrophy	[75]
CuB	PI3K/AKT/mTOR	Solid Tumors	[76,77,78,79,80,81,82]
CuB	JAK/STAT	Solid Tumors	[83,84,85,86,87,88]
CuB	MAPK	Solid Tumors	[89,90]
CuC	JAK/STAT	Solid Tumors	[92]
CuD	PI3K/AKT/mTOR	Solid Tumors	[93]
CuD	MAPK	Blood Cancers	[94]
CuD	MAPK	Solid Tumors	[95]
CuE	PI3K/AKT/mTOR	Solid Tumors	[96,97]
CuE	JAK/STAT	Solid Tumors	[98]
CuE	JAK/STAT	Neovascularization	[99]
CuE	MAPK	Solid Tumors	[100,101]
CuI	PI3K/AKT/mTOR	Solid Tumors	[102]
CuI	JAK/STAT	Blood Cancers	[103]
CuI	JAK/STAT	Solid Tumors	[104,105,106,107,108,109,110,111]
CuI	MAPK	Blood Cancers	[112]
CuI	MAPK	Solid Tumors	[113,114,115]
CuI	MAPK	Cardiac	[116]
CuQ	JAK/STAT	Solid Tumors	[117]
Extract from leaves of *C. colocynthis* (L.) Shrad	JAK/STAT and AKT	Solid Tumors	[118]
DHCE	MAPK	Solid Tumors	[119]
CuIIa	MAPK	Solid Tumors	[120]
CuIIb	MAPK	Solid Tumors	[121]
***Ericaceae***			
**Plant Metabolite**	**Signaling Pathway**	**Associated Disease Model**	**References**
Antho 50 (Bilberry extract from *Vaccinium myrtillus* L)	PI3K/AKT/mTOR	Blood Cancers	[122]
Enriched fraction of cranberry oligomeric proanthocyanidins (PAC-1A)	PI3K/AKT/mTOR	Solid Tumors	[123]
Cranberry proanthocyanidin extract (PAC-1)	PI3K/AKT/mTOR	Solid Tumors	[124]
Cranberry proanthocyanidin extract (CPAC)	PI3K/AKT/mTOR	Solid Tumors	[125]
Whole cranberry extracts from berry juice (CB)	PI3K/AKT/mTOR	Blood Cancers	[126]
Whole cranberry powder from *Vaccinium macrocarpon* (WCP)	PI3K/AKT/mTOR	Solid Tumors	[127]
Blueberry extract (BE)	PI3K/AKT/mTOR	Solid Tumors	[128]
Whole blueberry extract from *Vaccinium angustifolium*	PI3K/AKT/mTOR	Solid Tumors	[129]
Blueberry supplement from *Vaccinium myrtillus*	PI3K/AKT/mTOR	Solid Tumors	[130]
Anthocyanins (ACN)	PI3K/AKT/mTOR	Solid Tumors	[131]
Phenolic acids (PA)	PI3K/AKT/mTOR	Solid Tumors	
Bilberry extract (BE)	PI3K/AKT/mTOR	Cardiovascular Disease	[132]
Bilberry anthocyanins (BA)	PI3K/AKT/mTOR	Perimenopause	[133]
Anthocyanin-enriched blueberry extract (BAE)	PI3K/AKT/mTOR	Metabolic Disorder	[134]
Blueberry anthocyanin extract (BAE)	PI3K/AKT/mTOR	Metabolic Disorder	[135]
Blueberry anthocyanin extract	PI3K/AKT/mTOR	Macular Degeneration	[136]
Malvidin (Mv)	PI3K/AKT/mTOR	Macular Degeneration	
Malvidin-3-glucoside (Mv-3-glc)	PI3K/AKT/mTOR	Macular Degeneration	
Malvidin-3-galactoside (Mv-3-gal)	PI3K/AKT/mTOR	Macular Degeneration	
Blueberries (BB)	PI3K/AKT/mTOR	Cognitive Function	[137]
Rabbit-eye blueberry leaf fractions from *Vaccinium virgatum* Aiton	JAK/STAT	Blood Cancers	[138]
Blueberry supplementation	JAK/STAT	Solid Tumors	[139]
Malvidin (Mv)	JAK/STAT	Solid Tumors	
*Rhododendron album blume* methanol extract (RAME)	JAK/STAT	Inflammation	[140]
Extracts from chokeberry (*Aronia melanocarpa*)	MAPK	Solid Tumors	[141]
Extracts from elderberry (*Sambicus nigra*)	MAPK	Solid Tumors	
Extracts from bilberry (*Vaccinium myrtillus*)	MAPK	Solid Tumors	
Microencapsulated form of bilberry extract	MAPK	Solid Tumors	[142]
Mixture of 15 anthocyanins from bilberries	MAPK	Solid Tumors	[143]
Polyphenol-enriched blueberry preparation (PEBP) from *Vaccinium angustifolium* Ait juice	MAPK	Solid Tumors	[144]
Lingonberry extracts	MAPK	Solid Tumors	[145]
Ethanol extracts from stems (VOS) of *Vaccinium oldhamii* Miquel	MAPK	Inflammation	[146]
Ethanol extracts from leaves (VOL) of *Vaccinium oldhamii* Miquel	MAPK	Inflammation	
Ethanol extracts from fruits (VOF) of *Vaccinium oldhamii* Miquel	MAPK	Inflammation	
Anthocyanins extracted from blueberry (*Vaccinium* sp.) (BE)	MAPK	Inflammation	[147]
Anthocyanin extract from bog blueberry (ATH-Bbe)	MAPK	Inflammation	[148]
High-molecular weight non-dialyzable material (NDM) from cranberry juice of *Vaccinium macrocarpon*	MAPK	Inflammation	[149]
Mixture of 7 phenolic acids (7PA)	MAPK	Inflammation	[150]
Whole extract (TifBlue BB) from *Vaccinium virgatum*	MAPK	Cognitive Function	[151]
Fractions from wild blueberry juice from *Vaccinium angustifolium* Aiton	MAPK	Cognitive Function	
Blueberry supplementation (BB)	MAPK	Cognitive Function	[152]
Pterostilbene (Pter)	PI3K/AKT/mTOR	Solid Tumors	[153,154]
Cinnamtannin D1 (CNT D1)	PI3K/AKT/mTOR	Solid Tumors	[155]
Rhodomeroterpene (RMT)	PI3K/AKT/mTOR	Inflammation	[156]
Hyperoside	PI3K/AKT/mTOR	Inflammation	[157]
Malvidin (Mv)	PI3K/AKT/mTOR	Metabolic Disorder	[158]
Malvidin-3-glucoside (Mv-3-glc)	PI3K/AKT/mTOR	Metabolic Disorder	
Malvidin-3-galactoside (Mv-3-gal)	PI3K/AKT/mTOR	Metabolic Disorder	
Cya-3-Ara	PI3K/AKT/mTOR	Metabolic Disorder	[159]
Purified cranberry flavonols and A type proanthocyanidins (PACs)	MAPK	Solid Tumors	[160]
Malvidin-3-galactoside (M3G)	MAPK	Solid Tumors	[161]
Methyl salicylate 2-O-b-D-lactoside (MSL)	MAPK	Inflammation	[162]
Rhododendrin	MAPK	Inflammation	[163]
***Rosaceae***			
**Plant Metabolite**	**Signaling Pathway**	**Associated Disease Model**	**References**
Extracts from dried fruits of *Rosa cymosa* (RCE)	PI3K/AKT/mTOR	Blood Cancers	[164]
Multiple polyphenolic fractions from *Kakadu* and *Illawarra* plums	PI3K/AKT/mTOR	Solid Tumors	[165]
Methanol extract (RIME) from *Rubus idaeus* L	PI3K/AKT/mTOR	Solid Tumors	[166]
Chloroform extract (RICE) from *Rubus idaeus* L	PI3K/AKT/mTOR	Solid Tumors	
Ethyl acetate extract (RIAE) from *Rubus idaeus* L	PI3K/AKT/mTOR	Solid Tumors	
N-butanol extract (RIBE) from *Rubus idaeus* L	PI3K/AKT/mTOR	Solid Tumors	
Water extract (RIWE) from *Rubus idaeus* L	PI3K/AKT/mTOR	Solid Tumors	
Extracts from lyophilized strawberries (*Fragaria x ananassa*)	PI3K/AKT/mTOR	Solid Tumors	[167]
Extracts from red raspberry fruits of *Rubus idaeus* L (RRE)	PI3K/AKT/mTOR	Solid Tumors	[168]
Extract from petals of *Rosa gallica* (RPE)	PI3K/AKT/mTOR	Solid Tumors	[169]
Polyphenol-enriched plum pulp extract (PPP) from *Wushancuili*	PI3K/AKT/mTOR	Solid Tumors	[170]
Red flesh component of apples (Meihong variety) (AFP)	PI3K/AKT/mTOR	Solid Tumors	[171]
Peel component of apples (Meihong variety) (APP)	PI3K/AKT/mTOR	Solid Tumors	
Extracts from fruits of *Rubus coreanus* Miquel (RCM): 50% ethanol extract from unripe RCM (UE), aqueous extract of unripe RCM (UH), 50% ethanol extract of ripe RCM (RE), and aqueous extract of ripe RCM (RH)	PI3K/AKT/mTOR	Solid Tumors	[172]
Polysaccharide extract from *Rosa rugosa* petals (RRP)	PI3K/AKT/mTOR	Solid Tumors	[173]
Ethanol extracts from apple *Malus pumila* Mill	PI3K/AKT/mTOR	Tissue Regeneration	[174]
Black raspberry extract (BRE)	PI3K/AKT/mTOR	Inflammation	[175]
Total flavonols (TFs) from leaves of *Eriobotrya japonica*	PI3K/AKT/mTOR	Inflammation	[176]
Water extract from dried inner bark of stems of *Sorbus commixta* (Sc-WE)	PI3K/AKT/mTOR	Inflammation	[177]
Bark extract from *Prunus jamasakura*	PI3K/AKT/mTOR	Inflammation	[178]
Leaf extract from *Eriobotrya japonica* (LE)	PI3K/AKT/mTOR	Muscle Aging	[179]
Extract from *Aronia melanocarpa* (AME)	PI3K/AKT/mTOR	Muscle Aging	[180]
Polysaccharide ethanol extract (PAP) from roots of *Potentilla anserine L*	PI3K/AKT/mTOR	Neurodegenerative Disease	[181]
Extracts from fruit of chokeberry (CBE) from *Aronia melanocarpa*	PI3K/AKT/mTOR	Metabolic Disorder	[182]
Root, fruit, and leaf extracts from *Sarcopoterium spinosum Sp*	PI3K/AKT/mTOR	Metabolic Disorder	[183]
Extracts from fruits (without seeds) of *Crataegus pinnatifida Bge* (HPE)	PI3K/AKT/mTOR	Metabolic Disorder	[184]
Methanol extract from aerial components of *Alchemilla monticola* (ALM)	PI3K/AKT/mTOR	Metabolic Disorder	[185]
Kaempferol-3-O-glucoside (AST)	PI3K/AKT/mTOR	Metabolic Disorder	
Quercetin-3-O-rhamnoside (QUE)	PI3K/AKT/mTOR	Metabolic Disorder	
Water extract from unripe fruits of *Rubus coreanus* Miquel (RF)	JAK/STAT	Inflammation	[186]
Water extract from *Sanguisorbae Radix* (WSR)	JAK/STAT	Inflammation	[187]
Ethanol extract from black raspberry powder (BRB-E) from *Rubus occidentalis*	JAK/STAT	Inflammation	[188]
Strawberry extracts (*Fragaria x ananassas cv. Earliglow*)	MAPK	Solid Tumors	[189]
Methylene chloride fraction from *Geum japonicum Thunberg*	MAPK	Solid Tumors	[190]
Extracts from different stages of plum maturity (*Prunus salicina* Lindl cv Soldam): immature extract (IPE), midmature extract (MMPE), mature extract (MPE)	MAPK	Solid Tumors	[191]
Total phenolics from dark sweet cheries (WE)	MAPK	Solid Tumors	[192,193]
Enriched fractions of anthocyanins (ACN) and proanthocyanidins (PCA)	MAPK	Solid Tumors	
Methanol extract from *Agrimona Pilosa Ledeb* (APLME)	MAPK	Solid Tumors	[194]
Leaf extract from *Chaenomeles japonica L* (PRE)	MAPK	Solid Tumors	[195]
Fruit juice concentrate from Asian plum (Bainiku-ekisu)	MAPK	Cardiovascular Disease	[196]
Apple powder (skin and pulp) from two varieties of apples (Marie Menard and Golden Delicious)	MAPK	Inflammation	[197]
Apple polyphenol extract (APP)	MAPK	Inflammation	[198]
Extracts from the bark of *Prunus yedoensis* (PYE)	MAPK	Inflammation	[199]
Extracts from berries of *Crataegus laevigata* (CLE)	MAPK	Inflammation	[200]
Extracts from leaves of *Rosa davurica* Pall (RDL)	MAPK	Inflammation	[201]
Peel extract from *Cydonia oblonga* miller	MAPK	Inflammation	[202]
Extract from whole plant of *Filipendula palmata* (FPE)	MAPK	Inflammation	[203]
Ripe fruit extracts from *Rubus coreanus* Miquel (RFRC)	MAPK	Allergic Responses	[204]
Total flavonol extract (TFs) from *Rosa laevigata* michx	MAPK	Neurodegenerative Disease	[205]
Extract from unripe fruit of *Prunus mume*	MAPK	Neurodegenerative Disease	[206]
Extracts from fruits of *Chaenomeles thibetica* (CTE)	MAPK	Liver Injury	[207]
Ethanol extracts from twigs of *Sorbus commixta* (STE)	MAPK	Skin Aging	[208]
Extracts from cherry blossoms of *Prubus yeonesis* (CBE)	MAPK	Skin Aging	[209]
Extract from dried fruit of *Rubus idaeus L* (RI)	MAPK	Skin Aging	[210]
Polyphenol extracts from fruits of *Crataegus pinnatifida* (HPE)	MAPK	Skin Aging	[211]
Extracts from leaves of *Pourthiaea villosa* (PVDE)	MAPK	Skin Aging	[212]
Ethanol extracts from *Potentilla glabra* (Pg-EE)	MAPK	Skin Aging	[213]
Extract from *Crataegus pinnatifa* fruits	MAPK	Hair Growth	[214]
PDB-1 (C-27-carboxylated-lupane-triterpenoid derivative)	PI3K/AKT/mTOR	Solid Tumors	[215]
Ellagic acid	PI3K/AKT/mTOR	Solid Tumors	[216]
Euscaphic acid (EA)	PI3K/AKT/mTOR	Cardiovascular Disease	[217]
Tormentic acid (TA)	PI3K/AKT/mTOR	Cardiovascular Disease	
Cyanidin-3-rutinoside (C3R)	MAPK	Blood Cancers	[218]
Astragalin	MAPK	Inflammation	[219]
Phloretin	MAPK	Inflammation	[220]
2′-3-dihydroxy-5-methoxybiphenyl (RIR-2)	MAPK	Inflammation	[221]
Prunetin	MAPK	Skeletal Maintenance	[222]
Trihydroxybenzaldehyde (THBA)	MAPK	Cardiovascular Disease	[223]

## 6. Concluding Perspectives

### 6.1. Cytoskeletal Alterations—Future Perspectives and Gaps in Knowledge

In all of the above-described CuB studies, the cytoskeletal alterations were mostly focused on changes in actin filaments. Further efforts are needed to investigate CuB’s effects on microtubules and intermediate filaments, including the detailed mechanism of action. These cytoskeletal events occurred within a short time period (minutes to a few hours), which is in contrast to the slow responses that are induced to mediate functional responses such as cellular growth, migration, invasion, and tumor growth (24 h to a few days). Further evidence using targeting strategies (i.e., siRNA) against proteins involved in regulating the cytoskeletal network would provide evidence of their contribution to the observed cellular outcomes (cell death, tumor reduction, migration, invasion, etc.) in response to the cucurbitacins. Apart from evidence associating CuB-induced changes in cytoskeletal alterations across all the above-described cancer cell types, there is limited data on the mechanism of these changes, with the exception of one report presenting evidence of the involvement of a PKA-dependent pathway leading to VASP activation required for F-actin aggregate formation [28]. Since PKA is downstream of G-protein coupled receptors (GPCRs), a current gap in knowledge is the identity of such cell-surface GPCRs that may become activated in response to CuB exposure. As reviewed in [18], there exist numerous regulatory proteins which participate in cytoskeletal organization, and further research could be focused on how CuB exposure might regulate their activities.

The majority of the studies with CuE appear to be conducted at longer time periods (i.e., 24 h), which contrasts with the shorter time course performed with CuB. Along with limited data in blood cancers, there is yet again little to no examination of the detailed mechanism of action of CuE on modulating cytoskeletal elements. Therefore, further efforts could focus on comparing the efficacies and detailed mechanistic contribution to these descriptive intracellular cytoskeletal outcomes. Although it was demonstrated that human glioma tumor (GBM) specimens are characterized by an upregulated cofilin pathway including elevated LIMK1/2 (relative to the normal brain) [224], and CuI-treated GBM cell lines led to multiple altered cellular outcomes (i.e., cytotoxicity, adhesion, migration, and invasion), no further mechanistic insights into cytoskeletal alterations induced by cucurbitacins were established, which would be a noteworthy future direction. Furthermore, such as CuE, there is limited data for CuI in both blood and solid cancers, along with mechanisms of action on actin filaments. Further efforts can investigate alterations in microtubules and intermediate filaments in response to CuI as well. There is also limited data in both blood and solid cancers with respect to cucurbitacin derivatives, along with their mechanisms of action on the cytoskeletal network. Further efforts can investigate such alterations in actin, microtubules, and intermediate filaments in response to these cucurbitacin derivatives as well.

Farrerol, a flavanone in the *Ericaceae* family from *Rhododendron dauricum* L., elicits functional outcomes by modulating numerous signaling cascades, including MAPK and AKT, amongst others [225]. However, to the best of our knowledge, there are no published reports of its effects on the cytoskeleton. Little to no mechanistic insights into cytoskeletal alterations induced by phytochemicals from the *Ericaceae* family were noted, which is a noteworthy research direction. There is also limited data across different cell model systems with respect to mechanisms of action on the cytoskeletal network. It is also unknown which phytochemical(s) are responsible for mediating alterations in the cytoskeleton in the CPAC and blueberry capsule extracts. Similar to *Ericaceae*, there are little to no mechanistic insights into cytoskeletal alterations induced by either purified phytochemicals or mixtures from the *Rosaceae* family, which should be considered a future research direction. Similar to the *Ericaceae* family, it is unknown which phytochemical(s) are responsible for mediating alterations in the cytoskeleton in the WS1442, RBE, SC, PA, DIE, RIE, PY, and Se-PFPs mixtures, although prepared from a rich diversity of plant species from this family.

### 6.2. Protein Trafficking Dynamics—Future Perspectives and Gaps in Knowledge

Regardless of the agent under investigation, with the exception of one study investigating the trafficking of sucrase-isomaltase to the BBM [68], the majority of the studies focused on expression profiling of key molecules in the ER stress pathway or in signaling events that were involved in modulating translocation of a cytoplasmically localized protein to the nuclear compartment. Therefore, this is a research area that could benefit from a broader focus using high-throughput screening strategies such as using tag-based fluorescence methods. This would enable an unbiased investigation of the detailed underlying mechanism of action of the phytochemical(s) in the relevant cell model system, specifically with efforts to study their effects on the alteration of organelle and trafficking dynamics.

As discussed earlier, while the role of cytoskeletal dynamics is primarily understood as being related to cellular structure, shape, and motility, these elements also play an intimate role in protein trafficking dynamics. For example, it is well known that actin assembly contributes to the process of clathrin-mediated endocytosis, which consequently affects signaling events [226]. Furthermore, the process underlying clathrin-mediated endocytosis has also been linked to a range of human diseases, including cancers, neurogenerative conditions, and even infectious diseases in which pathogens harness this mechanism to enter cells [227]. Such evidence corroborates the need to further explore these roles for these above-described plant metabolites, for which there is evidence of their ability to influence cytoskeletal integrity.

### 6.3. Signaling—Future Perspectives and Gaps in Knowledge

To a large extent, across the three plant families (*Cucurbitaceae*, *Ericaceae*, and *Rosaceae*), the effects of the phytochemicals (in extract or purified form) on signaling events were highly descriptive via assessment of changes in expression. However, there were a few studies that progressed to the next step in assessing whether the observed expression changes in the signaling molecule contributed to the functional outcomes (i.e., reduced cellular viability, reduced migration/invasion, decreased angiogenesis, and apoptosis). We propose that specific drugs targeting these pathways (i.e., PI3K, MAPK, EGFR, etc.), knockdown strategies (i.e., siRNA, shRNA, etc.), or overexpression approaches can be performed in combination with these plant phytochemicals. Likewise, only a few reports described efforts at molecular docking simulations to predict the affinity of their interactions with specific signaling mediators, such as EGFR. In some studies, changes in both phosphorylated and total signaling proteins were noted, and thus the dose or time of the agent utilized may be correlated with an apoptotic outcome, which includes dismantling and subsequent breakdown of the intracellular components; thus, interpretation of these findings of how the altered signaling mediator contributes to the mechanism by which the phytochemical alters the signaling event remains challenging.

Furthermore, although there were findings demonstrating the effect of various phytochemicals on EGFR kinase activity, future research directions could be focused on GPCRs, for which reports in this area are limiting. With respect to Rosaceae and Ericaceae, most of the studies utilized whole plant extracts or enriched fractions; in comparison, the use of purified phytochemicals was fewer, with, at times, opposing responses being elicited for more than one purified phytochemical from one plant species. Thus, further investigation into the effect and dosages of purified phytochemicals on signaling events *in vitro* and *in vivo* model systems, as well as it is *in vivo* bioactivity, is warranted. Finally, based on our search terms used in the initial literature screening for cucurbitacins from the Cucurbitaceae family, we uncovered findings focused predominantly on the JAK/STAT signaling cascade. This contrasts with the Ericaceae and Rosaceae families in which MAPK and PI3K/AKT/mTOR signaling events were predominantly uncovered. Therefore, towards the goal of uncovering whether the phytochemicals from these three different plant families uniquely target one or more of these pathways, one could apply an unbiased proteomic profiling approach such as Reverse Phase Protein Array (RPPA) or Mass-Spectrometric based approaches.

### 6.4. Limitations of This Study

Since our PubMed searches utilized specific terms, including the plant family, human, and a subset of specific molecules, this process of identifying articles may have eliminated other articles relevant to this area of research. For example, there may be specific species of plants within each family for which there may be information that was not identified in our broad search efforts. Furthermore, since there exists a large array of cytoskeletal regulatory factors and our searches did not focus on each one of these but only the major cytoskeletal elements, this would have also limited the articles identified. The use of the term “human” in all of our searches (to eliminate plant-based focused research) may also limit our findings by eliminating articles describing pre-clinical models. We also focused on specific signaling pathway elements (PI3K/AKT, JAK/STAT, and MAPK), although there is much crosstalk amongst these and with other transduction events for which we did not focus on. In addition, there were articles that intertwined cytoskeletal alterations, changes in translocation of key molecules, and signaling changes; however, we did not list these articles across multiple categories but focused on the major pathway of interest presented in the article.

With respect to the Cucurbitaceae family of plant metabolites, we focused on the cucurbitacins due to their high enrichment in this family. However, there are other relevant plant metabolites in the Cucurbitaceae family for which we did not pursue searches. Therefore, in this regard, this is another limitation of our study.

The methods utilized for the preparation of plant extracts and fractions may also be diverse across the various papers we reviewed, and this may thus affect the final biological outcomes. Future studies could focus on relating these specific methods (including doses used in cell systems and handling methods) to the biological outcomes, which may uncover differences due to differences in composition or loss in metabolite bioactivities. We also did not describe the side effects observed, if any, in any of the model organism studies. These would be highly relevant to translating such pre-clinical findings to the clinic.

### 6.5. Overall Future Perspectives

As discussed earlier, towards our review objective and for comparative purposes, the three plant families that were selected had at least 10 species in Hillsborough County, with two families associated with a high medicinal value (*Ericaceae* and *Rosaceae*) and one associated with a lesser medicinal value (*Cucurbitaceae*) in North America. Out of the plant genera in the *Cucurbitaceae* family that were uncovered in our searches, only two were described, namely *Cucumis* and *Thricosanthes*. With respect to the *Ericaceae* plant family, the *Rhododendron* and *Vaccinium* genera were reported in the analyzed studies. Within the *Rosaceae* family, the *Agrimonia*, *Aphanes*, *Aronia*, *Crataegus*, *Eriobotrya*, *Fragaria*, *Malus*, *Potentilla*, *Prunus*, *Pyracantha*, *Rosa*, *and Rubus* genera were analyzed within the framework of our search methods. Therefore, we propose that future research efforts could focus on the species from these specific genera and species (summarized in Table 1, Table 2 and Table 3) within our region of Hillsborough County to assess their medicinal value and underlying mechanisms of action on cytoskeletal organization, protein trafficking dynamics, and signaling events. With respect to our data mining of the Florida Plant Atlas, in which we identify the high abundance species across a large array of plant genera in Hillsborough county (Figure 1), we do not know of their relative abundance for each, which could be a future research direction.

Challenges have been encountered regarding the isolation of specific phytochemicals, including those from cranberry [160], and as such, current research has been restricted to the use of crude extracts with varying phytochemical contents, which may lead to variability in research findings. Wang and colleagues isolated specific flavonoids from cranberry extracts to the highest purity achieved thus far using high-performance column chromatography and subsequent characterization methods (i.e., HPLC and MS-approaches) [160]. Similar experimental approaches can be applied to the isolation of specific components from other plant extracts. However, there is still a need to critically evaluate published research studies and direct future research based on the feasibility of isolating metabolites from such plant species and/or applying them to synthesis platforms [3].

Overall, the majority of the studies analyzed herein were descriptive in nature within the cytoskeletal organization, protein trafficking dynamics, and signaling research topics. Further insight into uncovering the underlying mechanism of action can be pursued by using a combination of genetic approaches. Moreover, efforts to move forward using molecular docking simulations to investigate drug-protein interactions together with in silico experimental approaches would be valuable as a critical step in drug discovery [228,229].

## Figures and Tables

**Table 1:** *Cucurbitaceae* Family Plant Genera in Hillsborough County, Florida, United States.

CUCURBITACEAE of Florida				
Genera	Species	Status	Common Name	Hillsborough Wild Population?
***Cayaponia***	*Cayaponia americana* (Lam.) Cogn	Native	American Melonleaf	
	*Cayaponia quinqueloba* (Raf.) Shinners	Native	Fivelobe Melonleaf	
***Citrullus***	*Citrullus lanatus* (Thunb.) Matsum. & Nakai	Not Native	Watermelon	Yes
***Coccinia***	*Coccinia grandis* (L.) Voigt	Not Native	Ivy Gourd	Yes
***Cucumis***	*Cucumis anguria* L.	Not Native	Gooseberry gourd	Yes
	*Cucumis melo* L.	Not Native	Cantaloupe	Yes
	*Cucumis metulifer* E.Meyer ex Naudin	Not Native	African horned cucumber	Pinellas
	*Cucumis sativus* L.	Not Native	Garden cucumber	Yes
***Cucurbita***	*Cucurbita foetidissima* Kunth	Not Native	Buffalo Gourd	
	*Cucurbita moschata* Duchesne	Not Native	Seminole Pumpkin	Yes
	*Cucurbita okeechobeensis* (Small) L.H.Bailey	Native, endangered	Okeechobee Gourd	
***Lagenaria***	*Lagenaria siceraria* (Molina) Standl.	Not Native	Bottle Gourd	Yes
***Luffa***	*Luffa aegyptiaca* Mill.	Not Native	Loofah	Yes
***Melothria***	*Melothria pendula* L.	Native	Creeping Cucumber	Yes
***Momordica***	*Momordica balsamina* L.	Not Native	Southern Balsampear	
	*Momordica charantia* L.	Not Native, Invasive II	Balsampear	Yes
***Sicyos***	*Sicyos angulatus* L.	Native	Oneseed burr cucumber	
	*Sicyos edulis* Jacq.	Not Native	Chayote	
***Trichosanthes***	*Trichosanthes cucumerina* L.	Not Native	Snakegourd	

**Table 2:** *Ericaceae* Family Plant Genera in Hillsborough County, Florida, United States.

ERICACEAE of Florida				
Genera	Species	Status	Common Name	Hillsborough Wild Population?
***Agarista***	*Agarista populifolia* (Lam.) Judd	Native	Florida Hobblebush; Pinestem	
***Bejaria***	*Bejaria racemosa* Vent.	Native	Tarflower	Yes
***Ceratiola***	*Ceratiola ericoides* Michx.	Native	Florida Rosemary; Sand Heath	Yes
***Chimaphila***	*Chimaphila maculata* (L.) Pursh	Native	Spotted Wintergreen; Striped Prince’s Pine	
***Epigaea***	*Epigaea repens* L.	Native; Endangered (State)	Trailing Arbutus	
***Eubotrys***	*Eubotrys racemosus* (L.) Nutt.	Native	Swamp Doghobble	
***Gaylussacia***	*Gaylussacia dumosa* (Andrews) A. Gray	Native	Dwarf Huckleberry	Yes
	*Gaylussacia frondosa* (L.) Torr. & A. Gray	Native	Blue Huckleberry	Yes
	*Gaylussacia mosieri* Small	Native	Woolly Huckleberry	
***Hypopitys***	*Hypopitys lanuginosa* (Michx.) Raf.	Native; Endangered (State)	Pinesap; False Beechdrops	
***Kalmia***	*Kalmia hirsuta* Walter	Native	Wicky; Hair Laurel	
	*Kalmia latifolia* L.	Native; Threatened (State)	Mountain Laurel	
***Leucothoe***	*Leucothoe axillaris* (Lam.) D.Don	Native	Coastal Doghobble	
	*Lyonia ferruginea* (Walter) Nutt.	Native	Rusty Staggerbush	
	*Lyonia fruticosa* (Michx.) G.S. Torr.	Native	Coastalplain Staggerbush	Yes
	*Lyonia ligustrina* (L.) DC. var. *foliosiflora* (Michx.) Fernald	Native	Maleberry	Yes
	*Lyonia lucida* (Lam.) K.Koch	Native	Fetterbush	Yes
	*Lyonia mariana* (L.) D.Don	Native	Piedmont Staggerbush	Yes
***Monotropa***	*Monotropa uniflora* L.	Native	Indianpipe	Yes
***Monotropsis***	*Monotropsis reynoldsiae* (A. Gray) A. Heller	Native; Endangered (State)	Pigmypipes	
***Oxydendrum***	*Oxydendrum arboreum* (L.) DC.	Native	Sourwood	
***Pieris***	*Pieris phyllyreifolia* (Hook.) DC.	Native	Fetterbush	
***Rhododendron***	*Rhododendron alabamense* Rehder	Native; Endangered (State)	Alabama Azalea	
	*Rhododendron austrinum* (Small) Rehder	Native; Endangered (State)	Florida Flame Azaela; Orange Azalea	
	*Rhododendron canescens* (Michx.) Sweet	Native	Sweet Pinxter Azaela; Mountain Azalea	
	*Rhododendron minus* Michaux var. *chapmanii* (A.Gray) W.H. Duncan & Pullen	Native; Endangered (State & National)	Chapmen’s Rhododendron	
	*Rhododendron viscosum* (L.) Torr.	Native	Swamp Azalea	Yes
***Vaccinium***	*Vaccinium arboreum* Marshall	Native	Sparkleberry; Farkleberry	Yes
	*Vaccinium corymbosum* L.	Native	Highbush Blueberry	Yes
	*Vaccinium darrowii* Camp	Native	Darrow’s Blueberry	Yes
	*Vaccinium myrsinites* Lam.	Native	Shiny Blueberry	Yes
	*Vaccinium stamineum* L.	Native	Deerberry	Yes

**Table 3:** *Rosaceae* Family Plant Genera in Hillsborough County, Florida, United States.

ROSACEAE of Florida				
Genera	Species	Status	Common Name	Hillsborough Wild Population?
***Agrimonia***	*Agrimonia incisa* Torr. & A. Gray	Native (Threatened)	Incised Agrimony	Yes
	*Agrimonia microcarpa* Wallr.	Native	Smallfruit Agrimony	Yes
***Amelanchier***	*Amelanchier arborea* (R.Michx.) Fernald	Native	Common Serviceberry	Y
***Aphanes***	*Aphanes australis* Rydb.	Not Native	Slender Parsley Peirt	Y
***Aronia***	*Aronia arbutifolia* (L.) Pers.	Native	Red Chokeberry	Yes
***Crataegus***	*Crataegus aestivalis* (Walter) Torr. & A. Gray	Native	May Haw	Y
	*Crataegus crus-galli* L.	Native	Cockspur Hawthorn	Pinellas
	*Crataegus flava* Aiton	Native	Yellowleaf Hawthorn	Y
	*Crataegus marshallii* Eggl.	Native	Parsley Hawthorn	
	*Crataegus michauxii* Pers.	Native	Michaux’s Hawthorn	Yes
	*Crataegus opaca* Hook. & Arn.	Native	Riverflat Hawthorn	Yes
	*Crataegus phaenopyrum* (L.f.) Medik.	Native	Washington Hawthorn	
	*Crataegus spathulata* Michx.	Native	Littlehip Hawthorn	
	*Crataegus uniflora* Münchh.	Native	Dwarf Hawthorn	
	*Crataegus viridis* L.	Native	Green Hawthorn	
***Eriobotryto***	*Eriobotrya japonica* (Thunb.) Lindl.	Not Native	Loquat	Y
***Fragaria***	*Fragaria virginiana* Duchesne	Native	Virginia Strawberry	
***Malus***	*Malus angustifolia* (Aiton) Michx.	Native	Southern Crabapple	
***Physocarpus***	*Physocarpus opulifolius* (L.) Maxim.	Native	Common Ninebark	
***Potentilla***	*Potentilla indica* (Andrews) T. Wolf	Not Native	Indian Strawberry	Yes
	*Potentilla recta* L.	Not Native	Sulphur Cinquefoil	
	*Potentilla reptans* L.	Not Native	Creeping Cinquefoil	
	*Potentilla simplex* Michx.	Native	Common Cinquefoil	
***Prunus***	*Prunus americana* Marshall	Native	American Plum	
	*Prunus angustifolia* Marshall	Native	Chicksaw Plum	Yes
	*Prunus caroliniana* (Mill.) Aiton	Native	Carolina Laurelcherry	Yes
	*Prunus geniculata* R.M. Harper	Native	Scrub Palm	
	*Prunus myrtifolia* (L.) Urb.	Native	West Indian Cherry	
	*Prunus persica* (L.) Batsch	Not Native	Peach	
	*Prunus serotina* Ehrh.	Native	Black Cherry	Yes
	*Prunus subhirtella* Miq.	Not Native	Winter-Flowering Cheery	
	*Prunus umbellata* Elliott	Native	Flatwoods Plum	Yes
***Pyracantha***	*Pyracantha fortuneana* (Maxim.) H.L.Li	Not Native	Chinese Firethorn	
	*Pyracantha koidzumii* (Hayata) Rehder	Not Native	Formose Firethorn	Yes
***Pyrus***	*Pyrus calleryana* Decne.	Not Native	Callery Pear	
	*Pyrus communis* L.	Not Native	Common Pear	
***Rosa***	*Rosa bracteata* J.C. Wendl.	Not Native	Macartney Rose	
	*Rosa carolina* L.	Native	Carolina Rose	
	*Rosa laevigata* Michx.	Not Native	Cherokee Rose	Yes
	*Rosa lucieae* Franch. & Rochsebr. ex Crép.	Not Native	Memorial Rose	
	*Rosa multiflora* Thunb.	Not Native	Multiflora Rose	
	*Rosa palustris* Marshall	Native	Swamp Rose	Yes
	*Rosa setigera* Michx.	Native	Climbing Rose	
***Rubus***	*Rubus cuneifolius* Pursh	Native	Sand Blackberry	Yes
	*Rubus flagellaris* Willd.	Native	Northern Dewberry	
	*Rubus niveus* Thunb.	Not Native	Snowpeaks Rasberry	
	*Rubus pensilvanicus* Poir.	Native	Sawtooth Blackberry	Yes
	*Rubus trivalis* Michx.	Native	Southern Dewberry	Yes

**Table 4:** Summary of Effects on the Cytoskeleton across the three plant families.

Effects on the Cytoskeleton		
*Cucurbitaceae*		
Plant Metabolite	Associated Disease Model	References
CuB	Blood Cancers	[20,21,22]
	Solid Tumors	[23,24,25,26,27,28]
CuE	Blood Cancers	[29]
	Solid Tumors	[30,31,32,33]
	Other (*In Vitro*)	[34]
CuI	Solid Tumors	[35]
CuIIa	Solid Tumors	[36]
DHCF	Solid Tumors	[37]
IsoD	Solid Tumors	[38]
***Ericaceae***		
**Plant Metabolite**	**Associated Disease Model**	**References**
Cranberry proanthocyanidin extract (CPAC) from *Vaccinium macrocarpon*	Solid Tumors	[39]
Extract from capsule-form of billberry	Solid Tumors	[40]
Pterostilbene	Solid Tumors	[41]
***Rosaceae***		
**Plant Metabolite**	**Associated Disease Model**	**References**
Extract derived from *Crataegus spp* hawthorn (WS1442)	Endothelial Permeability, Neovascularization	[42,43]
Red raspberry extract (RBE)	Hepatic Fibrosis	[44]
Stem and cortex extracts from *Sorbus commixta* Hedl (SC)	Solid Tumors	[45]
Extracts from *Crataegus* berries, leaves, and flowers from 6 species	Solid Tumors	[46]
Extract from *Pygeum africanum* (PA)	Solid Tumors	[47]
Raspberry extract from *Rubus idaeus* L (RIE)	Solid Tumors	[48]
Leaf extracts from *Duchesna indica* (DIE)	Solid Tumors	[49]
Selenium-enriched polysaccharides from *Pyracantha fortuneana* (Se-PFPs)	Solid Tumors	[50]
Extracts from roots from *Sanguisorba officinalis* L (DY)	Solid Tumors	[51]
Aruncin B	Blood Cancers	[52]
Phloretin	Solid Tumors	[53]
Ellagic acid (EA)	Solid Tumors	[54]
Urolithin A (UA)	Solid Tumors	
Protocatechuic acid (PCA)	Solid Tumors	
Kaempferol	Solid Tumors	[55]

**Table 5:** Summary of Effects on the Protein Trafficking Dynamics across the three plant families.

Effects on Protein Trafficking Dynamics		
*Cucurbitaceae*		
Plant Metabolite	Associated Disease Model	References
CuB	Solid Tumors	[58]
CuE	Rheumatoid Arthritis	[59]
***Ericaceae***		
**Plant Metabolite**	**Associated Disease Model**	**References**
Blueberry powder	Solid Tumors	[60]
*Rhododendron luteum* extract (RLE)	Solid Tumors	[61]
(Poly)phenol-digested metabolites from leaves of *Arbutus unedo* (LPDMs)	Neurodegenerative Disease	[62]
Malvidin-3-glucoside (Mv-3-Gc) and Malvidin-3-galactoside (Mv-3-Gal)	Inflammation	[63]
***Rosaceae***		
**Plant Metabolite**	**Associated Disease Model**	**References**
Raspberry seed powder (RSF)	Obesity	[64]
Raspberry ketone (RK)	Obesity	[65]
Saskatoon berry powder (SBp)	Obesity	[66]
Strawberry tree honey from *Arubutus unedo* L (STH)	Solid Tumors	[67]
Methanol extract from *Rosa canina* (RCME)	Inflammation	[68]
Polyphenol extract from pulp of *Malus micromalus Makino* (MMPE)	Heavy Metal Toxicity	[69]
Saskatoon berry (SB)	Cardiovascular Disease	[70]
Cyanidin-3-galactoside (C3Ga)	Cardiovascular Disease	
Cyanidin-3-glucoside (C3G)	Cardiovascular Disease	
Asiatic acid (AAPC)	Liver Disease	[71]

## Data Availability

Data is contained within the article and Supplementary Materials.

## Supplementary Materials

The following supporting information can be downloaded at: https://www.mdpi.com/article/10.3390/ph15111380/s1, Table S1: PubMed Search Terms and Results for the *Cucurbitaceae* Family; Table S2: PubMed Search Terms and Results for the *Ericaceae* Family; Table S3: PubMed Search Terms and Results for the *Rosaceae* Family; Supplementary File S1: *Cucurbitaceae* Plant Family Metabolites used as purified agents or extracts; Supplementary File S2: *Ericaceae* Plant Family Metabolites used as purified agents or extracts; Supplementary File S3: *Rosaceae* Plant Family Metabolites used as purified agents or extracts; Supplementary File S4: Effects of *Cucurbitaceae*, *Ericaceae*, and *Rosaceae* plant family metabolites in human diseases.

## References

[1] Fabricant D.S., Farnsworth N.R. (2001). The value of plants used in traditional medicine for drug discovery. Environ. Health Perspect..

[2] Dhyani P., Quispe C., Sharma E., Bahukhandi A., Sati P., Attri D.C., Szopa A., Sharifi-Rad J., Docea A.O., Mardare I. (2022). Anticancer potential of alkaloids: A key emphasis to colchicine, vinblastine, vincristine, vindesine, vinorelbine and vincamine. Cancer Cell Int..

[3] Zhang J., Hansen L.G., Gudich O., Viehrig K., Lassen L.M.M., Schrubbers L., Adhikari K.B., Rubaszka P., Carrasquer-Alvarez E., Chen L. (2022). A microbial supply chain for production of the anti-cancer drug vinblastine. Nature.

[4] Da Silva T.C., da Silva J.M., Ramos M.A. (2018). What Factors Guide the Selection of Medicinal Plants in a Local Pharmacopoeia? A Case Study in a Rural Community from a Historically Transformed Atlantic Forest Landscape. Evid. Based Complement. Alternat. Med..

[5] Kew RBG (2017). State of the World’s Plants 2017. Royal Botanic Gardens, Kew. https://stateoftheworldsplants.org/.

[6] Moerman D.E. (1996). An analysis of the food plants and drug plants of native North America. J. Ethnopharmacol..

[7] Ford J., Gaoue O.G. (2017). Alkaloid-poor plant families, Poaceae and Cyperaceae, are over-utilized for medicine in Hawaiian pharmacopoeia. Econ. Bot..

[8] Phumthum M., Balslev H., Barfod A.S. (2019). Important Medicinal Plant Families in Thailand. Front Pharmacol..

[9] Wunderlin R.P., Hansen B.F., Franck A.R., Essig F.B. (2022). Atlas of Florida Plants. Institute for Systematic Botany, University of South Florida, Tampa. http://florida.plantatlas.usf.edu/.

[10] Rajasree R.S., Sibi P.I., Francis F., William H. (2016). Phytochemicals of Cucurbitaceae Family—A Review. Int. J. Pharmacogn. Phytochem. Res..

[11] Alghasham A.A. (2013). Cucurbitacins—A promising target for cancer therapy. Int. J. Health Sci..

[12] Kaushik U., Aeri V., Mir S.R. (2015). Cucurbitacins—An insight into medicinal leads from nature. Pharmacogn. Rev..

[13] Lyrene P.M., Sherman W.B. (1979). The Rabbiteye Blueberry Industry in Florida—1887 to 1930—With notes on the Current Status of Abandoned Plantations. Econ. Bot..

[14] Tundis R., Tenuta M.C., Loizzo M.R., Bonesi M., Finetti F., Trabalzini L., Deguin B. (2021). Vaccinium Species (Ericaceae): From Chemical Composition to Bio-Functional Activities. Appl. Sci..

[15] Wang X., Jiang R., Liu Z., Liu W., Xie M., Wei S., She G. (2014). Phytochemicals and biological activities of poisonous genera of Ericaceae in China. Nat. Prod. Commun..

[16] Soundararajan P., Won S.Y., Kim J.S. (2019). Insight on Rosaceae Family with Genome Sequencing and Functional Genomics Perspective. Biomed. Res. Int..

[17] Burton-Freeman B.M., Sandhu A.K., Edirisinghe I. (2016). Red Raspberries and Their Bioactive Polyphenols: Cardiometabolic and Neuronal Health Links. Adv. Nutr..

[18] Aseervatham J. (2020). Cytoskeletal Remodeling in Cancer. Biology.

[19] Bayless K.J., Johnson G.A. (2011). Role of the Cytoskeleton in Formation and Maintenance of Angiogenic Sprouts. J. Vasc. Res..

[20] Haritunians T., Gueller S., Zhang L., Badr R., Yin D., Xing H., Fung M.C., Koeffler H.P. (2008). Cucurbitacin B induces differentiation, cell cycle arrest, and actin cytoskeletal alterations in myeloid leukemia cells. Leuk. Res..

[21] Zhu J.-S., Ouyang D.-Y., Shi Z.-J., Xu L.-H., Zhang Y.-T., He X.-H. (2012). Cucurbitacin B Induces Cell Cycle Arrest, Apoptosis and Autophagy Associated with G Actin Reduction and Persistent Activation of Cofilin in Jurkat Cells. Pharmacology.

[22] Ueno M., Kariya R., Sittithumcharee G., Okada S. (2021). Cucurbitacin B induces apoptosis of primary effusion lymphoma via disruption of cytoskeletal organization. Phytomedicine.

[23] Wakimoto N., Yin D., O’Kelly J., Haritunians T., Karlan B., Said J., Xing H., Koeffler H.P. (2008). Cucurbitacin B has a potent antiproliferative effect on breast cancer cells *in vitro* and *in vivo*. Cancer Sci..

[24] Duangmano S., Sae-Lim P., Suksamrarn A., Domann F.E., Patmasiriwat P. (2012). Cucurbitacin B inhibits human breast cancer cell proliferation through disruption of microtubule polymerization and nucleophosmin/B23 translocation. BMC Complement. Altern. Med..

[25] Liang J., Zhang X.L., Yuan J.W., Zhang H.R., Liu D., Hao J., Ji W., Wu X.Z., Chen D. (2019). Cucurbitacin B inhibits the migration and invasion of breast cancer cells by altering the biomechanical properties of cells. Phytother. Res..

[26] Kausar H., Munagala R., Bansal S.S., Aqil F., Vadhanam M.V., Gupta R.C. (2013). Cucurbitacin B potently suppresses non-small-cell lung cancer growth: Identification of intracellular thiols as critical targets. Cancer Lett..

[27] Yin D., Wakimoto N., Xing H., Lu D., Huynh T., Wang X., Black K.L., Koeffler H.P. (2008). Cucurbitacin B markedly inhibits growth and rapidly affects the cytoskeleton in glioblastoma multiforme. Int. J. Cancer.

[28] Zhang Y.T., Xu L.H., Lu Q., Liu K.P., Liu P.Y., Ji F., Liu X.M., Ouyang D.Y., He X.H. (2014). VASP activation via the Galpha13/RhoA/PKA pathway mediates cucurbitacin-B-induced actin aggregation and cofilin-actin rod formation. PLoS ONE.

[29] Nakashima S., Matsuda H., Kurume A., Oda Y., Nakamura S., Yamashita M., Yoshikawa M. (2010). Cucurbitacin E as a new inhibitor of cofilin phosphorylation in human leukemia U937 cells. Bioorg. Med. Chem. Lett..

[30] Duncan K.L., Duncan M.D., Alley M.C., Sausville E.A. (1996). Cucurbitacin E-induced disruption of the actin and vimentin cytoskeleton in prostate carcinoma cells. Biochem. Pharmacol..

[31] Zhang T., Li J., Dong Y., Zhai D., Lai L., Dai F., Deng H., Chen Y., Liu M., Yi Z. (2012). Cucurbitacin E inhibits breast tumor metastasis by suppressing cell migration and invasion. Breast Cancer Res. Treat..

[32] Ma G., Luo W., Lu J., Ma D.-L., Leung C.-H., Wang Y., Chen X. (2016). Cucurbitacin E induces caspase-dependent apoptosis and protective autophagy mediated by ROS in lung cancer cells. Chem. Biol. Interact..

[33] Song H., Wang Y., Li L., Sui H., Wang P., Wang F. (2018). Cucurbitacin E Inhibits Proliferation and Migration of Intestinal Epithelial Cells via Activating Cofilin. Front. Physiol..

[34] Sorensen P.M., Iacob R.E., Fritzsche M., Engen J.R., Brieher W.M., Charras G., Eggert U.S. (2012). The natural product cucurbitacin E inhibits depolymerization of actin filaments. ACS Chem. Biol..

[35] Sari-Hassoun M., Clement M.-J., Hamdi I., Bollot G., Bauvais C., Joshi V., Toma F., Burgo A., Cailleret M., Rosales-Hernández M.C. (2016). Cucurbitacin I elicits the formation of actin/phospho-myosin II co-aggregates by stimulation of the RhoA/ROCK pathway and inhibition of LIM-kinase. Biochem. Pharmacol..

[36] Boykin C., Zhang G., Chen Y.H., Zhang R.W., Fan X.E., Yang W.M., Lu Q. (2011). Cucurbitacin IIa: A novel class of anti-cancer drug inducing non-reversible actin aggregation and inhibiting survivin independent of JAK2/STAT3 phosphorylation. Br. J. Cancer.

[37] Ren S., Ouyang D.-Y., Saltis M., Xu L.-H., Zha Q.-B., Cai J.-Y., He X.-H. (2012). Anti-proliferative effect of 23,24-dihydrocucurbitacin F on human prostate cancer cells through induction of actin aggregation and cofilin-actin rod formation. Cancer Chemother. Pharmacol..

[38] Nakashima S., Oda Y., Morita M., Ohta A., Morikawa T., Matsuda H., Nakamura S. (2022). Analysis of Active Compounds Using Target Protein Cofilin―Cucurbitacins in Cytotoxic Plant *Bryonia cretica*. Toxins.

[39] Harmidy K., Tufenkji N., Gruenheid S. (2011). Perturbation of Host Cell Cytoskeleton by Cranberry Proanthocyanidins and Their Effect on Enteric Infections. PLoS ONE.

[40] Nguyen V., Tang J., Oroudjev E., Lee C.J., Marasigan C., Wilson L., Ayoub G. (2010). Cytotoxic Effects of Bilberry Extract on MCF7-GFP-Tubulin Breast Cancer Cells. J. Med. Food.

[41] Mak K.K., Wu A.T., Lee W.H., Chang T.C., Chiou J.F., Wang L.S., Wu C.H., Huang C.Y., Shieh Y.S., Chao T.Y. (2013). Pterostilbene, a bioactive component of blueberries, suppresses the generation of breast cancer stem cells within tumor microenvironment and metastasis via modulating NF-kappaB/microRNA 448 circuit. Mol. Nutr. Food Res..

[42] Bubik M.F., Willer E.A., Bihari P., Jurgenliemk G., Ammer H., Krombach F., Zahler S., Vollmar A.M., Furst R. (2012). A novel approach to prevent endothelial hyperpermeability: The Crataegus extract WS(R) 1442 targets the cAMP/Rap1 pathway. J. Mol. Cell Cardiol..

[43] Sousa M., Machado V., Costa R., Figueira M., Sepodes B., Barata P., Ribeiro L., Soares R. (2016). Red Raspberry Phenols Inhibit Angiogenesis: A Morphological and Subcellular Analysis Upon Human Endothelial Cells. J. Cell. Biochem..

[44] Wu T.-H., Wang P.-W., Lin T.-Y., Yang P.-M., Li W.-T., Yeh C.-T., Pan T.-L. (2021). Antioxidant properties of red raspberry extract alleviate hepatic fibrosis via inducing apoptosis and transdifferentiation of activated hepatic stellate cells. Biomed. Pharmacother..

[45] Park H., Park H., Chung T.-W., Choi H.-J., Jung Y.-S., Lee S.-O., Ha K.-T. (2017). Effect of Sorbus commixta on the invasion and migration of human hepatocellular carcinoma Hep3B cells. Int. J. Mol. Med..

[46] Żurek N., Karatsai O., Rędowicz M.J., Kapusta I.T. (2021). Polyphenolic Compounds of *Crataegus* Berry, Leaf, and Flower Extracts Affect Viability and Invasive Potential of Human Glioblastoma Cells. Molecules.

[47] Quiles M.T., Arbós M.A., Fraga A., de Torres I.M., Reventós J., Morote J. (2010). Antiproliferative and apoptotic effects of the herbal agent Pygeum africanum on cultured prostate stromal cells from patients with benign prostatic hyperplasia (BPH). Prostate.

[48] Hsieh Y.S., Chu S.C., Hsu L.S., Chen K.S., Lai M.T., Yeh C.H., Chen P.N. (2013). Rubus idaeus L. reverses epithelial-to-mesenchymal transition and suppresses cell invasion and protease activities by targeting ERK1/2 and FAK pathways in human lung cancer cells. Food Chem. Toxicol..

[49] Chen P.N., Yang S.F., Yu C.C., Lin C.Y., Huang S.H., Chu S.C., Hsieh Y.S. (2017). Duchesnea indica extract suppresses the migration of human lung adenocarcinoma cells by inhibiting epithelial-mesenchymal transition. Environ. Toxicol..

[50] Sun Q., Dong M., Wang Z., Wang C., Sheng D., Li Z., Huang D., Yuan C. (2016). Selenium-enriched polysaccharides from *Pyracantha fortuneana* (Se-PFPs) inhibit the growth and invasive potential of ovarian cancer cells through inhibiting beta-catenin signaling. Oncotarget.

[51] Zhang W., Peng C., Yan J., Chen P., Jiang C., Sang S., Yuan Y., Hong Y., Yao M. (2022). *Sanguisorba officinalis* L. suppresses 5-fluorouracil-sensitive and-resistant colorectal cancer growth and metastasis via inhibition of the Wnt/beta-catenin pathway. Phytomedicine.

[52] Han C.R., Jun D.Y., Woo H.J., Jeong S.-Y., Woo M.-H., Kim Y.H. (2012). Induction of microtubule-damage, mitotic arrest, Bcl-2 phosphorylation, Bak activation, and mitochondria-dependent caspase cascade is involved in human Jurkat T-cell apoptosis by aruncin B from *Aruncus dioicus* var. kamtschaticus. Bioorg. Med. Chem. Lett..

[53] Wu K.-H., Ho C.-T., Chen Z.-F., Chen L.-C., Whang-Peng J., Lin T.-N., Ho Y.-S. (2018). The apple polyphenol phloretin inhibits breast cancer cell migration and proliferation via inhibition of signals by type 2 glucose transporter. J. Food Drug Anal..

[54] Eskra J.N., Schlicht M.J., Bosland M.C. (2019). Effects of Black Raspberries and Their Ellagic Acid and Anthocyanin Constituents on Taxane Chemotherapy of Castration-Resistant Prostate Cancer Cells. Sci. Rep..

[55] Tang H., Yang L., Wu L., Wang H., Chen K., Wu H., Li Y. (2021). Kaempferol, the melanogenic component of Sanguisorba officinalis, enhances dendricity and melanosome maturation/transport in melanocytes. J. Pharmacol. Sci..

[56] Tejeda-Muñoz N., Mei K.-C., Sheladiya P., Monka J. (2022). Targeting Membrane Trafficking as a Strategy for Cancer Treatment. Vaccines.

[57] Lippincott-Schwartz J., Roberts T.H., Hirschberg K. (2000). Secretory Protein Trafficking and Organelle Dynamics in Living Cells. Annu. Rev. Cell Dev. Biol..

[58] Dakeng S., Duangmano S., Jiratchariyakul W., U-Pratya Y., Bogler O., Patmasiriwat P. (2012). Inhibition of Wnt signaling by cucurbitacin B in breast cancer cells: Reduction of Wnt-associated proteins and reduced translocation of galectin-3-mediated beta-catenin to the nucleus. J. Cell Biochem..

[59] Jia Q., Cheng W., Yue Y., Hu Y., Zhang J., Pan X., Xu Z., Zhang P. (2015). Cucurbitacin E inhibits TNF-alpha-induced inflammatory cytokine production in human synoviocyte MH7A cells via suppression of PI3K/Akt/NF-kappaB pathways. Int. Immunopharmacol..

[60] Baba A.B., Kowshik J., Krishnaraj J., Sophia J., Dixit M., Nagini S. (2016). Blueberry inhibits invasion and angiogenesis in 7,12-dimethylbenz[a]anthracene (DMBA)-induced oral squamous cell carcinogenesis in hamsters via suppression of TGF-beta and NF-kappaB signaling pathways. J. Nutr. Biochem..

[61] Turan I., Demir S., Yaman S.O., Canbolat D., Mentese A., Aliyazicioglu Y. (2022). An Investigation of the Antiproliferative Effect of *Rhododendron luteum* Extract on Cervical Cancer (HeLa) Cells via Nrf2 Signaling Pathway. Nutr. Cancer.

[62] Macedo D., Jardim C.E.C.G., Figueira I., Almeida A.F., McDougall G.J., Stewart D., Yuste J.E., Tomás-Barberán F.A., Tenreiro S., Outeiro T.F. (2018). (Poly)phenol-digested metabolites modulate alpha-synuclein toxicity by regulating proteostasis. Sci. Rep..

[63] Huang W.-Y., Liu Y.-M., Wang J., Wang X.-N., Li C.Y. (2014). Anti-Inflammatory Effect of the Blueberry Anthocyanins Malvidin-3-Glucoside and Malvidin-3-Galactoside in Endothelial Cells. Molecules.

[64] Kang I., Espín J.C., Carr T.P., Tomás-Barberán F.A., Chung S. (2016). Raspberry seed flour attenuates high-sucrose diet-mediated hepatic stress and adipose tissue inflammation. J. Nutr. Biochem..

[65] Leu S.-Y., Chen Y.-C., Tsai Y.-C., Hung Y.-W., Hsu C.-H., Lee Y.M., Cheng P.-Y. (2017). Raspberry Ketone Reduced Lipid Accumulation in 3T3-L1 Cells and Ovariectomy-Induced Obesity in Wistar Rats by Regulating Autophagy Mechanisms. J. Agric. Food Chem..

[66] Zhao R., Xiang B., Dolinsky V.W., Xia M., Shen G.X. (2021). Saskatoon berry powder reduces hepatic steatosis and insulin resistance in high fat-high sucrose diet-induced obese mice. J. Nutr. Biochem..

[67] Afrin S., Giampieri F., Cianciosi D., Alvarez-Suarez J.M., Bullon B., Amici A., Quiles J.L., Forbes-Hernández T.Y., Battino M. (2021). Strawberry tree honey in combination with 5-fluorouracil enhances chemosensitivity in human colon adenocarcinoma cells. Food Chem. Toxicol..

[68] Wanes D., Toutounji M., Sebai H., Rizk S., Naim H.Y. (2021). *Rosa canina* L. Can Restore Endoplasmic Reticulum Alterations, Protein Trafficking and Membrane Integrity in a Dextran Sulfate Sodium-Induced Inflammatory Bowel Disease Phenotype. Nutrients.

[69] Wang G., Tang J., Song Q., Yu Q., Yao C., Li P., Ding Y., Lin M., Cheng D. (2020). Malus micromalus Makino phenolic extract preserves hepatorenal function by regulating PKC-α signaling pathway and attenuating endoplasmic reticulum stress in lead (II) exposure mice. J. Inorg. Biochem..

[70] Zhao R., Xie X., Le K., Li W., Moghadasian M.H., Beta T., Shen G.X. (2015). Endoplasmic reticulum stress in diabetic mouse or glycated LDL-treated endothelial cells: Protective effect of Saskatoon berry powder and cyanidin glycans. J. Nutr. Biochem..

[71] Wang D., Lao L., Pang X., Qiao Q., Pang L., Feng Z., Bai F., Sun X., Lin X., Wei J. (2018). Asiatic acid from Potentilla chinensis alleviates non-alcoholic fatty liver by regulating endoplasmic reticulum stress and lipid metabolism. Int. Immunopharmacol..

[72] Golemis E.A., Ochs M.F., Pugacheva E.N. (2001). Signal transduction driving technology driving signal transduction: Factors in the design of targeted therapies. J. Cell Biochem..

[73] Wang W.D., Liu Y., Su Y., Xiong X.Z., Shang D., Xu J.J., Liu H.J. (2017). Antitumor and Apoptotic Effects of Cucurbitacin a in a-549 Lung Carcinoma Cells Is Mediated Via G2/M Cell Cycle Arrest and M-Tor/Pi3k/Akt Signalling Pathway. Afr. J. Tradit. Complement. Altern. Med..

[74] Liu J., Liu X., Ma W., Kou W., Li C., Zhao J. (2018). Anticancer activity of cucurbitacin-A in ovarian cancer cell line SKOV3 involves cell cycle arrest, apoptosis and inhibition of mTOR/PI3K/Akt signaling pathway. J. BUON Off. J. Balk. Union Oncol..

[75] Xiao Y., Yang Z., Wu Q.Q., Jiang X.H., Yuan Y., Chang W., Bian Z.Y., Zhu J.X., Tang Q.Z. (2017). Cucurbitacin B Protects Against Pressure Overload Induced Cardiac Hypertrophy. J. Cell Biochem..

[76] Shang Y., Guo X.X., Li W.W., Rao W., Chen M.L., Mu L.N., Li S.J. (2014). Cucurbitacin-B inhibits neuroblastoma cell proliferation through up-regulation of PTEN. Eur. Rev. Med. Pharmacol. Sci..

[77] Qin S., Li J., Si Y., He Z., Zhang T., Wang D., Liu X., Guo Y., Zhang L., Li S. (2018). Cucurbitacin B induces inhibitory effects via CIP2A/PP2A/Akt pathway in glioblastoma multiforme. Mol. Carcinog..

[78] Gupta P., Srivastava S.K. (2014). Inhibition of HER2-integrin signaling by Cucurbitacin B leads to *in vitro* and *in vivo* breast tumor growth suppression. Oncotarget.

[79] Cai F., Zhang L., Xiao X., Duan C., Huang Q., Fan C., Li J., Liu X., Li S., Liu Y. (2016). Cucurbitacin B reverses multidrug resistance by targeting CIP2A to reactivate protein phosphatase 2A in MCF-7/adriamycin cells. Oncol. Rep..

[80] Niu Y., Sun W., Lu J.-J., Ma D.-L., Leung C.-H., Pei L., Chen X. (2016). PTEN Activation by DNA Damage Induces Protective Autophagy in Response to Cucurbitacin B in Hepatocellular Carcinoma Cells. Oxidative Med. Cell Longev..

[81] Klungsaeng S., Kukongviriyapan V., Prawan A., Kongpetch S., Senggunprai L. (2019). Cucurbitacin B induces mitochondrial-mediated apoptosis pathway in cholangiocarcinoma cells via suppressing focal adhesion kinase signaling. Naunyn-Schmiedeberg’s Arch. Pharmacol..

[82] Zhou B., Zong S., Zhong W., Tian Y., Wang L., Zhang Q., Zhang R., Li L., Wang W., Zhao J. (2020). Correction: Interaction between laminin-5gamma2 and integrin beta1 promotes the tumor budding of colorectal cancer via the activation of Yes-associated proteins. Oncogene.

[83] Thoennissen N.H., Iwanski G.B., Doan N.B., Okamoto R., Lin P., Abbassi S., Song J.H., Yin D., Toh M., Xie W.D. (2009). Cucurbitacin B Induces Apoptosis by Inhibition of the *JAK/STAT* Pathway and Potentiates Antiproliferative Effects of Gemcitabine on Pancreatic Cancer Cells. Cancer Res..

[84] Zhang M., Sun C., Shan X., Yang X., Li-Ling J., Deng Y. (2010). Inhibition of Pancreatic Cancer Cell Growth by Cucurbitacin B Through Modulation of Signal Transducer and Activator of Transcription 3 Signaling. Pancreas.

[85] Iwanski G.B., Lee D.H., En-Gal S., Doan N.B., Castor B., Vogt M., Toh M., Bokemeyer C., Said J.W., Thoennissen N.H. (2010). Cucurbitacin B, a novel *in vivo* potentiator of gemcitabine with low toxicity in the treatment of pancreatic cancer. Br. J. Pharmacol..

[86] Zhang Z.R., Gao M.X., Yang K. (2017). Cucurbitacin B inhibits cell proliferation and induces apoptosis in human osteosarcoma cells via modulation of the JAK2/STAT3 and MAPK pathways. Exp. Ther. Med..

[87] Zhang H., Zhao B., Wei H., Zeng H., Sheng D., Zhang Y. (2022). Cucurbitacin B controls M2 macrophage polarization to suppresses metastasis via targeting JAK-2/STAT3 signalling pathway in colorectal cancer. J. Ethnopharmacol..

[88] Liu J.H., Li C., Cao L., Zhang C.H., Zhang Z.H. (2022). Cucurbitacin B regulates lung cancer cell proliferation and apoptosis via inhibiting the IL-6/STAT3 pathway through the lncRNA XIST/miR-let-7c axis. Pharm. Biol..

[89] Zhou X., Yang J., Wang Y., Li W., Li-Ling J., Deng Y., Zhang M. (2012). Cucurbitacin B inhibits 12-O-tetradecanoylphorbol 13-acetate-induced invasion and migration of human hepatoma cells through inactivating mitogen-activated protein kinase and PI3K/Akt signal transduction pathways. Hepatol. Res..

[90] Liu P., Xiang Y., Liu X., Zhang T., Yang R., Chen S., Xu L., Yu Q., Zhao H., Zhang L. (2019). Cucurbitacin B Induces the Lysosomal Degradation of EGFR and Suppresses the CIP2A/PP2A/Akt Signaling Axis in Gefitinib-Resistant Non-Small Cell Lung Cancer. Molecules.

[91] Aiswarya S.U.D., Vikas G., Haritha N.H., Liju V.B., Shabna A., Swetha M., Rayginia T.P., Keerthana C.K., Nath L.R., Reshma M.V. (2022). Purified and Characterized From the Rhizome of *Corallocarpus epigaeus* Exhibits Anti-Melanoma Potential. Front Oncol..

[92] Wu D., Wang Z., Lin M., Shang Y., Wang F., Zhou J., Zhang X., Luo X., Huang W. (2019). *In Vitro* and *In Vivo* Antitumor Activity of Cucurbitacin C, a Novel Natural Product From Cucumber. Front. Pharmacol..

[93] Zhang Y.Z., Wang C.F., Zhang L.F. (2018). Cucurbitacin D impedes gastric cancer cell survival via activation of the iNOS/NO and inhibition of the Akt signalling pathway. Oncol. Rep..

[94] Wang D., Shen M., Kitamura N., Sennari Y., Morita K., Tsukada J., Kanazawa T., Yoshida Y. (2021). Mitogen-activated protein kinases are involved in cucurbitacin D-induced antitumor effects on adult T-cell leukemia cells. Investig. New Drugs.

[95] Kim M.S., Lee K., Ku J.M., Choi Y.J., Mok K., Kim D., Cheon C., Ko S.G. (2020). Cucurbitacin D Induces G2/M Phase Arrest and Apoptosis via the ROS/p38 Pathway in Capan-1 Pancreatic Cancer Cell Line. Evid. Based Complement. Alternat. Med..

[96] Zhang L., Liang H., Xin Y. (2020). Cucurbitacin E inhibits esophageal carcinoma cell proliferation, migration, and invasion by suppressing Rac1 expression through PI3K/AKT/mTOR pathway. Anti-Cancer Drugs.

[97] Song H., Sui H., Zhang Q., Wang P., Wang F. (2020). Cucurbitacin E Induces Autophagy-Involved Apoptosis in Intestinal Epithelial Cells. Front. Physiol..

[98] Liu Y., Yang H., Guo Q., Liu T., Jiang Y., Zhao M., Zeng K., Tu P. (2020). Cucurbitacin E Inhibits Huh7 Hepatoma Carcinoma Cell Proliferation and Metastasis via Suppressing MAPKs and JAK/STAT3 Pathways. Molecules.

[99] Dong Y., Lu B., Zhang X., Zhang J., Lai L., Li D., Wu Y., Song Y., Luo J., Pang X. (2010). Cucurbitacin E, a tetracyclic triterpenes compound from Chinese medicine, inhibits tumor angiogenesis through VEGFR2-mediated Jak2-STAT3 signaling pathway. Carcinogenesis.

[100] Jing S.-Y., Wu Z.-D., Zhang T.-H., Zhang J., Wei Z.-Y. (2020). *In vitro* antitumor effect of cucurbitacin E on human lung cancer cell line and its molecular mechanism. Chin. J. Nat. Med..

[101] Kong Y., Chen J., Zhou Z., Xia H., Qiu M.H., Chen C. (2014). Cucurbitacin E induces cell cycle G2/M phase arrest and apoptosis in triple negative breast cancer. PLoS ONE.

[102] Zhu X., Huang H., Zhang J., Liu H., Hao L., Xiao M., Wu Y. (2018). The anticancer effects of Cucurbitacin I inhibited cell growth of human non-small cell lung cancer through PI3K/AKT/p70S6K pathway. Mol. Med. Rep..

[103] Van Kester M.S., Out-Luiting J.J., von dem Borne P.A., Willemze R., Tensen C.P., Vermeer M.H. (2008). Cucurbitacin I inhibits Stat3 and induces apoptosis in Sezary cells. J. Investig. Dermatol..

[104] Blaskovich M.A., Sun J., Cantor A., Turkson J., Jove R., Sebti S.M. (2003). Discovery of JSI-124 (cucurbitacin I), a selective Janus kinase/signal transducer and activator of transcription 3 signaling pathway inhibitor with potent antitumor activity against human and murine cancer cells in mice. Cancer Res..

[105] Guo H., Kuang S., Song Q.-L., Liu M., Sun X.-X., Yu Q. (2018). Cucurbitacin I inhibits STAT3, but enhances STAT1 signaling in human cancer cells *in vitro* through disrupting actin filaments. Acta Pharmacol. Sin..

[106] Al-Harbi B., Aboussekhra A. (2021). Cucurbitacin I (JSI-124)-dependent inhibition of STAT3 permanently suppresses the pro-carcinogenic effects of active breast cancer-associated fibroblasts. Mol. Carcinog..

[107] Su Y., Li G., Zhang X., Gu J., Zhang C., Tian Z., Zhang J. (2008). JSI-124 inhibits glioblastoma multiforme cell proliferation through G(2)/M cell cycle arrest and apoptosis augment. Cancer Biol. Ther..

[108] Yuan G., Yan S.-F., Xue H., Zhang P., Sun J.-T., Li G. (2014). Cucurbitacin I Induces Protective Autophagy in Glioblastoma *in Vitro* and *in Vivo*. J. Biol. Chem..

[109] Chau M.N., Banerjee P.P. (2008). Development of a STAT3 reporter prostate cancer cell line for high throughput screening of STAT3 activators and inhibitors. Biochem. Biophys. Res. Commun..

[110] Lui V.W., Yau D.M., Wong E.Y., Ng Y.-K., Lau C.P.-K., Ho Y., Chan J.P., Hong B., Ho K., Cheung C.S. (2009). Cucurbitacin I elicits anoikis sensitization, inhibits cellular invasion and *in vivo* tumor formation ability of nasopharyngeal carcinoma cells. Carcinogenesis.

[111] Qi J., Xia G., Huang C.R., Wang J.X., Zhang J. (2015). JSI-124 (Cucurbitacin I) Inhibits Tumor Angiogenesis of Human Breast Cancer Through Reduction of STAT3 Phosphorylation. Am. J. Chin. Med..

[112] Ishdorj G., Johnston J.B., Gibson S.B. (2011). Cucurbitacin-I (JSI-124) activates the JNK/c-Jun signaling pathway independent of apoptosis and cell cycle arrest in B Leukemic Cells. BMC Cancer.

[113] Escandell J.M., Kaler P., Recio M.C., Sasazuki T., Shirasawa S., Augenlicht L., Ríos J.-L., Klampfer L. (2008). Activated kRas protects colon cancer cells from cucurbitacin-induced apoptosis: The role of p53 and p21. Biochem. Pharmacol..

[114] Ni Y., Wu S., Wang X., Zhu G., Chen X., Ding Y., Jiang W. (2018). Cucurbitacin I induces pro-death autophagy in A549 cells via the ERK-mTOR-STAT3 signaling pathway. J. Cell Biochem..

[115] Deng C., Zhang B., Zhang S., Duan C., Cao Y., Kang W., Yan H., Ding X., Zhou F., Wu L. (2016). Low nanomolar concentrations of Cucurbitacin-I induces G2/M phase arrest and apoptosis by perturbing redox homeostasis in gastric cancer cells *in vitro* and *in vivo*. Cell Death Dis..

[116] Wu Y., Chen H., Li R., Wang X., Li H., Xin J., Liu Z., Wu S., Jiang W., Zhu L. (2016). Cucurbitacin-I induces hypertrophy in H9c2 cardiomyoblasts through activation of autophagy via MEK/ERK1/2 signaling pathway. Toxicol. Lett..

[117] Sun J., Blaskovich M.A., Jove R., Livingston S.K., Coppola D. (2005). Sa Cucurbitacin Q: A selective STAT3 activation inhibitor with potent antitumor activity. Oncogene.

[118] Tannin-Spitz T., Grossman S., Dovrat S., Gottlieb H.E., Bergman M. (2007). Growth inhibitory activity of cucurbitacin glucosides isolated from Citrullus colocynthis on human breast cancer cells. Biochem. Pharmacol..

[119] Liu H., Wang H., Dong A., Huo X., Wang H., Wang J., Si J. (2022). The Inhibition of Gastric Cancer Cells’ Progression by 23,24-Dihydrocucurbitacin E through Disruption of the Ras/Raf/ERK/MMP9 Signaling Pathway. Molecules.

[120] Zhang J., Song Y., Liang Y., Zou H., Zuo P., Yan M., Jing S., Li T., Wang Y., Li D. (2019). Cucurbitacin IIa interferes with EGFR-MAPK signaling pathway leads to proliferation inhibition in A549 cells. Food Chem. Toxicol..

[121] Liang Y., Zhang T., Ren L., Jing S., Li Z., Zuo P., Li T., Wang Y., Zhang J., Wei Z. (2021). Cucurbitacin IIb induces apoptosis and cell cycle arrest through regulating EGFR/MAPK pathway. Environ. Toxicol. Pharmacol..

[122] Alhosin M., Leon-Gonzalez A.J., Dandache I., Lelay A., Rashid S.K., Kevers C., Pincemail J., Fornecker L.M., Mauvieux L., Herbrecht R. (2015). Bilberry extract (Antho 50) selectively induces redox-sensitive caspase 3-related apoptosis in chronic lymphocytic leukemia cells by targeting the Bcl-2/Bad pathway. Sci. Rep..

[123] Mansouri R.A., Percival S.S. (2020). Cranberry extract initiates intrinsic apoptosis in HL-60 cells by increasing BAD activity through inhibition of AKT phosphorylation. BMC Complement. Med. Ther..

[124] Vorsa N., Singh A.P., Lange T.S., Kim K.K., Brard L., Horan T., Moore R.G., Singh R.K. (2012). Purified cranberry proanthocyanidines (PAC-1A) cause pro-apoptotic signaling, ROS generation, cyclophosphamide retention and cytotoxicity in high-risk neuroblastoma cells. Int. J. Oncol..

[125] Kim K.K., Singh A.P., Singh R.K., DeMartino A., Brard L., Vorsa N., Lange T.S., Moore R.G. (2012). Anti-angiogenic activity of cranberry proanthocyanidins and cytotoxic properties in ovarian cancer cells. Int. J. Oncol..

[126] Déziel B.A., Patel K., Neto C., Gottschall-Pass K., Hurta R.A. (2010). Proanthocyanidins from the American Cranberry (Vaccinium macrocarpon) inhibit matrix metalloproteinase-2 and matrix metalloproteinase-9 activity in human prostate cancer cells via alterations in multiple cellular signalling pathways. J. Cell Biochem..

[127] Wu X., Song M., Cai X., Neto C., Tata A., Han Y., Wang Q., Tang Z., Xiao H. (2018). Chemopreventive Effects of Whole Cranberry (*Vaccinium macrocarpon*) on Colitis-Associated Colon Tumorigenesis. Mol. Nutr. Food Res..

[128] Lin Y., Li B., Zhao J., Wei L., Wang Y., Wang M., Dia V.P., Meng X. (2019). Combinatorial effect of blueberry extracts and oxaliplatin in human colon cancer cells. J. Cell Physiol..

[129] Adams L.S., Phung S., Yee N., Seeram N.P., Li L., Chen S. (2010). Blueberry Phytochemicals Inhibit Growth and Metastatic Potential of MDA-MB-231 Breast Cancer Cells through Modulation of the Phosphatidylinositol 3-Kinase Pathway. Cancer Res..

[130] Ranjani S., Kowshik J., Sophia J., Nivetha R., Baba A.B., Veeravarmal V., Joksic G., Rutqvist L.E., Nilsson R., Nagini S. (2020). Activation of PI3K/Akt/NF-kB Signaling Mediates Swedish Snus Induced Proliferation and Apoptosis Evasion in the Rat Forestomach: Modulation by Blueberry. Anticancer Agents Med. Chem..

[131] Tsakiroglou P., Weber J., Ashworth S., Del Bo’ C., Klimis-Zacas D. (2021). Angiogenesis is Differentially Modulated by Anthocyanin and Phenolic Acid Extracts from Wild Blueberry (*V. angustifolium*) Through PI3K Pathway. J. Med. Food.

[132] Bryl-Gorecka P., Sathanoori R., Arevstrom L., Landberg R., Bergh C., Evander M., Olde B., Laurell T., Frobert O., Erlinge D. (2020). Bilberry Supplementation after Myocardial Infarction Decreases Microvesicles in Blood and Affects Endothelial Vesiculation. Mol. Nutr. Food Res..

[133] Li N., Li J., Hao J., Zhang M., Yin J., Geng J., Wu T., Lyv X. (2019). Bilberry anthocyanin improves the serum cholesterol in aging perimenopausal rats *via* the estrogen receptor signaling pathway. Food Funct..

[134] Si X., Tian J., Shu C., Wang Y., Gong E., Zhang Y., Zhang W., Cui H., Li B. (2020). Serum Ceramide Reduction by Blueberry Anthocyanin-Rich Extract Alleviates Insulin Resistance in Hyperlipidemia Mice. J. Agric. Food Chem..

[135] Huang W., Yan Z., Li D., Ma Y., Zhou J., Sui Z. (2018). Antioxidant and Anti-Inflammatory Effects of Blueberry Anthocyanins on High Glucose-Induced Human Retinal Capillary Endothelial Cells. Oxidative Med. Cell Longev..

[136] Huang W.-Y., Wu H., Li D.-J., Song J.-F., Xiao Y.-D., Liu C.-Q., Zhou J.-Z., Sui Z.-Q. (2018). Protective Effects of Blueberry Anthocyanins against H_2_O_2_-Induced Oxidative Injuries in Human Retinal Pigment Epithelial Cells. J. Agric. Food Chem..

[137] Williams C., El Mohsen M.A., Vauzour D., Rendeiro C., Butler L.T., Ellis J.A., Whiteman M., Spencer J.P. (2008). Blueberry-induced changes in spatial working memory correlate with changes in hippocampal CREB phosphorylation and brain-derived neurotrophic factor (BDNF) levels. Free Radic. Biol. Med..

[138] Ichikawa T., Sugamoto K., Matsuura Y., Kunitake H., Shimoda K., Morishita K. (2022). Inhibition of adult T-cell leukemia cell proliferation by polymerized proanthocyanidin from blueberry leaves through JAK proteolysis. Cancer Sci..

[139] Baba A.B., Nivetha R., Chattopadhyay I., Nagini S. (2017). Blueberry and malvidin inhibit cell cycle progression and induce mitochondrial-mediated apoptosis by abrogating the JAK/STAT-3 signalling pathway. Food Chem. Toxicol..

[140] Park J.W., Lee H.S., Lim Y., Paik J.H., Kwon O.K., Kim J.H., Paryanto I., Yunianto P., Choi S., Oh S.R. (2018). Rhododendron album Blume extract inhibits TNF-alpha/IFN-gamma-induced chemokine production via blockade of NF-kappaB and JAK/STAT activation in human epidermal keratinocytes. Int. J. Mol. Med..

[141] Rooprai H.K., Christidou M., Murray S.A., Davies D., Selway R., Gullan R.W., Pilkington G.J. (2021). Inhibition of Invasion by Polyphenols from Citrus Fruit and Berries in Human Malignant Glioma Cells *In Vitro*. Anticancer Res..

[142] Kropat C., Betz M., Kulozik U., Leick S., Rehage H., Boettler U., Teller N., Marko D. (2013). Effect of Microformulation on the Bioactivity of an Anthocyanin-rich Bilberry Pomace Extract (*Vaccinium myrtillus* L.) *in Vitro*. J. Agric. Food Chem..

[143] Teller N., Thiele W., Marczylo T.H., Gescher A.J., Boettler U., Sleeman J., Marko D. (2009). Suppression of the Kinase Activity of Receptor Tyrosine Kinases by Anthocyanin-Rich Mixtures Extracted from Bilberries and Grapes. J. Agric. Food Chem..

[144] Vuong T., Mallet J.-F., Ouzounova M., Rahbar S., Hernandez-Vargas H., Herceg Z., Matar C. (2016). Role of a polyphenol-enriched preparation on chemoprevention of mammary carcinoma through cancer stem cells and inflammatory pathways modulation. J. Transl. Med..

[145] Wang S.Y., Feng R., Bowman L., Penhallegon R., Ding M., Lu Y. (2005). Antioxidant activity in lingonberries (*Vaccinium vitis-idaea* L.) and its inhibitory effect on activator protein-1, nuclear factor-kappaB, and mitogen-activated protein kinases activation. J. Agric. Food Chem..

[146] Kim H.N., Baek J.K., Park S.B., Kim J.D., Son H.J., Park G.H., Eo H.J., Park J.H., Jung H.S., Jeong J.B. (2019). Anti-inflammatory effect of Vaccinium oldhamii stems through inhibition of NF-kappaB and MAPK/ATF2 signaling activation in LPS-stimulated RAW264.7 cells. BMC Complement. Altern. Med..

[147] Shu C., Tian J., Si X., Xie X., Li B., Li D. (2022). Blueberry anthocyanin extracts protect against Helicobacter pylori-induced peptic epithelium injuries both *in vitro* and *in vivo*: The key role of MAPK/NF-kappaB pathway. Eur. J. Nutr..

[148] Bae J.-Y., Lim S.S., Kim S.J., Choi J.-S., Park J., Ju S.M., Han S.J., Kang I.-J., Kang Y.-H. (2009). Bog blueberry anthocyanins alleviate photoaging in ultraviolet-B irradiation-induced human dermal fibroblasts. Mol. Nutr. Food Res..

[149] Tipton D.A., Carter T.B., Dabbous M. (2014). Inhibition of interleukin 1beta-stimulated interleukin-6 production by cranberry components in human gingival epithelial cells: Effects on nuclear factor kappaB and activator protein 1 activation pathways. J. Periodontal. Res..

[150] Xie C., Kang J., Chen J.R., Nagarajan S., Badger T.M., Wu X. (2011). Phenolic acids are *in vivo* atheroprotective compounds appearing in the serum of rats after blueberry consumption. J. Agric. Food Chem..

[151] Joseph J.A., Shukitt-Hale B., Brewer G.J., Weikel K.A., Kalt W., Fisher D.R. (2010). Differential Protection among Fractionated Blueberry Polyphenolic Families against DA-, Aβ42- and LPS-Induced Decrements in Ca^2+^ Buffering in Primary Hippocampal Cells. J. Agric. Food Chem..

[152] Zhu Y., Bickford P.C., Sanberg P., Giunta B., Tan J. (2008). Blueberry opposes beta-amyloid peptide-induced microglial activation via inhibition of p44/42 mitogen-activation protein kinase. Rejuvenation Res..

[153] Tolba M.F., Abdel-Rahman S.Z. (2015). Pterostilbine, an active component of blueberries, sensitizes colon cancer cells to 5-fluorouracil cytotoxicity. Sci. Rep..

[154] Chen G., Xu Z., Chang G., Hou J., Hu L., Zhang Y., Yu D., Li B., Chang S., Xie Y. (2017). The blueberry component pterostilbene has potent anti-myeloma activity in bortezomib-resistant cells. Oncol. Rep..

[155] Way T.D., Tsai S.J., Wang C.M., Jhan Y.L., Ho C.T., Chou C.H. (2015). Cinnamtannin D1 from Rhododendron formosanum Induces Autophagy via the Inhibition of Akt/mTOR and Activation of ERK1/2 in Non-Small-Cell Lung Carcinoma Cells. J. Agric. Food Chem..

[156] Wu Y., Shi Q., Zhu P., Ma H., Cui S., Li J., Hou A., Li J. (2021). Rhodomeroterpene alleviates macrophage infiltration and the inflammatory response in renal tissue to improve acute kidney injury. FASEB J..

[157] Ku S.-K., Zhou W., Lee W., Han M.-S., Na M., Bae J.-S. (2015). Anti-Inflammatory Effects of Hyperoside in Human Endothelial Cells and in Mice. Inflammation.

[158] Huang W., Hutabarat R.P., Chai Z., Zheng T., Zhang W., Li D. (2020). Antioxidant Blueberry Anthocyanins Induce Vasodilation via PI3K/Akt Signaling Pathway in High-Glucose-Induced Human Umbilical Vein Endothelial Cells. Int. J. Mol. Sci..

[159] Tian J.-L., Liao X.-J., Wang Y.-H., Si X., Shu C., Gong E.-S., Xie X., Ran X.-L., Li B. (2019). Identification of Cyanidin-3-arabinoside Extracted from Blueberry as a Selective Protein Tyrosine Phosphatase 1B Inhibitor. J. Agric. Food Chem..

[160] Wang Y., Han A., Chen E., Singh R.K., Chichester C.O., Moore R.G., Singh A.P., Vorsa N. (2015). The cranberry flavonoids PAC DP-9 and quercetin aglycone induce cytotoxicity and cell cycle arrest and increase cisplatin sensitivity in ovarian cancer cells. Int. J. Oncol..

[161] Wang Y.H., Lin J., Tian J., Si X., Jiao X., Zhang W., Gong E., Li B. (2019). Blueberry Malvidin-3-galactoside Suppresses Hepatocellular Carcinoma by Regulating Apoptosis, Proliferation, and Metastasis Pathways *In Vivo* and *In Vitro*. J. Agric. Food Chem..

[162] Zhang X., Sun J., Xin W., Li Y., Ni L., Ma X., Zhang D., Zhang D., Zhang T., Du G. (2015). Anti-inflammation effect of methyl salicylate 2-O-beta-D-lactoside on adjuvant induced-arthritis rats and lipopolysaccharide (LPS)-treated murine macrophages RAW264.7 cells. Int. Immunopharmacol..

[163] Jeon Y.J., Kim B.H., Kim S., Oh I., Lee S., Shin J., Kim T.Y. (2013). Rhododendrin ameliorates skin inflammation through inhibition of NF-kappaB, MAPK, and PI3K/Akt signaling. Eur. J. Pharmacol..

[164] Wang K.-C., Liu Y.-C., El-Shazly M., Shih S.-P., Du Y.-C., Hsu Y.-M., Lin H.-Y., Chen Y.-C., Wu Y.-C., Yang S.-C. (2019). The Antioxidant from Ethanolic Extract of Rosa cymosa Fruits Activates Phosphatase and Tensin Homolog *In Vitro* and *In Vivo*: A New Insight on Its Antileukemic Effect. Int. J. Mol. Sci..

[165] Tan A.C., Kończak I., Ramzan I., Zabaras D., Sze D.M.-Y. (2011). Potential Antioxidant, Antiinflammatory, and Proapoptotic Anticancer Activities of Kakadu Plum and Illawarra Plum Polyphenolic Fractions. Nutr. Cancer.

[166] Chu S.-C., Hsieh Y.-S., Hsu L.-S., Chen K.-S., Chiang C.-C., Chen P.-N. (2014). *Rubus idaeus* L Inhibits Invasion Potential of Human A549 Lung Cancer Cells by Suppression Epithelial-to-Mesenchymal Transition and Akt Pathway *In Vitro* and Reduces Tumor Growth *In Vivo*. Integr. Cancer Ther..

[167] Shi N., Clinton S.K., Liu Z., Wang Y., Riedl K.M., Schwartz S.J., Zhang X., Pan Z., Chen T. (2015). Strawberry Phytochemicals Inhibit Azoxymethane/Dextran Sodium Sulfate-Induced Colorectal Carcinogenesis in Crj: CD-1 Mice. Nutrients.

[168] Zhang H., Liu J., Li G., Wei J., Chen H., Zhang C., Zhao J., Wang Y., Dang S., Li X. (2018). Fresh red raspberry phytochemicals suppress the growth of hepatocellular carcinoma cells by PTEN/AKT pathway. Int. J. Biochem. Cell Biol..

[169] Lim W.-C., Choi H.-K., Kim K.-T., Lim T.-G. (2020). Rose (*Rosa gallica*) Petal Extract Suppress Proliferation, Migration, and Invasion of Human Lung Adenocarcinoma A549 Cells through via the EGFR Signaling Pathway. Molecules.

[170] Li W., Cheng M., Zhang W., He R., Yang H. (2021). New Insights into the Mechanisms of Polyphenol from Plum Fruit Inducing Apoptosis in Human Lung Cancer A549 Cells Via PI3K/AKT/FOXO1 Pathway. Mater. Veg..

[171] Li C.X., Lin Z.X., Zhao X.H., Zuo W.F., Wang N., Zhang Z.Y., Chen X.S. (2021). Differential effects of phenolic extracts from red-fleshed apple peels and flesh induced G1 cell cycle arrest and apoptosis in human breast cancer MDA-MB-231 cells. J. Food Sci..

[172] Kim Y., Lee S.M., Kim J.-H. (2014). Unripe *Rubus coreanus* Miquel suppresses migration and invasion of human prostate cancer cells by reducing matrix metalloproteinase expression. Biosci. Biotechnol. Biochem..

[173] Liu Y., Li H., Zheng Z., Niu A., Liu S., Li W., Ren P., Liu Y., Inam M., Guan L. (2022). Rosa rugosa polysaccharide induces autophagy-mediated apoptosis in human cervical cancer cells via the PI3K/AKT/mTOR pathway. Int. J. Biol. Macromol..

[174] Lee J., Shin M.S., Kim M.O., Jang S., Oh S.W., Kang M., Jung K., Park Y.S., Lee J. (2016). Apple ethanol extract promotes proliferation of human adult stem cells, which involves the regenerative potential of stem cells. Nutr. Res..

[175] Medda R., Lyros O., Schmidt J.L., Jovanovic N., Nie L., Link B.J., Otterson M.F., Stoner G.D., Shaker R., Rafiee P. (2015). Anti inflammatory and anti angiogenic effect of black raspberry extract on human esophageal and intestinal microvascular endothelial cells. Microvasc. Res..

[176] Jian T., Chen J., Ding X., Lv H., Li J., Wu Y., Ren B., Tong B., Zuo Y., Su K. (2020). Flavonoids isolated from loquat (*Eriobotrya japonica*) leaves inhibit oxidative stress and inflammation induced by cigarette smoke in COPD mice: The role of TRPV1 signaling pathways. Food Funct..

[177] Yu T., Lee Y.J., Jang H.-J., Kim A.R., Hong S., Kim T.W., Kim M.-Y., Lee J., Cho J.Y. (2011). Anti-inflammatory activity of Sorbus commixta water extract and its molecular inhibitory mechanism. J. Ethnopharmacol..

[178] Yamauchi Y., Okuyama T., Ishii T., Okumura T., Ikeya Y., Nishizawa M. (2019). Sakuranetin downregulates inducible nitric oxide synthase expression by affecting interleukin-1 receptor and CCAAT/enhancer-binding protein beta. J. Nat. Med..

[179] Sung B., Hwang S.Y., Kim M.J., Kim M., Jeong J.W., Kim C.M., Chung H.Y., Kim N.D. (2015). Loquat leaf extract enhances myogenic differentiation, improves muscle function and attenuates muscle loss in aged rats. Int. J. Mol. Med..

[180] Makanae Y., Ato S., Kido K., Fujita S. (2019). Dietary *Aronia melanocarpa* extract enhances mTORC1 signaling, but has no effect on protein synthesis and protein breakdown-related signaling, in response to resistance exercise in rat skeletal muscle. J. Int. Soc. Sports Nutr..

[181] Cheng J., Zhao L., Liu D., Shen R., Bai D. (2022). *Potentilla anserine* L. polysaccharide protects against cadmium-induced neurotoxicity. Environ. Toxicol. Pharmacol..

[182] Qin B., Anderson R.A. (2012). An extract of chokeberry attenuates weight gain and modulates insulin, adipogenic and inflammatory signalling pathways in epididymal adipose tissue of rats fed a fructose-rich diet. Br. J. Nutr..

[183] Elyasiyan U., Nudel A., Skalka N., Rozenberg K., Drori E., Oppenheimer R., Kerem Z., Rosenzweig T. (2017). Anti-diabetic activity of aerial parts of Sarcopoterium spinosum. BMC Complement. Altern. Med..

[184] Liu S., Yu J., Fu M., Wang X., Chang X. (2021). Regulatory effects of hawthorn polyphenols on hyperglycemic, inflammatory, insulin resistance responses, and alleviation of aortic injury in type 2 diabetic rats. Food Res. Int..

[185] Mladenova S.G., Vasileva L.V., Savova M.S., Marchev A.S., Tews D., Wabitsch M., Ferrante C., Orlando G., Georgiev M.I. (2021). Anti-Adipogenic Effect of Alchemilla monticola is Mediated Via PI3K/AKT Signaling Inhibition in Human Adipocytes. Front. Pharmacol..

[186] Kim D.-H., Lee J.-Y., Kim Y.-J., Kim H.-J., Park W. (2020). Rubi Fructus Water Extract Alleviates LPS-Stimulated Macrophage Activation via an ER Stress-Induced Calcium/CHOP Signaling Pathway. Nutrients.

[187] Yang J.H., Yoo J.M., Cho W.K., Ma J.Y. (2016). Anti-inflammatory effects of Sanguisorbae Radix water extract on the suppression of mast cell degranulation and STAT-1/Jak-2 activation in BMMCs and HaCaT keratinocytes. BMC Complement. Altern. Med..

[188] Mace T.A., King S.A., Ameen Z., Elnaggar O., Young G., Riedl K.M., Schwartz S.J., Clinton S.K., Knobloch T.J., Weghorst C.M. (2014). Bioactive compounds or metabolites from black raspberries modulate T lymphocyte proliferation, myeloid cell differentiation and Jak/STAT signaling. Cancer Immunol. Immunother..

[189] Wang S.Y., Feng R., Lu Y., Bowman L., Ding M. (2005). Inhibitory effect on activator protein-1, nuclear factor-kappaB, and cell transformation by extracts of strawberries (Fragaria x ananassa Duch). J. Agric. Food Chem..

[190] Lee S.-H., Cho K.-J., Choi W.-S., Lee H.K., Yoon E.K., Son M., Woo S.-U., Kweon M.-A., Heo J.-C. (2008). A fraction of methylene chloride from Geum japonicum Thunberg inhibits tumor metastatic and angiogenic potential. Oncol. Rep..

[191] Yu M.H., Im H.G., Lee S.G., Kim D.-I., Seo H.J., Lee I.-S. (2009). Inhibitory effect of immature plum on PMA-induced MMP-9 expression in human hepatocellular carcinoma. Nat. Prod. Res..

[192] Noratto G., Layosa M.A., Lage N.N., Atienza L., Ivanov I., Mertens-Talcott S.U., Chew B.P. (2020). Antitumor potential of dark sweet cherry sweet (*Prunus avium*) phenolics in suppressing xenograft tumor growth of MDA-MB-453 breast cancer cells. J. Nutr. Biochem..

[193] Layosa M.A.A., Lage N.N., Chew B.P., Atienza L., Mertens-Talcott S., Talcott S., Noratto G.D. (2021). Dark Sweet Cherry (*Prunus avium*) Phenolics Enriched in Anthocyanins Induced Apoptosis in MDA-MB-453 Breast Cancer Cells through MAPK-Dependent Signaling and Reduced Invasion via Akt and PLCgamma-1 Downregulation. Nutr. Cancer.

[194] Eom S.Y., Kim M. (2021). The inhibitory effect of *Agrimonia Pilosa* methanolic extract on matrix metalloproteinases in HT1080 cells. J. Food Biochem..

[195] Chojnacka K., Owczarek K., Caban M., Sosnowska D., Kajszczak D., Lewandowska U. (2022). Chemoprotective effects of Japanese quince (*Chaenomeles japonica* L.) phenol leaf extract on colon cancer cells through the modulation of extracellular signal-regulated kinases/AKT signaling pathway. J. Physiol. Pharmacol..

[196] Utsunomiya H., Takekoshi S., Gato N., Utatsu H., Motley E.D., Eguchi K., Fitzgerald T.G., Mifune M., Frank G.D., Eguchi S. (2002). Fruit-juice concentrate of Asian plum inhibits growth signals of vascular smooth muscle cells induced by angiotensin II. Life Sci..

[197] Castagnini C., Luceri C., Toti S., Bigagli E., Caderni G., Femia A.P., Giovannelli L., Lodovici M., Pitozzi V., Salvadori M. (2009). Reduction of colonic inflammation in HLA-B27 transgenic rats by feeding Marie Menard apples, rich in polyphenols. Br. J. Nutr..

[198] Bao M.J., Shen J., Jia Y.L., Li F.F., Ma W.J., Shen H.J., Shen L.L., Lin X.X., Zhang L.H., Dong X.W. (2013). Apple polyphenol protects against cigarette smoke-induced acute lung injury. Nutrition.

[199] Yun J.M., Im S.B., Roh M.K., Park S.H., Kwon H.A., Lee J.Y., Choi H.Y., Ham I.H., Kim Y.B., Lee J.M. (2014). Prunus yedoensis bark inhibits lipopolysaccharide-induced inflammatory cytokine synthesis by IkappaBalpha degradation and MAPK activation in macrophages. J. Med. Food.

[200] Nguyen Q.T.N., Fang M., Zhang M., Do N.Q., Kim M., Zheng S.D., Hwang E., Yi T.H. (2021). *Crataegus laevigata* Suppresses LPS-Induced Oxidative Stress during Inflammatory Response in Human Keratinocytes by Regulating the MAPKs/AP-1, NFkappaB, and NFAT Signaling Pathways. Molecules.

[201] Hwang D.H., Koh P.-O., Kang C., Kim E. (2021). Rosa davurica Pall. improves DNCB-induced atopic dermatitis in mice and regulated TNF-Alpa/IFN-gamma-induced skin inflammatory responses in HaCaT cells. Phytomedicine.

[202] Essafi-Benkhadir K., Refai A., Riahi I., Fattouch S., Karoui H., Essafi M. (2012). Quince (*Cydonia oblonga* Miller) peel polyphenols modulate LPS-induced inflammation in human THP-1-derived macrophages through NF-kappaB, p38MAPK and Akt inhibition. Biochem. Biophys. Res. Commun..

[203] Mi X.-J., Kim J.-K., Lee S., Moon S.-K., Kim Y.-J., Kim H. (2022). *In vitro* assessment of the anti-inflammatory and skin-moisturizing effects of *Filipendula palmata* (Pall.) Maxim. On human keratinocytes and identification of its bioactive phytochemicals. J. Ethnopharmacol..

[204] Kim S.-H., Kim H.-H., Choi P.H., Yoo J.-S., Jeon H., Chae B.-S., Park J.-S., Shin T.-Y. (2012). Ripe fruit of Rubus coreanus inhibits mast cell-mediated allergic inflammation. Int. J. Mol. Med..

[205] Liu M., Xu Y., Han X., Liang C., Yin L., Xu L., Qi Y., Zhao Y., Peng J., Sun C. (2014). Potent Effects of Flavonoid-Rich Extract from Rosa laevigata Michx Fruit against Hydrogen Peroxide-Induced Damage in PC12 Cells via Attenuation of Oxidative Stress, Inflammation and Apoptosis. Molecules.

[206] Lee K.M., Bang J., Kim B.-Y., Lee I.S., Han J.-S., Hwang B.Y., Jeon W.K. (2015). Fructus mume alleviates chronic cerebral hypoperfusion-induced white matter and hippocampal damage via inhibition of inflammation and downregulation of TLR4 and p38 MAPK signaling. BMC Complement. Altern. Med..

[207] Ma B., Wang J., Tong J., Zhou G., Chen Y., He J., Wang Y. (2016). Protective effects of *Chaenomeles thibetica* extract against carbon tetrachloride-induced damage via the MAPK/Nrf2 pathway. Food Funct..

[208] Xuan S.H., Park Y.M., Park S.H., Jeong H.J., Park S.N. (2018). Suppression of Ultraviolet B-mediated Matrix Metalloproteinase Generation by *Sorbus commixta* Twig Extract in Human Dermal Fibroblasts. Photochem. Photobiol..

[209] Li L., Hwang E., Ngo H.T.T., Lin P., Gao W., Liu Y., Yi T.H. (2018). Antiphotoaging Effect of *Prunus yeonesis* Blossom Extract via Inhibition of MAPK/AP-1 and Regulation of the TGF-betaI/Smad and Nrf2/ARE Signaling Pathways. Photochem. Photobiol..

[210] Gao W., Wang Y.-S., Hwang E., Lin P., Bae J., Seo S.A., Yan Z., Yi T.-H. (2018). *Rubus idaeus* L. (red raspberry) blocks UVB-induced MMP production and promotes type I procollagen synthesis via inhibition of MAPK/AP-1, NF-κβ and stimulation of TGF-β/Smad, Nrf2 in normal human dermal fibroblasts. J. Photochem. Photobiol. B Biol..

[211] Liu S., You L., Zhao Y., Chang X. (2018). Hawthorn Polyphenol Extract Inhibits UVB-Induced Skin Photoaging by Regulating MMP Expression and Type I Procollagen Production in Mice. J. Agric. Food Chem..

[212] Choi S.-I., Lee J.S., Lee S., Cho B.-Y., Choi S.-H., Han X., Sim W.-S., Kim Y.-C., Lee B.-Y., Kang I.-J. (2019). Protective Effects and Mechanisms of *Pourthiaea villosa* (Thunb.) Decne. Extract on Hydrogen Peroxide-Induced Skin Aging in Human Dermal Fibroblasts. J. Med. Food.

[213] You L., Kim M.-Y., Cho J.Y. (2021). Protective Effect of *Potentilla glabra* in UVB-Induced Photoaging Process. Molecules.

[214] Shin H.-S., Lee J.-M., Park S.-Y., Yang J.-E., Kim J.-H., Yi T.-H. (2013). Hair Growth Activity of *Crataegus pinnatifida* on C57BL/6 Mouse Model. Phytotherapy Res..

[215] Zhang R.-R., Meng N.-N., Liu C., Li K.-L., Wang M.-X., Lv Z.-B., Chen S.-Y., Guo X., Wang X.-K., Wang Q. (2020). PDB-1 from Potentilla discolor Bunge induces apoptosis and autophagy by downregulating the PI3K/Akt/mTOR signaling pathway in A549 cells. Biomed. Pharmacother..

[216] Tan Y.H., Shudo T., Yoshida T., Sugiyama Y., Si J.Y., Tsukano C., Takemoto Y., Kakizuka A. (2019). Ellagic acid, extracted from *Sanguisorba officinalis*, induces G1 arrest by modulating PTEN activity in B16F10 melanoma cells. Genes Cells.

[217] Shi C., Li Z., Wu Y., Li X., Li Y., Wei J., Li J., Zhang Y., Li L. (2020). Euscaphic acid and Tormentic acid protect vascular endothelial cells against hypoxia-induced apoptosis via PI3K/AKT or ERK 1/2 signaling pathway. Life Sci..

[218] Feng R., Ni H.-M., Wang S.Y., Tourkova I.L., Shurin M., Harada H., Yin X.-M. (2007). Cyanidin-3-rutinoside, a Natural Polyphenol Antioxidant, Selectively Kills Leukemic Cells by Induction of Oxidative Stress. J. Biol. Chem..

[219] Ma Z., Piao T., Wang Y., Liu J. (2015). Astragalin inhibits IL-1beta-induced inflammatory mediators production in human osteoarthritis chondrocyte by inhibiting NF-kappaB and MAPK activation. Int. Immunopharmacol..

[220] Huang W.C., Wu S.J., Tu R.S., Lai Y.R., Liou C.J. (2015). Phloretin inhibits interleukin-1beta-induced COX-2 and ICAM-1 expression through inhibition of MAPK, Akt, and NF-kappaB signaling in human lung epithelial cells. Food Funct..

[221] Liao H.R., Chen I.S., Liu F.C., Lin S.Z., Tseng C.P. (2018). 2’,3-dihydroxy-5-methoxybiphenyl suppresses fMLP-induced superoxide anion production and cathepsin G release by targeting the beta-subunit of G-protein in human neutrophils. Eur. J. Pharmacol..

[222] Khan K., Pal S., Yadav M., Maurya R., Trivedi A.K., Sanyal S., Chattopadhyay N. (2015). Prunetin signals via G-protein-coupled receptor, GPR30(GPER1): Stimulation of adenylyl cyclase and cAMP-mediated activation of MAPK signaling induces Runx2 expression in osteoblasts to promote bone regeneration. J. Nutr. Biochem..

[223] Suh S.J., Cho K.J., Moon T.C., Chang H.W., Park Y.G., Kim C.H. (2008). 3,4,5-trihydroxybenzaldehyde from Geum japonicum has dual inhibitory effect on matrix metalloproteinase 9; inhibition of gelatinoytic activity as well as MMP-9 expression in TNF-alpha induced HASMC. J. Cell Biochem..

[224] Park J.-B., Agnihotri S., Golbourn B., Bertrand K.C., Luck A., Sabha N., Smith C.A., Byron S., Zadeh G., Croul S. (2014). Transcriptional profiling of GBM invasion genes identifies effective inhibitors of the LIM kinase-Cofilin pathway. Oncotarget.

[225] Qin X., Xu X., Hou X., Liang R., Chen L., Hao Y., Gao A., Du X., Zhao L., Shi Y. (2022). The pharmacological properties and corresponding mechanisms of farrerol: A comprehensive review. Pharm. Biol..

[226] Yarar D., Waterman-Storer C.M., Schmid S.L. (2005). A Dynamic Actin Cytoskeleton Functions at Multiple Stages of Clathrin-mediated Endocytosis. Mol. Biol. Cell.

[227] Howell G.J., Holloway Z.G., Cobbold C., Monaco A.P., Ponnambalam S. (2006). Cell Biology of Membrane Trafficking in Human Disease. Int. Rev. Cytol..

[228] Konc J., Lešnik S., Janežič D. (2015). Modeling enzyme-ligand binding in drug discovery. J. Cheminform..

[229] Meng X.-Y., Zhang H.-X., Mezei M., Cui M. (2011). Molecular Docking: A Powerful Approach for Structure-Based Drug Discovery. Curr. Comput. Aided-Drug Des..

