# Supplementary material for: Modulation of Cytoskeleton, Protein Trafficking, and Signaling Pathways by Metabolites from Cucurbitaceae, Ericaceae, and Rosaceae Plant Families
Source: Pharmaceuticals (Basel). 2022 Nov 10;15(11):1380. doi: 10.3390/ph15111380 (PMC9698530; doi:10.3390/ph15111380)
Supplement: Supplementary file 1 [file pharmaceuticals-15-01380-s001.zip › Supplementary_Table_S1.pdf]

Supplementary Table S1. PubMed Search Terms and Results for the *Cucurbitaceae* Family.

Search Terms in PubMed	Results	Relevant	Notes	# of Relevant Papers: Elimination based on detailed paper review
**Cucurbitacin protein trafficking**	213	Too many irrelevant (plant-based mostly)	Therefore, focused the search to humans.	
**Cucurbitacin protein trafficking human**	8	6		5
**Cucurbitacin human motor protein**	1	Irrelevant		
**Cucurbitacin human dynein**	0			
**Cucurbitacin human kinesin**	0			
**Cucurbitacin human microtubules**	13	ALL Relevant	Many articles are overlapping within these categories	12
**Cucurbitacin human actin**	37	36 Relevant		28
**Cucurbitacin human vimentin**	3	ALL Relevant		3
**Cucurbitacin human lamin**	0			
**Cucurbitacin human golgi**	0			
**Cucurbitacin human endoplasmic reticulum**	3	ALL Relevant		2
**Cucurbitacin human lysosome**	5	ALL Relevant		3
**Cucurbitacin human signaling**	204	Did not assess (too many) - therefore focused search terms - see below	Many articles are overlapping within these categories	
**Cucurbitacin human MAPK**	28			18
**Cucurbitacin human AKT**	41			23
**Cucurbitacin human PI3K**	18			12
**Cucurbitacin human JAK**	30			17
**Cucurbitacin human STAT**	25			10
**Cucurbitacin human EGFR**	18			11
**Cucurbitacin human adenylyl cyclase**	1			1
**Cucurbitacin human phospholipase C**	0			
**Cucurbitacin human GPCR**	0			
