# Supplementary material for: Modulation of Cytoskeleton, Protein Trafficking, and Signaling Pathways by Metabolites from Cucurbitaceae, Ericaceae, and Rosaceae Plant Families
Source: Pharmaceuticals (Basel). 2022 Nov 10;15(11):1380. doi: 10.3390/ph15111380 (PMC9698530; doi:10.3390/ph15111380)
Supplement: Supplementary file 1 [file pharmaceuticals-15-01380-s001.zip › Supplementary_Table_S2.pdf]

Supplementary Table S2. PubMed Search Terms and Results for the *Ericaceae* Family.

Search Terms in PubMed	Results	Relevant	Notes	# of Relevant Papers: Elimination based on detailed paper review
**Ericaceae protein trafficking**	22	Many irrelevant	Mostly plant focused	
**Ericaceae protein trafficking human**	4			3
**Ericaceae human motor protein**	5	Irrelevant		
**Ericaceae human dynein**	0			
**Ericaceae human kinesin**	0			
**Ericaceae human microtubules**	1			1
**Ericaceae human actin**	4			2
**Ericaceae human vimentin**	2			2
**Ericaceae human lamin**	0			
**Ericaceae human golgi**	0			
**Ericaceae human endoplasmic reticulum**	2			2
**Ericaceae human lysosome**	0			
**Ericaceae human signaling**	121		Many and thus performed more focused searches below	
**Ericaceae human MAPK**	19		Overlap in some articles in these categories	18
**Ericaceae human AKT**	33			33
**Ericaceae human PI3K**	13			13
**Ericaceae human JAK**	3			3
**Ericaceae human STAT**	7			6
**Ericaceae human EGFR**	6			5
**Ericaceae human adenylyl cyclase**	0			
**Ericaceae human phospholipase C**	0			
**Ericaceae human GPCR**	0			
