# Supplementary material for: Modulation of Cytoskeleton, Protein Trafficking, and Signaling Pathways by Metabolites from Cucurbitaceae, Ericaceae, and Rosaceae Plant Families
Source: Pharmaceuticals (Basel). 2022 Nov 10;15(11):1380. doi: 10.3390/ph15111380 (PMC9698530; doi:10.3390/ph15111380)
Supplement: Supplementary file 1 [file pharmaceuticals-15-01380-s001.zip › Supplementary_Table_S3.pdf]

Supplementary Table S3. PubMed Search Terms and Results for the *Rosaceae* Family.

Search Terms in PubMed	Results	Relevant	Notes	# of Relevant Papers: Elimination based on detailed paper review
rosaceae protein trafficking	217		Plant focused	
rosaceae protein trafficking human	34			
rosaceae human motor protein	4	0	Irrelevant	
rosaceae human dynein	0			
rosaceae human kinesin	0			
rosaceae human microtubules	4	4		4
rosaceae human actin	13			12
rosaceae human vimentin	8			8
rosaceae human lamin	0			1
rosaceae human golgi	1			
rosaceae human endoplasmic reticulum	12			11
rosaceae human lysosome	6			3
rosaceae human signaling	324		Too many so utilize focused terms within this topic	
rosaceae human MAPK	48		Some overlap amongst these articles	47
rosaceae human AKT	49			48
rosaceae human PI3K	19			19
rosaceae human JAK	4			4
rosaceae human STAT	7			7
rosaceae human EGFR	16			16
rosaceae human adenylyl cyclase	1			1
rosaceae human phospholipase C	1			1
rosaceae human GPCR	3			3
